# Intraoperative electrocorticography using high-frequency oscillations or spikes to tailor epilepsy surgery in the Netherlands (the HFO trial): a randomised, single-blind, adaptive non-inferiority trial

**DOI:** 10.1016/S1474-4422(22)00311-8

**Published:** 2022-11

**Authors:** Willemiek Zweiphenning, Maryse A van 't Klooster, Nicole E C van Klink, Frans S S Leijten, Cyrille H Ferrier, Tineke Gebbink, Geertjan Huiskamp, Martine J E van Zandvoort, Monique M J van Schooneveld, M Bourez, Sophie Goemans, Sven Straumann, Peter C van Rijen, Peter H Gosselaar, Pieter van Eijsden, Willem M Otte, Eric van Diessen, Kees P J Braun, Maeike Zijlmans, HFO study group, Eltje M. Bloemen-Carlier, Veronika Cibulková, Renee de Munnink, Sandra van der Salm, Martinus J.C. Eijkemans, Janine M. Ophorst-van Eck, Anouk Velders, Charlotte J.J. van Asch, Jack Zwemmer, Renate van Regteren-van Griethuysen, Henriette Smeding, Lydia van der Berg, Jeroen de Bresser, Gérard A.P. de Kort, Jan-Willem Dankbaar

**Affiliations:** a Department of Neurology and Neurosurgery, Utrecht Brain Center, University Medical Center Utrecht (Part of ERN EpiCARE), Utrecht, Netherlands; b Department of Pediatric Psychology, Wilhelmina's Children Hospital, University Medical Center Utrecht, Netherlands; c Stichting Epilepsie Instellingen Nederland, Heemstede, Netherlands

## Abstract

**Background:**

Intraoperative electrocorticography is used to tailor epilepsy surgery by analysing interictal spikes or spike patterns that can delineate epileptogenic tissue. High-frequency oscillations (HFOs) on intraoperative electrocorticography have been proposed as a new biomarker of epileptogenic tissue, with higher specificity than spikes. We prospectively tested the non-inferiority of HFO-guided tailoring of epilepsy surgery to spike-guided tailoring on seizure freedom at 1 year.

**Methods:**

The HFO trial was a randomised, single-blind, adaptive non-inferiority trial at an epilepsy surgery centre (UMC Utrecht) in the Netherlands. We recruited children and adults (no age limits) who had been referred for intraoperative electrocorticography-tailored epilepsy surgery. Participants were randomly allocated (1:1) to either HFO-guided or spike-guided tailoring, using an online randomisation scheme with permuted blocks generated by an independent data manager, stratified by epilepsy type. Treatment allocation was masked to participants and clinicians who documented seizure outcome, but not to the study team or neurosurgeon. Ictiform spike patterns were always considered in surgical decision making. The primary endpoint was seizure outcome after 1 year (dichotomised as seizure freedom [defined as Engel 1A–B] *vs* seizure recurrence [Engel 1C–4]). We predefined a non-inferiority margin of 10% risk difference. Analysis was by intention to treat, with prespecified subgroup analyses by epilepsy type and for confounders. This completed trial is registered with the Dutch Trial Register, Toetsingonline ABR.NL44527.041.13, and ClinicalTrials.gov, NCT02207673.

**Findings:**

Between Oct 10, 2014, and Jan 31, 2020, 78 individuals were enrolled to the study and randomly assigned (39 to HFO-guided tailoring and 39 to spike-guided tailoring). There was no loss to follow-up. Seizure freedom at 1 year occurred in 26 (67%) of 39 participants in the HFO-guided group and 35 (90%) of 39 in the spike-guided group (risk difference –23·5%, 90% CI –39·1 to –7·9; for the 48 patients with temporal lobe epilepsy, the risk difference was –25·5%, –45·1 to –6·0, and for the 30 patients with extratemporal lobe epilepsy it was –20·3%, –46·0 to 5·4). Pathology associated with poor prognosis was identified as a confounding factor, with an adjusted risk difference of –7·9% (90% CI –20·7 to 4·9; adjusted risk difference –12·5%, –31·0 to 5·9, for temporal lobe epilepsy and 5·8%, –7·7 to 19·5, for extratemporal lobe epilepsy). We recorded eight serious adverse events (five in the HFO-guided group and three in the spike-guided group) requiring hospitalisation. No patients died.

**Interpretation:**

HFO-guided tailoring of epilepsy surgery was not non-inferior to spike-guided tailoring on intraoperative electrocorticography. After adjustment for confounders, HFOs show non-inferiority in extratemporal lobe epilepsy. This trial challenges the clinical value of HFOs as an epilepsy biomarker, especially in temporal lobe epilepsy. Further research is needed to establish whether HFO-guided intraoperative electrocorticography holds promise in extratemporal lobe epilepsy.

**Funding:**

UMCU Alexandre Suerman, EpilepsieNL, RMI Talent Fellowship, European Research Council, and MING Fund.

## Introduction

Epilepsy surgery is the only potentially curative treatment for people with focal—and presumed structural—epilepsy. The seizure freedom rate after epilepsy surgery is 36–84% after 1 year, and approximately 60% after 5 years or more.^1^ Intraoperative electrocorticography can be performed during surgery to map functional regions and to optimise the delineation of epileptogenic tissue by analysing interictal spikes or spike patterns. This so-called tailoring can affect surgical decision making.^2^ Spikes are defined as paroxysmal sharp transients in the conventional EEG frequency range between 0–80 Hz, with a maximum duration of 80 ms and minimal amplitude of twice the baseline. Ictiform spike patterns are defined as rhythmic patterns or discharges with increasing amplitude and decreasing frequency.^3^ Complete resection of areas showing interictal spikes and ictiform spike patterns has been associated with good seizure outcome.^3^, ^4^, ^5^ Some studies suggest that residual spikes after resection predict a poor outcome, but other studies contradict this finding.^6^, ^7^, ^8^, ^9^, ^10^

**Research in context**

**Evidence before this study**

We searched PubMed, Embase, the Cochrane library, and the ClinicalTrials.gov registry on July 5, 2021, without language restrictions, to identify randomised controlled trials, other clinical trials, meta-analyses, and systematic reviews comparing high frequency oscillations (HFOs) and spikes for delineation of the epileptogenic tissue in epilepsy surgery, published between database inception and July 5, 2021. We used the search terms “high frequency oscillations” AND “epilepsy” OR “epilepsy surgery”. We identified one meta-analysis, one Cochrane review, one prospective observational multicentre study, and three ongoing clinical trials. The meta-analysis, including 11 studies, focused on existing retrospective evidence of the relation between resection of HFO-generating areas on mainly extraoperative invasive EEG and surgical outcome. The findings of the meta-analysis showed a difference in HFO resection ratios between seizure-free and seizure recurrence outcome groups (ripples: difference 0·18; 95% CI 0·10–0·27; fast ripples: difference 0·17; 0·01–0·33). The effect sizes in this meta-analysis were small but favoured HFOs. The Cochrane review included two prospective observational studies (including 11 patients in total) in which ictal HFOs were recorded during long-term electrocorticography, reporting on outcomes of epilepsy surgery. The quality of evidence for all outcomes (six patients were seizure-free [Engel 1]) was very low. The prospective observational multicentre clinical study evaluated the use of extraoperative and intraoperative interictal HFOs to predict seizure outcome. 52 patients from three tertiary epilepsy centres were included over 1 year. On an individual patient level, prediction of outcome was impossible in all patients. However, HFOs predicted outcomes better in the group using intraoperative recordings in children than in the extraoperative invasive EEG recordings in adults. All described prospective studies were relatively small non-randomised trials, without control groups or masking. None of the studies reported adverse effects. At the time of our search, ClinicalTrials.gov listed three ongoing clinical studies: one observational study of intraoperative electrocorticography in patients with tumours and epilepsy (NCT02320136, n=120), one randomised controlled trial comparing HFOs and spikes versus spikes in extraoperative and intraoperative electrocorticography to predict outcome in paediatric refractory epilepsy (NCT03790280, n=30), and our study. No previous studies have compared surgical results guided by HFOs versus those not considering HFOs.

**Added value of this study**

This study is the first randomised controlled trial of HFO-based intraoperative electrocorticography-tailoring in clinical practice. This trial was needed to test whether decision making based on HFOs is as useful as that based on spikes, and does not lead to worse seizure outcomes.

**Implications of all the available evidence**

No reliable conclusions on the value of HFOs for tailoring epilepsy surgery can be drawn from the evidence that is available, including that from the HFO trial. The HFO randomised trial did not show non-inferiority of HFOs to spikes in intraoperative electrocorticography. After confounder correction, non-inferiority of HFOs to spikes was noted in extratemporal lobe epilepsy. The superiority of spikes was suggested by our findings in the whole population and in temporal lobe epilepsy. The results of the HFO trial warn against clinical interpretation of HFOs in the temporal lobes and provide new insights for the design of future randomised controlled trials in epilepsy surgery, in particular inclusion, exclusion, and stratification criteria.

Over the past two decades, high-frequency oscillations (HFOs) have been proposed as a new precise biomarker for delineation of epileptogenic tissue.^11^ HFOs are transient bursts of activity above the conventional EEG frequency range. HFOs are subdivided into ripples (80–250 Hz) and fast ripples (250–500 Hz).^12^ Retrospective studies show that, at a group level, localisation of HFOs correlates better with the seizure onset zone and postsurgical outcome than localisation of spikes.^12^, ^13^, ^14^, ^15^, ^16^ Residual HFOs after resection, in particular fast ripples, predict seizure recurrence.^13^, ^14^ In addition, automatic detection by a computer algorithm is easier for HFOs than for spikes, which facilitates the process towards automatisation of the surgical guidance using intraoperative electrocorticography. We conducted the HFO trial to investigate, prospectively, whether tailoring of epilepsy surgery guided by HFOs in the intraoperative electrocorticogram was non-inferior to tailoring guided by spikes in terms of seizure outcome 1 year after surgery. Clinical information—including MRI, semiology, and ictiform spike patterns—was also incorporated into surgical decision making.

## Methods

### Study design and participants

The HFO trial was a randomised controlled, single-blind adaptive non-inferiority trial conducted at the UMC Utrecht. This is one of the three university medical centres in the Netherlands at which epilepsy surgery is performed. It specialises in paediatric epilepsy surgery. The other two centres are the VUmc Amsterdam and Maastricht UMC. Initially, two of the three epilepsy surgery centres agreed to participate in the trial. VUmc Amsterdam, which only operates on adults, withdrew participation shortly after the start of the trial, because of a change in the national referral policy. No individuals from VUmc Amsterdam were included in the trial. The third university medical centre, Maastricht UMC, did not participate.

Participants were candidates for epilepsy surgery of all ages referred to the Dutch Collaborative Epilepsy Surgery Program or the UMC Utrecht SEIN epilepsy surgery programme and selected to undergo intraoperative electrocorticography-guided epilepsy surgery. Members of the Dutch Collaborative Epilepsy Surgery Program come from two Dutch epilepsy referral centres (Stichting Epilepsie Instellingen Nederland, Utrecht; and Kempenhaeghe, Heeze) and the three Dutch university medical centres at which epilepsy surgery is performed (UMC Utrecht, VUmc Amsterdam, and Maastricht UMC). Both programmes excluded patients undergoing primary tumour surgery, even with intraoperative electrocorticography.

Exclusion criteria were a presumed occipital focus, to avoid physiological fast ripples; chronic invasive EEG monitoring before surgery, because this procedure would provide information on invasively recorded spikes and HFOs; and insufficient command of the Dutch language to complete questionnaires. Previous epilepsy surgery was not an exclusion criterion. Due to a clinical policy shift towards early surgery, individuals who did not yet fulfil the International League Against Epilepsy criteria for refractory epilepsy—at least two seizures in the past 24 months despite previous use of two or more different, well tolerated antiseizure medications—could be included by a protocol amendment made in July, 2015 (appendix pp 3–4).

The trial protocol and rationale for the trial have been published previously.^2^ The medical committee at UMC Utrecht approved the trial protocol (MEC-13389). The trial was run on the principles of the Declaration of Helsinki. All individuals and the parents or legal representatives of individuals who were younger than 18 years or incapacitated provided written informed consent before enrolment.

### Randomisation and masking

A data manager independent of the trial team generated an online permuted block randomisation scheme (block sizes of two, four, and six) that was stratified by epilepsy type (temporal lobe epilepsy *vs* extratemporal lobe epilepsy) and by study site (UMC Utrecht *vs* VUmc Amsterdam), and this scheme was uploaded into a secure database (ALEA version 2.2). Stratification by study site would also have included indirect stratification by age, because both children and adults can have surgery at UMC Utrecht whereas VUmc Amsterdam only operates on adults. However, due to the premature closure of the second site before any participants were enrolled, indirect stratification for age was not done. On the day before surgery, participants underwent a baseline neurological examination (National Institutes of Health Stroke Scale) and completed questionnaires on seizure frequency, antiseizure medication use, and quality of life, then they were randomly assigned (1:1) to either HFO-guided or spike-guided intraoperative electrocorticography-tailored surgery by the coordinating researchers (MZ, MAvtK, WZ, NECvK). The random allocation was communicated to the neurosurgeon by one of the coordinating researchers on the day before surgery. Treatment allocation was masked to the participants and physicians involved in the follow-up in outpatient clinics who documented seizure outcome, but not to the study team or neurosurgeon (PE, PG, PR). The intraoperative electrocorticography and neurosurgery reports that were uploaded to the clinical system were masked for treatment allocation. These original reports were uploaded to a separate system, which only the study team had access to. The original intraoperative electrocorticography reports replaced the masked reports after study completion.

### Procedures

All participants who were referred for intraoperative electrocorticography-tailored epilepsy surgery received standard perisurgical care (ie, all care and clinical procedures during surgery). Participants underwent an extensive presurgical workup, starting with MRI, video-EEG, and a neuropsychological examination. If deemed necessary by the Dutch Collaborative Epilepsy Surgery Program or the UMC Utrecht SEIN epilepsy surgery programme, language studies (ie, studies to test hemisphere performance or dominance), EEG-functional MRI, magnetoencephalogram, high-resolution EEG, 7T MRI, PET, or ictal SPECT could also be done. The surgical plan and initial placement of electrodes were based on this presurgical workup.

Clinical practice at UMC Utrecht is that intraoperative electrocorticography is generally performed only in patients with lesional epilepsy, concordant results of non-invasive investigations, and an epileptic focus (ie, the region were the epilepsy is presumed to originate from) outside of functional eloquent brain regions. Intraoperative electrocorticography is done to determine the extent of the neocortical resection that is needed, or it refines a decision on the necessity of a hippocampectomy. Information from the electrocorticography is weighed against other clinical information, such as MRI, video-EEG, semiology, and neuropsychological testing. Recording an ictiform spike pattern (ie, rhythmic spikes or an electrographic seizure) would provide more weight in a decision about the resection compared with recording non-rhythmic spikes, but non-rhythmic spikes are also considered when planning. A clear focus of spikes that persist after the initial resection might be a reason to extend the resection. The abundance of spikes from mesiotemporal structures affects the decision about hippocampectomy. The irritative zone might direct surgical entry and extent. All information is discussed within the surgical team. The advice of the clinical neurophysiologist is generally followed by the neurosurgeon, considering functional and anatomical constraints.

Intraoperative electrocorticography was recorded for both the spike-guided and HFO-guided groups using combinations of 2 *×* 4, 4 *×* 4, or 4 *×* 5 electrode grids and 1 *×* 6 or 1 *×* 8 electrode strips or needles (Ad-Tech, Racine, WI, USA; DIXI Medical, Chaudefontaine, France) placed directly on the cortex. Recordings were made with a 64-channel (MicroMed LTM Express, MicroMed, Veneto, Italy) or 32-channel (MicroMed Flexi, MicroMed, Veneto, Italy) EEG-system at 2048 Hz sampling rate. The signal was referenced to an external electrode placed on the mastoid. Propofol infusion, which was used to sedate patients during surgery, was temporarily interrupted during recording. Once a continuous intraoperative electrocorticography background pattern was reached, we recorded the signals for at least 2 min before moving the electrode grid to the next position.

The intraoperative electrocorticography signals were always analysed by a specialist clinician assistant and clinical neurophysiologist (TG, CF, and FL) in conventional EEG filter settings of 0·5–70 Hz, an amplification of 75–100 μV/mm, and a timescale of 10–15 s/page for ictiform spike patterns. For participants who were randomly allocated to the spike-guided group, the specialist clinician assistant and clinical neurophysiologist also analysed the intraoperative electrocorticography signals for non-ictiform spikes. For participants who were randomly allocated to the HFO-guided group, the intraoperative electrocorticography recordings were transferred to a separate computer and analysed by two of the HFO researchers per surgery (MAvtK, NECvK, MZ, and WZ) who were unaware of the conventionally recorded electrocorticography signals (ie, spikes and ictiform spike patterns, which are seen using frequency ranges that are different from those used to visualise HFOs). The separate computer was needed because the clinical software did not have the specified requirements to enable visual HFO analysis of the signal and because the clinical neurophysiologist needed to look for ictiform spike patterns at the same time. The intraoperative electrocorticography signals from the HFO guidance group were transferred to Stellate Harmonie Reviewer (version 7.0; Montreal, QC, Canada) for HFO analysis with the following settings: for ripples, a finite impulse response filter (>80 Hz, 5 μV/mm gain, and 0·4 s per page); and for fast ripples, a finite impulse response filter (>250 Hz, 1 μV/mm gain, and 0·4 s per page). HFO findings were discussed with the clinical neurophysiologist in charge. No automated HFO detector was used, because when the HFO trial began only offline detectors existed.

Based on the biomarker findings (either spikes or HFOs), advice was given by the clinical neurophysiologist to the neurosurgeon on the extent of the tissue to be resected in relation to the surgical plan. Independent of the random allocation, ictiform spike patterns were always analysed. If an ictiform spike pattern showed HFOs on some channels, these channels were considered most relevant and removed first. In the absence of any relevant events, the resection was performed—as planned—on the basis of the presurgical hypothesis of the epileptogenic zone.

Postoperatively, individuals in both treatment groups received the same clinical care, including a standardised neurological examination before discharge after surgery. In the case of new or unexpected postsurgical neurological deficits, the neurological examination was repeated at the clinical follow-up visits at 6–8 weeks, 6 months, or 12 months after surgery.

### Outcomes

The primary endpoint was seizure outcome 1 year after surgery (assessed by Engel classification). Seizures occurring within 2 weeks post-surgery were not included in the primary outcome assessment. Seizure outcome was dichotomised to seizure freedom (Engel 1A–B) versus seizure recurrence (Engel 1C–4). We decided to include individuals with auras (Engel 1B) in the seizure-free group because it can be difficult to distinguish true auras (focal aware seizures) from aura-like non-epileptic sensations. Postsurgical outcomes at 6–8 weeks, 6 months, and 12 months were determined through a follow-up questionnaire completed by the individual or legal representative and the treating clinician, who was masked to the randomly allocated treatment group, at the standard clinical care follow-up visits.

We assessed six secondary endpoints. First was the duration of surgery and intraoperative electrocorticography recording time. Second was the resection volume (based on MRI voxel-based volumetry). Third was change in cognitive function (divided into four domains: overall cognitive functioning, working memory, processing speed, and memory consolidation) at 1 year after surgery compared with presurgical neurophysiological assessment, expressed as negative, no change, or positive change (for details see appendix p 5). Fourth was quality of life before and after surgery (at 6–8 weeks, 6 months, and 12 months), using a visual analogue scale (range 0–10) on overall self-perceived quality of life. Fifth was new neurological deficits at hospital discharge by comparing the National Institutes of Health Stroke Scale before discharge with the presurgical score. Sixth was serious adverse events, defined as any medical occurrence that resulted in death, was life-threatening, required hospitalisation, or resulted in disability during the first year after surgery.

### Statistical analysis

On the basis of findings of previous studies,^12^, ^15^, ^16^ we estimated the chance of seizure freedom at 1 year after intraoperative electrocorticography-tailored surgery to be 65% when tailoring was based on spikes, versus 80% when tailoring was based on HFOs.^2^ Using a power of 80%, a one-sided significance level (α) of 0·05 (thus a 90% CI), and a non-inferiority margin (δ) of 10%, based on non-inferiority drug trials in epilepsy and the ranges of success in epilepsy surgery, we calculated that 78 individuals (39 per treatment group) would be needed to determine non-inferiority of HFO-based tailoring to spike-based tailoring on intraoperative electrocorticography. We did not consider clustering or the interim analyses for the sample size calculation.

We present summary statistics as absolute and relative frequencies for categorical data and mean (SD) or median (IQR) for continuous data. Analysis of the primary outcome was done in both the intention-to-treat population (defined as all participants who were assessed according to the random allocation) and the per-protocol population (defined as all participants according to the treatment received) Secondary outcomes were analysed in the intention-to-treat population only. Safety was analysed in all randomised patients.

Differences between categorical variables are presented as risk differences (90% CI; logistic regression) and compared using χ or Fisher's exact tests. Differences between continuous data are presented as differences between means (90% CI; compared using unpaired t-tests) or medians (90% CI; compared using non-parametric Wilcoxon tests). We prespecified subgroup analyses of the primary outcome by epilepsy type. We also prespecified a test for potential confounders of seizure outcome (ie, auras, bilateral tonic-clonic seizures, underlying pathology, pathology prognosis [good *vs* poor^1^], age at surgery, epilepsy duration, number of preoperative investigations, lobe of surgery, previous brain surgery, and treating neurosurgeon) using univariable followed by multivariable logistic regression and calculating the odds ratio (OR) and 90% CI. Variables in the univariable logistic regression that showed a p value of less than 0·20 with respect to seizure outcome were included in the multivariable logistic regression analysis. We corrected for any confounding factors resulting from the multivariable logistic regression that had a p value of less than 0·05 in subsequent prespecified analyses, overall and by epilepsy type.^1^ The intraclass correlation for neurosurgeons was calculated to check for potential clustering. We did univariable linear regression analysis of secondary outcomes with respect to seizure outcome and used an imputed dataset to correct for missing data. Imputation was done using the method of multiple imputations by chained equations (MICE package R; default settings), using predictive mean matching with 20 imputation sets.^17^ Regression results were pooled according to the Rubin rule. For neuropsychological data, linear regression analysis was performed for participants with a complete set of data only, because imputation could not be done due to the high amount of missing data. We applied no bias adjustment. We considered p values less than 0·05 significant for all analyses except the planned interim analyses. The original statistical analysis plan is in the study protocol (appendix pp 6–43).

An independent data monitoring committee (IDMC) assessed safety and efficacy after the first 20 (April, 2016) and 40 (April, 2017) participants had been enrolled. These interim analyses, performed by the IDMC biostatistician, included a pre-planned analysis of an anonymised dataset with preliminary postsurgical outcomes (6–8 weeks, 6 months, and 12 months) and included the number of serious adverse events. An efficiency analysis was performed after 40 participants had been included, using a one-sided alpha-correction of 0·003 (O’Brien Fleming method). Premature termination would be indicated if harmful effects were reported in the HFO-guided group compared with the spike-guided group. Researchers were masked to findings of the interim analyses to minimise operational bias. No reason was found to terminate the trial prematurely because of harmful effects, superiority, or non-feasibility issues, and no amendments to the study protocol were made at the request of the IDMC. The efficiency analysis yielded a p value of 0·62 (appendix pp 44–59).

Anonymised data collection, management, and storage were done in open-source clinical trial software OpenClinica (OpenClinica, Waltham, MA). Follow-up questionnaires were sent and processed through NetQ, a medical certified online survey platform. Analyses were done with the statistical software RStudio, version 1.3.1093. Data monitoring was performed twice a year by an independent clinical research associate (Julius Clinical, Zeist, Netherlands).

This trial is registered with the Dutch Trial Register, Toetsingonline ABR.NL44527.041.13, and ClinicalTrials.gov, NCT02207673.

### Role of the funding source

The funders had no role in study design, data collection, data analysis, data interpretation, writing of the report, or the decision to submit the paper for publication.

## Results

**Figure 1:**
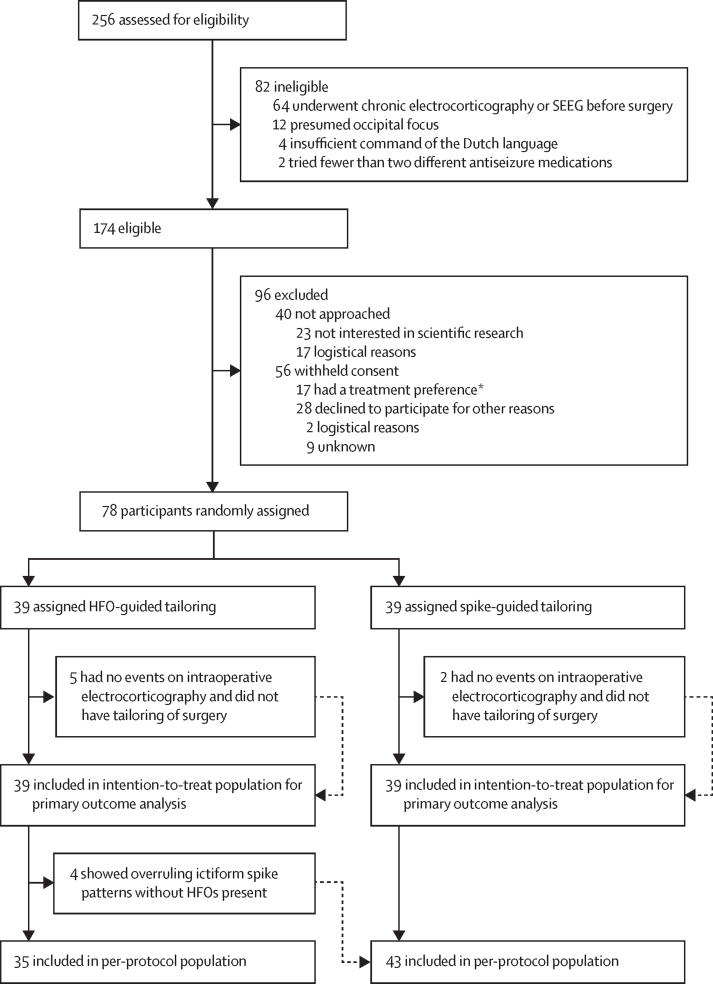
Trial profile HFOs=high frequency oscillations. SEEG=stereo-electroencephalography. *13 withheld consent because of the risk of the resection being too small, and four withdrew consent because of the uncertainty of the treatment assignment.

Between Oct 10, 2014, and Jan 31, 2020, 256 patients referred for intraoperative electrocorticography-tailored epilepsy surgery at UMC Utrecht were screened for eligibility (figure 1). 82 participants were excluded, mostly because they underwent invasive EEG monitoring before surgery. A further 40 participants were not approached to participate, and 56 individuals withheld consent (appendix p 60). Therefore, 78 participants were enrolled and randomly assigned, 39 to HFO-guided surgery and 39 to spike-guided surgery, comprising the intention-to-treat population. Age at surgery and the number of adults was higher in the HFO-guided group than in the spike-guided group (table 1). More auras were reported in the spike-guided group than in the HFO-guided group, and pathology associated with poor outcome was more frequent in the HFO-guided group than in the spike-guided group.

**Table 1:** Baseline characteristics

		**HFO group (n=39)**	**Spike group (n=39)**
**Sex**			
Female		18 (46%)	20 (51%)
Male		21 (54%)	19 (49%)
**Age at surgery, years**			
Median		21 (12–39)	15 (9·2–29·0)
Chil (aged 0–16 years)		17 (44%)	22 (56%)
Adult (aged >16 years)		22 (56%)	17 (44%)
**Epilepsy duration, years**			
Median		10·0 (2·8–22·0)	7·9 (2·2–14·0)
**Auras in the past month**			
Yes		13 (33%)	23 (59%)
No		26 (67%)	16 (41%)
Median		10·0 (4·0–15·0)	5·0 (2·0–22·0)
**Bilateral tonic-clonic seizures**			
Yes		13 (33%)	11 (28%)
**Seizures in the past month**			
Yes		38 (97%)	36 (92%)
No		1 (3%)	3 (8%)
Median		12 (3·0–75·0)	15 (4·0–80·0)
**Number of ASMs tried**			
<2		8 (21%)	15 (38%)
Median		2·0 (2·0–2·5)	2·0 (1·0–3·0)
**Number of ASMs used at intervention**			
0		0	2 (5%)
1		8 (21%)	13 (33%)
2		21 (54%)	11 (28%)
3		6 (15%)	10 (26%)
4		3 (8%)	3 (8%)
5		1 (3%)	0
**Neurosurgeon**			
A		14 (36%)	11 (28%)
B		21 (54%)	22 (56%)
C		4 (10%)	5 (13%)
**Surgery side**			
Left		19 (49%)	19 (49%)
Right		20 (51%)	20 (51%)
**Site of surgery**			
Frontal		14 (36%)	10 (26%)
Fronto-temporo-parietal		0	1 (3%)
Parietal		0	4 (10%)
Temporal		24 (62%)	24 (62%)
	Anterior lobectomy	14 (36%)	15 (38%)
	Complete lobectomy	2 (5%)	2 (5%)
	Neocortical resection	8 (21%)	7 (18%)
	Temporo-parieto-occipital	1 (3%)	0
**Previous brain surgery**			
Yes		5 (13%)	1 (3%)
No		34 (87%)	38 (97%)
**Number of preoperative investigations**			
>3		15 (38%)	11 (28%)
Median		2·0 (2·0–3·0)	2·0 (2·0–3·0)
**Preoperative MRI**			
Abnormal*		29 (74%)	30 (77%)
Abnormal on revision†		7 (18%)	8 (21%)
Normal‡		3 (8%)	1 (3%)
**Preoperative 7T MRI done**			
Yes		8 (21%)	4 (10%)
No		31 (79%)	35 (90%)
**Preoperative investigations**			
MRI			
	Concordant with resection	30 (77%)	34 (87%)
	Partly discordant with resection	9 (23%)	5 (13%)
	Not performed	0	0
Interictal EEG			
	Concordant with resection	23 (59%)	26 (67%)
	Partly discordant with resection	16 (41%)	11 (28%)
	Not performed	0	2 (5%)
Ictal EEG			
	Concordant with resection	22 (56%)	25 (64%)
	Partly discordant with resection	9 (23%)	7 (18%)
	Not performed	8 (21%)	7 (18%)
PET			
	Concordant with resection	4 (10%)	4 (10%)
	Partly discordant with resection	6 (15%)	5 (13%)
	Not performed	29 (74%)	30 (77%)
SPECT			
	Concordant with resection	2 (5%)	1 (3%)
	Partly discordant with resection	0	1 (3%)
	Not performed	37 (95%)	37 (95%)
Magnetoencephalography			
	Concordant with resection	4 (10%)	1 (3%)
	Partly discordant with resection	2 (5%)	3 (8%)
	Not performed	33 (85%)	35 (90%)
**Good prognosis pathology**§			
Total		25 (64%)	34 (87%)
Tumour		10 (26%)	16 (41%)
Vascular malformation		4 (10%)	3 (8%)
Hippocampal sclerosis		2 (5%)	6 (15%)
FCD 2		9 (23%)	9 (23%)
**Poor prognosis pathology**§			
Total		14 (36%)	5 (13%)
Gliosis; reactive tissue		6 (15%)	1 (3%)
Tuber (tuberous sclerosis)		5 (13%)	1 (3%)
FCD 1 and mild MCD		1 (3%)	2 (5%)
No abnormality		2 (5%)	1 (3%)

Data are n (%) or median (IQR). ASM=anti-seizure medication. FCD=focal cortical dysplasia. MCD=malformations of cortical development.

* Abnormalities seen after the first assessment by a neuroradiologist.

† Subtle abnormalities only recognised after revision of the MRI together with other modalities.

‡ No abnormalities.

§ Pathology prognosis (ie, good and poor) is based on the definition of Lamberink and colleagues (2020).^1^

**Figure 2:**
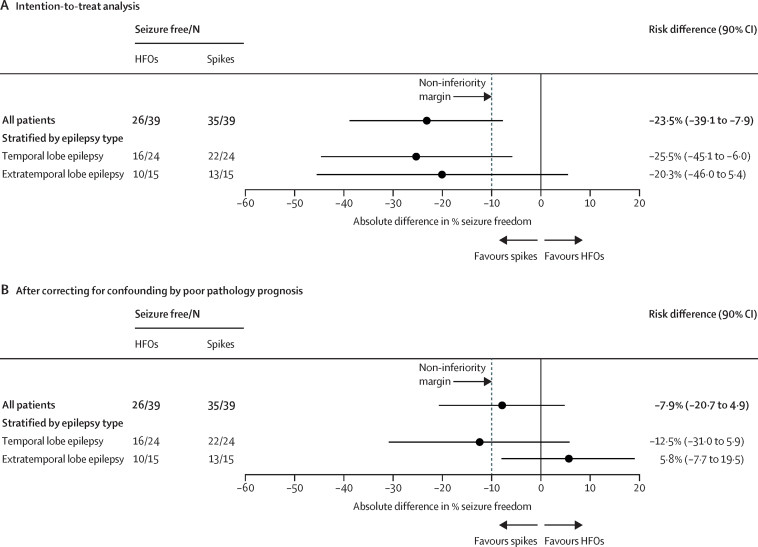
Forest plot of the primary analysis and prespecified subgroup analyses Results are absolute risk differences (dots) with a 90% CI (line) for the primary endpoint seizure freedom. Non-inferiority of tailoring based on HFOs would be shown if the lower limit of the 90% CI of the absolute risk difference was higher than the –10% non-inferiority margin (dotted line). HFOs=high frequency oscillations.

No participants were lost to follow-up (follow-up was 1 year for all patients). The primary endpoint analysis done at 1 year after surgery showed that 26 (67%) of 39 individuals in the HFO-guided group and 35 (90%) of 39 individuals in the spike-guided group were seizure-free (risk difference –23·5%, 90% CI –39·1 to –7·9; figure 2A). In the prespecified analysis by type of epilepsy, the risk difference for the subgroup of 48 patients with temporal lobe epilepsy was –25·5% (90% CI –45·1 to –6·0), and for the subgroup of 30 patients with extratemporal lobe epilepsy, the risk difference was –20·3% (90% CI –46·0 to 5·4). These findings all indicated that non-inferiority of HFOs was not proven (the lower bound of each 90% CI was lower than –10%). In the prespecified analysis of confounding variables, a significant association was found between poor prognosis pathology and seizure outcome (OR 11·89 [90% CI 3·17 to 44·56], p=0·0002; appendix pp 61–64). After correcting for the confounder of poor prognosis pathology, adjusted risk differences were –7·9% (90% CI –20·7 to 4·9) for the whole patient group, –12·5% (–31·0 to 5·9) for temporal lobe epilepsy, and 5·8% (–7·7 to 19·5) for extratemporal lobe epilepsy (figure 2B). The intraclass correlation for neurosurgeons was 0·17, indicating a low clustering effect (appendix p 65).

**Figure 3:**
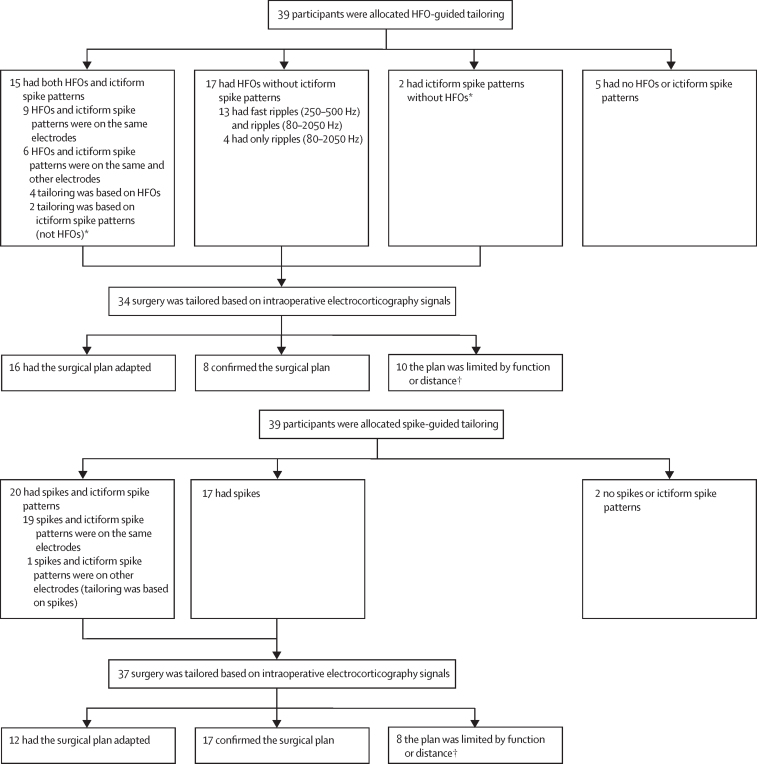
Intraoperative electrocorticography tailoring of surgery Flowcharts show findings of intraoperative electrocorticography during the surgical procedure for each treatment group and the effect on surgical decision making. HFOs=high frequency oscillations. *Included in per-protocol analysis. †Limited by function means that the extent of surgery was limited due to eloquent cortex; limited by distance means that the surgery was not extended because the identified EEG events were too distant from the surgical area.

For 71 (91%) of 78 participants, intraoperative electrocorticography-tailoring led to an adaptation or confirmation of the initial surgical plan, sometimes limited by eloquent regions or distance to resection (figure 3). In five individuals in the HFO-guided group and two in the spike-guided group, we did not record any HFOs or spike patterns (figure 1). We recorded ictiform spike patterns without co-occurring HFOs in four individuals who had been assigned to HFO-guided tailoring. Therefore, in the per-protocol population, these participants were analysed as part of the spike-guided group (appendix pp 66–71).

Secondary endpoints did not differ between the HFO-guided and spike-guided groups (table 2). Univariable linear regression of the imputed dataset for secondary outcome variables showed that intraoperative electrocorticography recording time (OR 0·99 [90% CI 0·98–1·00], p=0·03), pre-existing neurological deficits (0·77 [0·63–0·94], p=0·04), and postoperative quality of life (1·08 [1·03–1·14], p=0·01) were associated with seizure outcome. Univariable linear regression analysis of the neuropsychological assessments of the participants with a complete set of data showed no differences between the two treatment groups in the four domains of cognitive change (overall cognitive functioning, working memory, processing speed, and memory consolidation) related to seizure outcome.

**Table 2:** Secondary outcomes

	**HFO group (n=39)**	**Spike group (n=39)**	90% of absolute difference*or risk difference†	**p value (one-sided)**
**Total duration of surgery, min**				
Mean (SD)	240 (74)	240 (76)	0 (−23·1 to 33·4)*‡	0·76‡
Missing	0	0	N/A	N/A
**Total duration of intraoperative electrocorticography tailoring, min**				
Median (IQR)	22 (18–34)	22 (17–29)	0 (−1·2 to 6·6)*‡	0·25‡
Missing	0	0	N/A	N/A
**Resection volume, cm^3^**				
Median (IQR)	20 (5·7–31)	14 (6·2–20)	6 (−1·4 to 12·2)*†	0·19‡
Missing	11	12	N/A	N/A
**Preoperative quality of life**§				
Median (IQR)	7 (5·8–8)	7 (6–9)	0 (−1·1 to 0·4)*‡	0·47‡
Missing	0	0	N/A	N/A
**Postoperative quality of life**§**at 8 weeks**				
Median (IQR)	8 (7–8)	8 (7–9)	0 (−0·6 to 0·6)*‡	0·99‡
Missing	0	1	N/A	N/A
**Postoperative quality of life**§**at 6 months**				
Median (IQR)	8 (8–9)	8 (7–10)	0 (−0·6 to 0·6)*‡	0·95‡
Missing	1	0	N/A	N/A
**Postoperative quality of life**§**at 1 year**				
Median (IQR)	8 (7–10)	9 (8–9)	1 (−0·8 to 0·4)*‡	0·52‡
Missing	0	2	N/A	N/A
**Postoperative cognitive change**¶**—overall cognitive functioning**				
No change	18 (46%)	18 (46%)	1 (ref)†	0·27‖
Negative change	3 (8%)	7 (18%)	−0·14 (−0·37 to 0·10)†	..
Positive change	6 (15%)	8 (21%)	−0·06 (−0·31 to 0·19)†	..
Missing	12 (31%)	6 (15%)	..	..
**Postoperative cognitive change**¶**—processing speed**				
No change	7 (18%)	12 (31%)	1 (ref)†	0·60‖
Negative change	11 (28%)	8 (21%)	0·21 (−0·10 to 0·53)†	..
Positive change	7 (18%)	6 (15%)	0·17 (−0·18 to 0·52)†	..
Missing	14 (36%)	13 (33%)	N/A	N/A
**Postoperative cognitive change**¶**—working memory**				
No change	7 (18%)	12 (31%)	1 (ref)†	0·30‖
Negative change	6 (15%)	9 (23%)	0·03 (−0·29 to 0·35)†	..
Positive change	6 (15%)	3 (8%)	0·26 (−0·10 to 0·62)†	..
Missing	20 (51%)	15 (38%)	N/A	N/A
**Postoperative cognitive change**¶**—memory consolidation**				
No change	5 (13%)	8 (21%)	1 (ref)†	0·68‖
Negative change	5 (13%)	7 (18%)	0·03 (−0·34 to 0·40)†	N/A
Positive change	6 (15%)	5 (13%)	0·16 (−0·26 to 0·58)†	..
Missing	23 (59%)	19 (49%)	N/A	N/A
Pre-existing neurological deficit**				
No deficits	30 (77%)	34 (87%)	1 (ref)†	0·38‖
Deficits	9 (23%)	5 (13%)	0·10 (−0·06 to 0·27)†	..
Missing	0	0	N/A	N/A
Postoperative neurological deficits at discharge**††				
No or resolved deficits	31 (79%)	32 (82%)	1 (ref)†	1·0‖
Deficits	8 (21%)	7 (18%)	0·05 (−0·09 to 0·19)†	..
Missing	0	0	N/A	N/A
**Serious adverse events**				
No serious adverse events	34 (87%)	36 (92%)	1 (ref)†	0·71‖
Serious adverse events	5 (13%)	3 (8%)	0·05 (−0·09 to 0·19)†	..
Missing	0	0	N/A	N/A

* 90% of absolute difference.

† Risk difference.

‡ t-test.

§ Quality of life was determined using a visual analogue scale (0–10; with 0 being worst and 10 being best).

¶ Postoperative cognitive change is calculated as the Z score of the difference between pre-test and post-test results; categorised as negative change (Z score <−0·5), no change (Z score −0·5 to 0·5), and positive change (Z score >0·5).

‖ test.

** Neurological deficits determined using the National Institutes of Health Stroke Scale.

†† Postoperative neurological deficits at discharge contained (partly) anticipated neurological deficits in 11 patients, including visual field deficits in seven patients not subject to follow-up. Neurological follow-up was done in eight patients (deficits healed over time in five [63%] of these patients) at 8 weeks, two patients (two [100%]) at 6 months, and one patient (one [100%]) at 1 year.

Eight serious adverse events were recorded, five in the HFO-guided group and three in the spike-guided group (table 3). These serious adverse events were resolved after prolonged or additional hospitalisation, and three (one in the HFO-guided group and two in the spike-guided group) were considered directly related to the surgery. No patients died.

**Table 3:** Serious adverse events

	**Allocated group**	**Outcome at 1 year**
Readmittance (5 days) due to nausea and headache	HFOs	Seizure free (Engel 1A–B)
Status epilepticus after surgery	Spikes	Seizure free (Engel 1A–B)
Temporary neurological deficit in left arm without EEG correlate	HFOs	Seizure free (Engel 1A–B)
Subdural haematoma, external hydrocephalus, urosepsis	Spikes	Seizure free (Engel 1A–B)
CSF leakage from the wound	HFOs	Seizure free (Engel 1A–B)
Attempted suicide (non-fatal)	HFOs	Seizure free (Engel 1A–B)
Hospitalisation for an increase in seizure frequency after surgery*	Spikes	Seizure free (Engel 1A–B)
Hospitalisation for an increase in seizure frequency after surgery	HFOs	Recurrent seizures (Engel 1C–4)

Each row consists of the serious adverse event encountered for an individual patient. Serious adverse events were assessed in the safety population (ie, all included patients, n=78); all patients with a serious adverse event required hospitalisation. HFO=high-frequency oscillations.

* Any seizures occurring within 2 weeks post-surgery were not included in the primary outcome assessment.

## Discussion

The findings of the HFO trial showed that, with respect to seizure outcome at 1 year, HFOs were not non-inferior to spikes on intraoperative electrocorticography for tailoring of epilepsy surgery, both overall and by epilepsy type (ie, temporal lobe epilepsy and extratemporal lobe epilepsy). Non-inferiority was not shown because the lower bound of the 90% CI for the risk difference was lower than –10%. Potential superiority of spikes was suggested for the whole group and for the subgroup with temporal lobe epilepsy, but not for the subgroup with extratemporal lobe epilepsy, because the upper bound of the 90% CI was lower than 0%. However, this possibility will need testing in a further study. Confounder analyses showed that poor prognosis pathology affected seizure outcome, and correcting for this confounder yielded inconclusive results for the whole group and the subgroup with temporal lobe epilepsy, but indicated non-inferiority for HFO-guidance in the subgroup with extratemporal lobe epilepsy. Secondary outcome measures (surgical duration, resection volume, cognition, quality of life before and after surgery, neurological deficits at discharge, and serious adverse events) did not differ between HFO and spike guidance. Intraoperative electrocorticography recording time and pre-existing neurological deficits were associated with poor seizure outcome, and a relation was found between seizure outcome and postoperative quality of life.

We hypothesised that we would show non-inferiority of HFO guidance for the entire study population based on results of retrospective studies. A meta-analysis of 11 retrospective studies, looking at the relation between resection of HFO-generating areas on invasive EEG and surgical outcome, showed a difference in HFO resection ratios over resected and non-resected tissue between seizure-free and recurrence outcome groups.^18^ The effect sizes were small but in favour of HFOs. We reported a risk difference that was opposite to our assumed success rate of 65% for spikes and 80% for HFOs. The 90% success rate in the spike group exceeded previous success rates for epilepsy surgery with intraoperative electrocorticography. Participation in a trial might account for this high success rate, and several factors might account for the negative HFO group findings. First, there could be a publication bias of retrospective results merely showing group level statistics. Second, the association between removal of HFO-generating areas and seizure outcome in retrospective studies might not reflect a causal link and, thus, might not necessarily translate into improved outcome after HFO-guided surgery. Third, HFOs are difficult to record and interpret in intraoperative electrocorticography, whereas spikes are less troubled by operating theatre noise and sampling errors. Fourth, the effect of intraoperative electrocorticography—and specifically of the individual events (ie, HFOs or spikes)—on clinical decisions was lower than anticipated. Fifth, clinically relevant variables were unequally distributed over the groups, which is a risk in a randomised trial in a heterogeneous population. Finally, the negative effects found in the whole group could mainly be contributed by temporal lobe epilepsy, because physiological mesiotemporal HFOs affect the interpretation of pathological HFOs. We excluded people with occipital lobe epilepsies and were cautious around functionally eloquent areas for the same reason.

Recording of neither HFOs nor spikes over the hippocampus has been shown to be predictive of postsurgical outcome in individuals undergoing temporal lobe lesionectomies without hippocampectomies.^10^ Unpublished data from our group (in people not participating in the HFO trial) have lent support to this finding. Epileptic HFOs mainly arise from principal neuron action potentials.^19^, ^20^ Synchronisation of neural populations leads to episodes of high-frequency population spikes, extracellularly reflected as HFOs. Proposed mechanisms for fast synchronisation are gap junctions and ephaptic interactions.^21^ Purely neuronal HFOs can occur, usually ripples, but out-of-phase firing of groups of neurons can lead to fast ripples.^20^ The healthy cortex does not produce fast ripples (except within the eloquent cortex, including the hippocampus), which potentially accounts for why the hippocampal HFOs are less predictive of the epileptogenicity of a single hippocampus. HFOs might be used to determine the extent of the resection of extramesiotemporal lesions, as has been shown in other studies in predominantly extratemporal lobe epilepsy with lesions.^15^, ^22^, ^23^, ^24^, ^25^ We did not predefine strict cut-off values for the number of HFOs in our study protocol, but on the basis of our previous research and literature, we put more emphasis on fast ripples than ripples, and we used cut-off values that would come down to more than one fast ripple per min (80–250 Hz) and more than five ripples per min (250–500 Hz).^13^, ^14^

A strength of our study is that it is the first of its kind in this field. Randomised trials of epilepsy surgery have shown surgery to be superior to long-term antiseizure medication for temporal lobe epilepsy in adults, and for all types of epilepsy in children.^26^, ^27^ We designed our study to show non-inferiority of intraoperative electrocorticography-tailoring based on HFOs before considering a superiority trial, because a superiority trial requires a large sample size and multiple participating centres, together with development of standardised analysis and clinical decision strategies. Optimal intraoperative use of HFOs and related EEG signal biomarkers will require innovations in data recording, data analysis, and feedback of results to neurosurgeons. We first needed to show that HFOs were clinically relevant biomarkers so we could stimulate these innovative steps.

A limitation of our study is the skewed a-priori chances of success for people in the HFO and spike guidance groups. We aimed for stratification by epilepsy type (temporal lobe epilepsy and extratemporal lobe epilepsy) and participating centre. We anticipated that this strategy would equalise adults and children over both groups, but this expectation did not work out because the second of two adult study sites withdrew before any participants had been enrolled. Correcting all variables by stratification would have been impossible within our sample size, but excluding participants with an a-priori low chance of achieving seizure freedom—eg, people with multiple lesions or previous surgeries—could have partly prevented the skewness. Another limitation is that the study sample is representative of people undergoing intraoperative electrocorticography at UMC Utrecht, including many with lesions, but not necessarily for the whole epilepsy surgery population.

Some clinicians raised concerns at study onset about the ethics of basing surgical decisions on novel EEG biomarkers that could easily be confused with physiological activity or artefacts. We set up the study with the overall intention of treating people in the best manner, and we used HFOs—similar to spikes—to adjust rather than to plan the surgical strategy. That is, the resection was equally restricted to non-eloquent cortex, and cortical areas showing events remote from the planned resection site were not removed. The surgical plan was based on all presurgical diagnostics and was confirmed without adaption in 25 participants and adapted in 28 study participants. Specific ictiform spike patterns were considered in both groups,^3^ which we believe led to safe interpretation of HFO results. Another concern raised before the start of our study by clinicians was the effect of visual analysis of HFOs in the electrocorticogram on surgery duration. This secondary endpoint did not differ between the random assignment groups.

We encountered technical and practical issues during the study that affected the results, interpretation of other studies, and future study plans. The background noise in the operating theatre—from the surgical microscope, the heating blanket, the neuronavigational hardware, and the coagulator—affected intraoperative electrocorticography recordings. We started to turn off or turn aside all noise-generating devices temporarily. We found that the configuration of cables and EEG headbox type affected background noise in the fast ripple range and that using proper hardware is crucial. We aimed to record several minutes of an artefact-free signal without burst-suppression pattern. We stopped propofol for each recording until a continuous EEG background pattern was reached. At other centres, sevoflurane is reduced, or propofol is replaced with sevoflurane.^15^, ^25^

HFOs are not ready for clinical interpretation as we investigated in this trial, and particularly not in temporal lobe epilepsy. Future work should focus on distinguishing epileptic and physiological brain activity with advanced signal analysis and establishing physiological activity atlases. Technical innovations needed include high-density recordings, very high sampling frequency, and low intraoperative recorded noise.^28^, ^29^ Another challenge is intraoperative recording from depth. We need industrial developments to propel technical advances and allow optimal clinical data integration and display to enable real-time intraoperative surgical guidance. Research and innovation need to go hand in hand to achieve high success rates for epilepsy surgery. The results of the HFO trial provide directions for the setup of a multicentre superiority randomised trial, which might be possible after improved recording, sampling, and interpretation of HFOs.

## Data sharing

We support data sharing within the restrictions of the ethical approval permissions. Requests can be made to the corresponding author.

## Declaration of interests

We declare no competing interests.
