# Supplementary material for: Intraoperative electrocorticography using high-frequency oscillations or spikes to tailor epilepsy surgery in the Netherlands (the HFO trial): a randomised, single-blind, adaptive non-inferiority trial
Source: Lancet Neurol. 2022 Nov;21(11):982–93. doi: 10.1016/S1474-4422(22)00311-8 (PMC9579052; doi:10.1016/S1474-4422(22)00311-8)
Supplement: Supplementary appendix [file mmc1.pdf]

THE LANCET
Neurology

Supplementary appendix

This appendix formed part of the original submission and has been peer reviewed. We post it as supplied by the authors.

Supplement to: Zweiphenning W, van 't Klooster MA, van Klink NEC, et al. Intraoperative electrocorticography using high-frequency oscillations or spikes to tailor epilepsy surgery in the Netherlands (the HFO trial): a randomised, single-blind, adaptive non-inferiority trial. *Lancet Neurol* 2022; **21:** 982–93.

SUPPLEMENTARY MATERIAL

Intraoperative electrocorticography using high-frequency oscillations or spikes to tailor epilepsy surgery (the HFO trial): a randomized controlled, single-blinded, non-inferiority trial in the Netherlands

Table of contents

1 Study oversight 3
1.1 Schematic HFO trial protocol 3
1.2 Protocol amendments 4
2 Neuropsychological evaluation (secondary outcome variable) 5
3 The HFO trial protocol (incl. statistical analysis plan) 6
3.1 List persons involved 11
3.2 Summary 16
3.3 Introduction and rationale 18
3.4 Objectives 22
3.5 Study design 23
3.6 Study population 24
3.7 Treatment of subjects 27
3.8 Methods 28
3.9 Safety reporting 34
3.10 Statistical analysis 37
3.11 Ethical considerations 38
3.12 Administrative aspects and publication 40
3.13 References 42
4 Interim analysis IDMC 44
4.1 IDMC report: 20 patients included and FU of 6-8 weeks (dd 17-04-2016) 44
4.2 IDMC report: 40 patients included and FU of 6-8 weeks (dd 07-04-2017) 47
4.3 Efficiency & safety analysis interim analysis 40 patients 52
4.4 IDMC charter 53

5 Characteristics non-participants 60

6 Additional analyses 61

6.1 Baseline characteristics 61

6.2 Uni- & multivariable logistic regression analysis 64

6.3 Intraclass Correlation Neurosurgeons 65

6.4 Per protocol analyses 66

6.5 Per-patient ECoG information 69

1 Study oversight

The protocol of the HFO trial has been previous published in trials[1]: doi: 10.1186/s13063-015-0932-6
The original protocol can be found in chapter 6.

1.1 Schematic HFO trial protocol

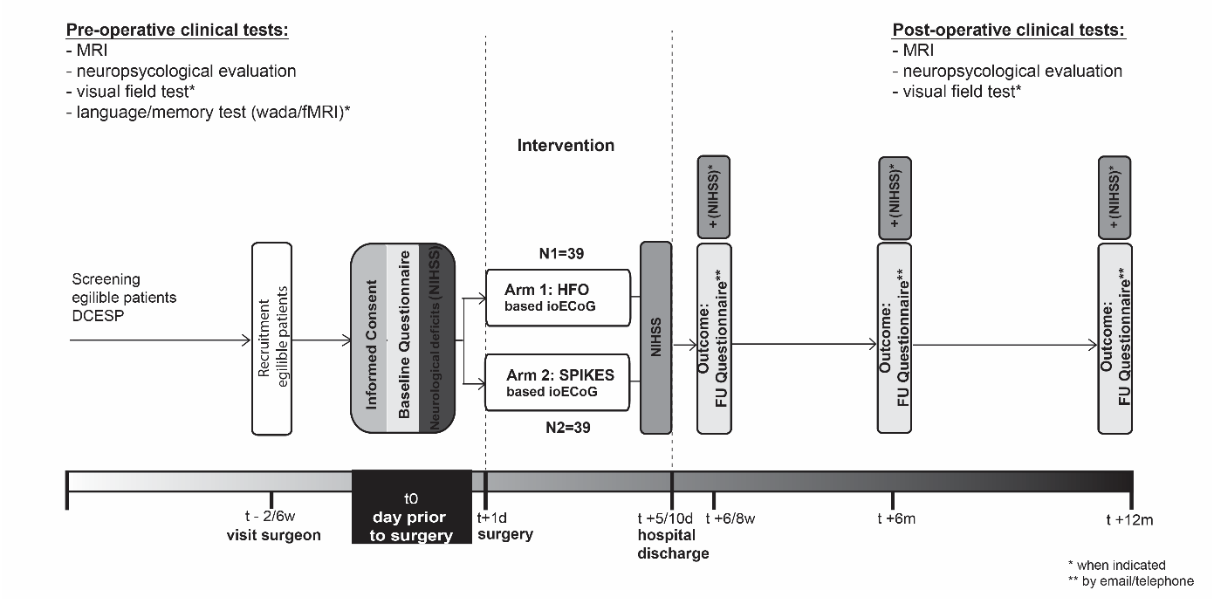

Figure S1: Trial protocol. A schematic representation of the study protocol and interventions. After the patients signed informed consent, a baseline questionnaire and neurological examination (using the National Institutes of Health Stroke Scale (NIHSS)) were completed. Via the follow-up questionnaire information about (preliminary) seizure outcome, anti-epileptic drug use, quality of life (QoL) and occurrence of serious adverse events (SAEs) was collected. Additional information was collected from route clinical test that was performed during the pre- and postsurgical period. (Adjusted from Van 't Klooster et al. 2015).[1]

[1] van 't Klooster MA, Leijten FSS, Huiskamp G, et al. High frequency oscillations in the intra-operative ECoG to guide epilepsy surgery ('The HFO Trial'): Study protocol for a randomized controlled trial. Trials 2015; 16. DOI:10.1186/s13063-015-0932-6.

1.2 Protocol amendments

Amend-ment	Initiator	Major changes	Date approval MEC
#1	PI	- Implemented a digital CRF & online questionnaires<br>- Included AE follow-up in questionnaires<br>- Updated IC; including permission to request data from other practitioners	Aug 2014
#2	PI	- Extend SAE monitoring during 1y FU<br>- Ask IC on day of hospital admission	Oct 2014
#3	PI	- Changes in data collection:<br>▪ NPO data: collect VAS data only, cancel Quoli89 data<br>▪ No collection of blood samples<br>- Update IC procedure and local investigator of 2nd site	May 2015
#4	PI	- Adapted in- & exclusion criteria:<br>▪ inclusion critria 'refractory epilepsy' removed<br>▪ exclusion of 'tumor patients' added<br>- Cancelled Aes reporting during 1y FU.	July 2015
#5	PI	- Closed 2nd study site (Vumc). No patients have been included<br>-Changed minimal time between first information and signing IC to 1 week.	Oct 2016

Table S1: Summary major changes HFO trial protocol amendments. Note: details regarding the changes made per amendment can be found in the section "History changes protocol" on page 2 of the protocol.

2 Neuropsychological evaluation (secondary outcome variable)

Pre- & 12 month post-operative neuropsychological evaluation (NPE) was performed as part of the clinical routine exams. Dutch versions of standardized tests corresponding with the patient's age and cognitive level were chosen and tested by a clinical neuropsychologist. Different versions of these tests all report on the corresponding domains of cognition. We assessed changes in cognitive functioning divided in four domains: overall cognitive function (OCF), working memory (WM), processing speed (PS), and memory consolidation (MC). Overall cognitive function (OCF) was calculated as a composite score (Table S1). The difference scores were normalized by the transformation to z-scores (separately for children and adults to obtain population-normalized scores) to represent common metric measurements. Subsequently, the individual patients' results per domain were dichotomized into negative (Δ z-score: -4 - <0.5), no (Δ z-score: >=0.5 - <=0.5), or positive (Δ z-score: >0.5 - 4) change. Data analysis was performed on complete case basis per domain, since not all participants underwent both pre- and post- NPE and/or the same test.

Domains	Calculated as	Variable explanation
**Overall Cognitive Function (OCF)**	$\Delta OCF = \text{mean}(\Delta\text{TotalIQ} + \Delta\text{WM} + \Delta\text{PS} + \Delta\text{MC})$	Intelligent tests used to determine ΔTotalIQ:<br>▪ <3.5y - Bailey Scales of Infant Development (BSID, versions II, III, IV; NL) For children in whom only a mental development age was reported we re-calculated the total IQ as follows: TotalIQ = mental dev age /chronological age *100.<br>▪ 2.5-6y - Weschler Preschool and Primary Scale of Intelligence (WPPSI, versions III, III; NL)<br>▪ 6-15 y - were tested by the Wechsler Intelligence Scale for Children (WISC, versions II, III, III, V; NL)<br>▪ 16+ y - Wechsler Adult Intelligence Scale (WAIS, versions II, III, III, V; NL)
**Working Memory (WM)**	For adults:<br>$\Delta WM = \Delta DigitSpan_{bw}$<br><br>For children:<br>$\Delta WM = \Delta DigitSpan_{norm}$	Adults working memory scores were based on the Digit span backward scores ($\Delta DigitSpan_{bw}$) for part of the WAIS intelligence scale. Children's working memory scores were based on the Digit span norm score ($\Delta DigitSpan_{norm}$) WISC or WAIS intelligence scale, which includes both digit span backward and forward to obtain reliable scores on this domain for this age group.
**Processing Speed (PS)**	For adults (18+y):<br>$\Delta PS = mean\ (\Delta PS_{raw} + \Delta TMT_{raw} + \Delta Stroop_{raw})$<br><br>For children (<18y):<br>$\Delta PS = mean\ (\Delta PS_{index})$	Processing speed for adults was assessed by a composite of the raw scores from three tests;<br>▪ the Processing Speed from the intelligence scale (WAIS) (ΔPS_{raw})<br>▪ the Trial Making Test (TMT, card A) (ΔTMT_{raw}) ,<br>▪ the Color Word Interference Test (Stroop test; mean card 1 and 2) ($\Delta Stroop_{raw}$)<br>For children this accessed through the possessing speed index score of the intelligence scale (ΔPS_{index}) was used, which is normalized to children's age.
**Memory Consolidation (MC)**	$\Delta MC = \Delta 15WT_{postponed}$	Memory consolidation was evaluated based on the postponed raw score of the 15-Word Task test for both adults and children.

Table S3: Details assessment of change in cognitive functions. Change in cognitive function (divided into four domains: overall cognitive functioning (OCF), working memory (WM), processing speed (PS) and memory consolidation (MS) is defined as one-year post- compared to pre-surgical neurophysiological assessment (Δ score).

3 The HFO trial protocol (incl. statistical analysis plan)

RESEARCH PROTOCOL

Intra-operative detection and localisation of high frequency oscillations in the ECoG to guide epilepsy surgery

Short title: "The HFO-study"

Protocol number METC: 13-389
ABR dossier number: NL44527.041.13 (**ABR number:** 44527)
Dutch Epilepsy Foundation number: 2012-4
ClinicalTrials.gov Identifier (NCTnr): NCT02207673

HISTORY CHANGES PROTOCOL

In the table below all changes made to protocol are described per version of the protocol.

**Protocol version**	**Type**	**Description changes**
**Version 8 (nr 1): Aug 2016**	Amendment Nr 5.	- As of 19-7-2016 the VUmc has been officially closed as a study site. No patients have been included there. Information about VUmc removed from protocol. Changed: B1_ABR_formulier, C1_onderzoeksprotocol, E1_E2_Patientinformatiebrief&Toestemmingsformulier_UMCU, I1_Lijst deelnemende centra, K6_Monitoringplan_HFOs, K6_Brief_huisarts_of_behandelend_neuroloog.<br>- As of 1-12-2015 new study coordinator: W.J.E.M. Zweiphenning. Changed: B1_ABR_formulier, C1_onderzoeksprotocol, E1_E2_Patientinformatiebrief&Toestemmingsformulier_UMCU, F1_Uitnodiging&herinnering online vragenlijst, F1_Vragenlijst_Baseline&FU_aanvallen_ae&qol, H2_CV_coordinerend_onderzoeker, K6_Brief_huisarts_of_behandelend_neuroloog.<br>- Minimal time period between providing information about the study and signing informed consent is changed to 1 week. Changed: C1_onderzoeksprotocol, E1_E2_Patientinformatiebrief&Toestemmingsformulier_UMCU<br>- Study procedure added to C1_onderzoeksprotocol: duration surgery is defined as ‘start incision’ to ‘end surgeon’.
**Version 7 (nr 1): Jun 2015**	Amendment Nr 4.	-Par 6.2 Inclusion criteria: adaptation definition ‘refractory epilepsy’.<br>-Par 6.3 Exclusion criteria: adding tumor patients.<br>-Par 8.2 Randomisation and par 11.2 Recruitment and consent procedure: are the same in UMCU and VUmc.<br>- 9.2.1 Adverse events (AEs): no reportage of AEs during post-operative follow-up period.<br>-Par 10.1 Primary and secondary outcome; adaptations in statistical analysis as a consequence of revisions protocol publication for Trails.<br><br>**NB: note that version number of all PIFs&IC changed due flaw in attachment 2, however there are no changes made in the information content.**
**Version 6 (nr 1): Apr 2015** (->ABR version 5)	Amendment Nr 3.	-Page 6-7: New local investigator VUmc<br>-Par 8.1.2 Secondary study: Changes in NPO data available (Quoli89 deleted, only VAS) and data collection (via neuropsychologists)<br>8.3.1Pre-intervention procedures: no collection of blood samples<br>-Par 8.3.3 Post-interventional procedures: the NIHSS directly post-OK is invalid.<br>-Par 8.2 Randomisation and par 11.2 Recruitment and consent: added IC procedure for VUmc.<br><br>**NB: note that version number of all PIFs&IC changed due to ABR change, however there are no changes made in the informationcontent.**
**Version 5 (nr 1): oct 2014** (->ABR version 4)	Amendment Nr 2.	- Add history of changes in protocol section.<br>- Par 9.2.2 Serious adverse events (SAEs):<br>The period of SAE monitoring is changed to complete follow-up period, instead of only during post-operative hospitalization period. Included is the definition of normal post-OR hospitalisation period.<br>- Par 12.1 Handling and storage of data and documents: The specific name of “Eltje Bloemen”, as the research nurse that will keep the randomisation files is removed.

		- Par 11.2 Informed consent procedure on day of hospital admission prior to surgery (+ adaptation fig 2)
**Version 4 (nr 1): july 2014** (->ABR version 3)	Amendment Nr 1.	- Online follow-up questionnaire at 6-8 weeks instead of paper version.<br>- AE question added to follow-up questionnaire(s)<br>- eCRF in OpenClinica<br>- Clinicalgov trial number
**Version 3 (nr 1); dec 2013** (->ABR version 2)	Reply METC Nr 2.	- Add rationale pilot study<br>- Emphasize non-inferiority hypothesis 2<br>- Clarification sample size calculation 2
**Version 2 (nr 2): sept 2013** (->ABR version 2)	Reply METC Nr 1.	- METC number<br>- Emphasize non-inferiority hypothesis<br>- Inclusion table 1: epilepsy surgery in the Netherlands<br>- Explanation role LWEC and screening<br>- Clarification sample size calculation<br>- Explanation no stratification for child/adult<br>- Supplement on statistical analysis (primary & secondary outcome and interim analysis (safety and efficiency) by DSMB)

PROTOCOL TITLE: Intra-operative detection and localisation of high frequency oscillations in the ECoG to guide epilepsy surgery

**Short title**	**"The HFO study"**
**Version**	**Version 8 (nr 1)**
**Date**	**August, 2016**
**Coordinating investigator/project leader**	***W.J.E.M. Zweiphenning, MD,*** PhD Student<br>University Medical Center Utrecht<br>Department of Neurology and Neurosurgery,<br>Clinical Neurophysiology, hp: C03.131<br>Heidelberglaan 100, 3584CX Utrecht<br>Postbus 85500, 3508 GA Utrecht,
**Principal investigator(s) (in Dutch: hoofdonderzoeker/uitvoerder)**	***G.J.M. Zijlmans, MD, PhD***<br>Neurologist /Assistant Professor University Medical Center Utrecht<br>Department of Neurology and Neurosurgery, hp. G.03.228<br>Heidelberglaan 100, 3584CX Utrecht<br>Postbus 85500, 3508 GA Utrecht
**Sponsor (in Dutch: verrichter/opdrachtgever)**	**University Medical Center Utrecht**
**Subsidising party**	The work of W.J.E.M. Zweiphenning is funded by the UMC Utrecht Alexandre Suerman Stipendium<br>The work of G.J.M. Zijlmans is funded by the Rudolf Magnus Institute Young Talent Award
**Independent physician(s)**	**University Medical Center, Utrecht, The Netherlands:**<br>***B.H. van der Worp, MD, PhD***<br>Neurologist<br>Department of Neurology and Neurosurgery, hp. G.03.232<br>Heidelberglaan 100, 3584CX Utrecht<br>Postbus 85500, 3508 GA Utrecht
**Laboratory sites <*if applicable*>**	***n.v.t***
**Pharmacy <*if applicable*>**	***n.v.t.***

PROTOCOL SIGNATURE SHEET

Name	Signature	Date
**Head of Department:**<br>***Prof. G.J.E. Rinkel***<br>Head of department of Neurology & Neurosurgery<br>UMC Utrecht<br>G.J.E.Rinkel@umcutrecht.nl		
**Principal Investigator:**<br>***G.J.M. Zijlmans (MD, PhD)***<br>Neurologist/Assistant Professor<br>UMC Utrecht<br><br>**Coordinating Investigator:**<br>***W.J.E.M. Zweiphenning (MD)***<br>PhD student<br>UMC Utrecht		

3.1 List persons involved

3.1.1 Persons directly involved (first-line)

University Medical Center Utrecht, The Netherlands

- **F.S.S. Leijten**
  Neurologist/Clinical Neurophysiologist
- **C.H. Ferrier**
  Neurologist/Clinical Neurophysiologist
- **K.P.J. Braun**
  Head of Dept. Paediatric Neurology
- **P.C. van Rijen**
  Neurosurgeon
- **P.H. Gosselaar**
  Neurosurgeon
- **T.A. Gebbink**
  Physician Assistant Epilepsy Surgery
  G.J.M. Huiskamp
  Physicist Clinical Neurophysiology
- **M.J.C. Eijkemans**
  Biostatistician Julius Center

Other

- **P.R. Bauer**
  PhD student SEIN Heemstede
- **R. Zellmann**
  Post Doc Montreal Neurological Institute, McGill University, Canada

3.1.2 Persons involved (second-line)

University Medical Center Utrecht, The Netherlands

- **F.E. Jansen**
  Paediatric Neurologist
- **P.A.J. Robe**
  Neurosurgeon
- **N.R.V. Henriquez**
  Person in charge EEG technicians
- **O.B. Braams**
  Paediatric Psychologist
- **M.M.J. Schoneveld**
  Paediatric Psychologist
- **M.J.E. van Zandvoort**
  Clinical Neuropsychologist
- **J.M. Ophorst-vanEck**
  Nurse Practitioner Neurosurgery
- **G.A.P. de Kort**
  (Neuro)Radiologist
- **T.D. Witkamp**
  (Neuro)Radiologist
- **E.M. Bloemen-Carlier**
  Research nurse paediatric neurology
- **S.A.M. Goemans**
  Research Assistant

Other

- **W. Alphers**
  Clinical Neuropsychologist SEIN Heemstede
- **M.P.H. Hendriks**
  Clinical Neuropsychologist Kempenhaege
- **D. Velis**
  Neurologist

- **S. Claus**
  Neurologist
- **G. Visser**
  Neurologist/Head of department of clinical neurophysiology SEIN
- **J. Gotman**
  Professor at Montreal Neurological Institute, McGill University, Canada
- **The Dutch Collaborative Epilepsy Surgery Program ("Landelijke Werkgroep Epilepsy Chirurgie" (LWEC))**

TABLE OF CONTENTS PROTOCOL

2.1. List of persons involved 11
2.2. Summary 16
2.3. Introduction and rationale 22
2.3.1 Introduction 22
2.3.2 Rationale for a prospective study 20
2.4. Objectives 22
2.4.1 Primary Objective 22
2.4.2 Secondary Objectives 22
2.5. Study design 23
2.6. Study population 24
2.6.1 Population 24
2.6.2 Inclusion criteria 25
2.6.3 Exclusion criteria 25
2.6.4 Sample size calculation 26
2.7. Treatment of subjects 27
2.7.1 Investigational treatment 27
2.7.2 Detection of HFOs 27
2.7.3 The dedicated team 27
2.7.4 Use of co-intervention and escape medication 27
2.8. Methods 28
2.8.1 Study parameters/endpoints 28
2.8.1.1 Main study parameters/endpoints 28
2.8.1.2 Secondary study parameters/endpoints 28
2.8.2 Randomisation, blinding and treatment allocation 29
2.8.3 Study procedures 30
2.8.3.1 Pre-intervention procedures 30
2.8.3.2 Intervention 30
2.8.3.3 Post-interventional procedures 31
2.8.3.4 Withdrawal of individual subjects 32

2.8.3.5 Replacement of individual subjects after withdrawal 32
2.8.3.6 Follow-up of subjects withdrawn from treatment 32
2.8.3.7 Premature termination of the study 33
2.9. Safetly reporting 34
2.9.1 Section 10 WMO event 34
2.9.2 Adverse and serious adverse events 34
2.9.2.1 Adverse events (AEs) 34
2.9.2.2 Serious adverse events (SAEs) 35
2.9.3 Follow-up of adverse events 35
2.9.4 Data Safety Monitoring Board (DSMB) 35
2.9.5 Monitoring 35
2.9.6 Stopping rules 36
2.10. Statistical analysis 37
2.10.1 Primary outcome and Secondary outcome 37
2.10.2 Interim analysis 37
2.11. Ethical considerations 38
2.11.1 Regulation statement 38
2.11.2 Recruitment and consent 38
2.11.3 Objection by minors 38
2.11.4 Benefits and group relatedness 38
2.11.5 Compensation for injury 39
2.12. Administrative aspects and publication 40
2.12.1 Handling and storage of data and documents 40
2.12.2 Amendments 40
2.12.3 Annual progress report 40
2.12.4 End of study report 40
2.12.5 Public disclosure and publication policy 41
2.13. References 42

LIST OF ABBREVIATIONS AND RELEVANT DEFINITIONS

**ABR**	ABR form, General Assessment and Registration form, is the application form that is required for submission to the accredited Ethics Committee (In Dutch, ABR = Algemene Beoordeling en Registratie)
**AE**	Adverse Event
**aECoG**	acute Electrocorticogram
**AED**	Anti-epileptic drug
**AR**	Adverse Reaction
**CA**	Competent Authority
**CCMO**	Central Committee on Research Involving Human Subjects; in Dutch: Centrale Commissie Mensgebonden Onderzoek
**CV**	Curriculum Vitae
**DSMB**	Data Safety Monitoring Board
**EU**	European Union
**EudraCT**	European drug regulatory affairs Clinical Trials
**FRs**	Fast Ripples (Subclass of HFOs with activity in the ECoG in range of >250-500Hz)
**GCP**	Good Clinical Practice
**Group 1**	Randomized for HFO-based resection (new method).
**Group 2**	Randomized for epileptiform spike-based resection (current standard method).
**HFOs**	High Frequency Oscillations (Activity in the ECoG in range of >80-500Hz, new biomarker strongly associated with epileptogenic tissue)
**HRQOL**	Health related Quality of Life
**IB**	Investigator's Brochure
**IC**	Informed Consent
**(i)EEG**	(intracranial) Electroencephalogram
**IEMU**	Intensive Epilepsy Monitoring Unit
**LWEC**	National Collaborative Dutch Epilepsy Surgery Program (In Dutch: Landelijke Werkgroep Epilepsy Chirurgie. The LWEC is assembled of the (paediatric) neurologist, clinical neurophysiologists, neurosurgeons and neuropsychiatrists from the 3 Dutch hospitals performing epilepsy surgery (Universitary Medical Center Utrecht, VU University Medical Center Amsterdam and Academic Hospital Maastricht) and the 2 Dutch epilepsy centers (SEIN en Epilepsiecentrum Kempenheaghe).
**MEG**	Magneto-Encephalography
**METC**	Medical research ethics committee (MREC)(In Dutch: Medisch Ethische Toetsing Commissie
**(f)MRI**	(functional) Magnetic Resonance Imaging
**NP**	Nurse Practitioner
**NPO**	Neuropsychological test
**PA**	Physician Assistant
**PET**	Positron Emission Tomography
**Rs**	Ripples (Subclass of HFOs with activity in the ECoG in range of >80-250Hz)
**SAE**	Serious Adverse Event
**SPECT**	Single-photon emission computed tomography
**Sponsor**	The sponsor is the party that commissions the organisation or performance of the research, for example a pharmaceutical company, academic hospital, scientific organisation or investigator. A party that provides funding for a study but does not commission it is not regarded as the sponsor, but referred to as a subsidising party.
**SUSAR**	Suspected Unexpected Serious Adverse Reaction
**UMCU**	University Medical Center Utrecht
**VAS scale**	Visual analogue scale
**VUmc**	VU University Medical Center
**WBP**	Personal Data Protection Act (In Dutch: Wet Bescherming Persoonsgevens)
**WMO**	Medical Research Involving Human Subjects Act (in Dutch: Wet Medisch-wetenschappelijk Onderzoek met Mensen.

3.2 Summary

Rationale: Epilepsy occurs in 0.5-0.7% of the population, of which 25% are children. About 30% of patients with focal epilepsy do not respond well to medication and half of them are eligible for epilepsy surgery. In recent years, the importance of early epilepsy surgery has been stressed, because successful resection may lead seizure freedom, medication freedom, and improved social and cognitive development, especially in children. The surgical success rate depends on the localization of the source of the epilepsy: the epileptogenic tissue. Despite all efforts using MRI, EEG and other techniques, the site and extent of the epileptogenic tissue is never a 100% clear. The current success rate of epilepsy surgery is around 65%. During surgery intracranial electrocorticography (acute ECoG, aECoG) is recorded in some medical centers, and the presence of epileptiform spikes as identified by a clinical neurophysiologist is used to guide the neurosurgeon in deciding the extent of the cortical resection. These spikes are considered interictal markers of the presence of epilepsy. EEG spikes have a local distribution that exceeds or propagates outside the limits of the ictal onset zone and thus the probable epileptogenic zone. High Frequency Oscillations (HFOs, >80-500Hz) in the ECoG have recently been identified as a new biomarker for epileptogenic tissue. Retrospective research shows that their local presence strongly relates to the seizure onset zone, and removal of tissue with HFOs could predict a better surgical outcome, even better than seizures themselves. The area showing HFOs usually overlaps with, but is smaller than the area showing spikes, and HFOs do not tend to propagate to distant sites as spikes do. The identification of HFOs is more objective than of spikes and automatic detection software exists. HFOs currently require experience and time from observers, but could yield faster and better epilepsy surgery through the development of a new surgical device that gives onsite information to the surgeon. We want to study the use of intra-operatively recorded HFOs, and compare them to the currently used epilepiform spikes, in the decision about the extent of resection. In a first step to verify if development of this device is worthwhile and enables clinical use of HFOs we want to perform a pilot study to test the following hypothesis: *The intra-operative use of HFOs to delineate the epileptogenic cortex does not yield significantly worse outcome in seizure freedom than the current method based on spikes. Second we expect that less brain tissue will be removed with HFOs compared to spikes.*

Objective: Primary objective is to investigate whether delineation of the epileptogenic tissue during aECoG-guided surgery using HFOs instead of epileptiform spikes, will lead to a not significantly worse outcome with respect to post-operative seizure freedom (non-inferiority design). Secondary objectives are the volume of tissue resected, cognitive functioning, neurologic deficits, surgical complications and duration, and health related quality of life (pre- vs. post-operative change).

Study design: Single blinded randomized controlled trial.

Study population: Patients of all ages with epilepsy undergoing epilepsy surgery with aECoG to guide the extent of the cortical resection. At study completion 78 patients are included in the study.

Intervention: Eligible patients will be randomised, after informed consent is signed, into group 1 (HFOs) in whom resection of epileptogenic tissue is guided by HFOs in the aECoG (new), or into group 2 (spikes) in whom resection of epileptogenic tissue is guided by epileptiform spikes in the aECoG (current standard). Independent of the randomisation ictiform spike patterns will always be resected.

Main study parameters/endpoints: Outcome after epilepsy surgery after 1 year of follow-up dichotomized in total seizure freedom (Engel classification Ia&b) vs. seizure recurrence (Engel classification Ic-IV).

Nature and extent of the burden and risks associated with participation, benefit and group relatedness:
The burden for a participant in this study is considered low. The participant or the legal representatives have to answer a short online/telephone questionnaire (7 questions) on seizure occurrence, adverse

events and quality of life at 6-8 weeks, 6 and 12 months. At baseline (pre-surgery) this short questionnaire will also be filled in during the patient outward hospital visits. All other data will be collected from the routine pre- and post-epilepsy surgery trajectory.

The risk associated with participation is that using HFOs could lead to a smaller resection, which could potentially lead to seizure recurrence, and in a worst case scenario a second surgery is necessary. The benefits associated with participation are that the new biomarker, HFOs, are expected to perform better than spikes, therefore resulting in equal or better surgical outcome. Potential benefits from a smaller or more precise resection would be reduced neurological deficits, and a better quality of life.

Children with intractable epilepsy pose a diagnostic and therapeutic challenge and nowadays are considered for early epilepsy surgery, because successful resection of epilepsy generating foci may lead to seizure- and medication freedom, and may improve social, psychological and cognitive development. A shift in epilepsy surgery towards this paediatric population has set over the last years and the proportion of children is still increasing. The study population consists of mostly children (>40%) because the main research question about post-surgical outcome and the expected effect of reducing neurological and cognitive deficits are most relevant in this population.

Because this study will also include children below the age of 12, the NFU risks category is graded as a “moderate risk” study. Monitoring is scheduled with high incidence during the first year of inclusion. Further an independent external DSMB is installed that primarily evaluates the safety of the study and performs (early) interim analyses. To enable interim analysis after inclusion of the first 20 and 40 patients, preliminary post-surgical outcome is determined at 6-8 weeks and 6 months, additional to the final outcome at 12 months.

By participating in this study, intractable epilepsy patients will contribute to improvement of the current knowledge of localization of epilepsy generating tissue as well as improvement of epilepsy surgery outcome.

3.3 Introduction and rationale

3.3.1 Introduction

Epilepsy occurs in 0.5-0.7% of the population (Engel 1993). The estimated number of new cases in European children and adolescents is 130 000/year (incidence rate 70 per 100 000). In adults (20-64 years) this number is 96 000 cases per year (incidence rate 30 per 100 000) and 85 000 in the elderly of 65 years and older (incidence 100 per 100 000) (Forsgren, Beghi et al. 2005). The highest incidence of epilepsy is in the paediatric and adolescent population. About 30% of patients with focal epilepsy don't respond well to medication (Sander 2003), and 50% of these patients are eligible for surgery (Rosenow and Luders 2001). The surgical success rate depends on exact localization of the source of epilepsy: the epileptogenic tissue. Despite all effort with MRI, EEG and other pre-operative techniques, the site and extent of the epileptogenic tissue is never a 100% sure.

Pre-operative localization of the epileptogenic tissue and eloquent cortex is costly and time-consuming. It includes multiple specialists' consultations, recording seizures with long-term (usually 1 week) video-EEG, MRI, (EEG-)fMRI, magneto-encephalography (MEG), PET and ictal SPECT scans. If doubt remains, in a select group of difficult patients epilepsy surgery is preceded by long-term intracranial EEG: either subdural electrode grids are implanted intracranially or needle electrodes are inserted through the scalp and skull. Usually this registration takes a week, in which the patient is monitored for occurrence of (spontaneous) seizures. However, most patients do not undergo long-term intracranial EEG. When the target region is generally known from non-invasive studies, surgery follows. In some centers the exact delineation of the area of resection depends on a final step during surgery, using intra-operative or acute electrocorticography (aECoG). The clinical neurophysiologist then evaluates the aECoG for so-called epileptiform spikes (in the frequency range <70Hz) that indicate epileptic involvement of underlying cortex. This guides the neurosurgeon in the final decision on the extent of the cortical resection. The primary reason for aECoG registration is tailoring of the resection, with/without functional mapping. A second reason for aECoG registration is prognosis determination; presence of residual spikes post-resection is correlated with diminished change on seizure freedom (Alarcon, Garcia Seoane et al. 1997; Stefan, Hopfengartner et al. 2008).

This so-called 'tailoring' of the resection based on spikes in the aECoG is under international debate. The critique is that not all spikes may signify epilepsy and that spikes tend to spread locally, away from its origin, into non-epileptic surrounding areas. While epileptiform spikes are highly specific for epileptic brain, they are not a particularly good biomarker because they are only loosely related to disease activity. Also, reactive post resection spikes can arise on the resection margin (Schwartz, Bazil et al. 2000). This could lead to unnecessary removal of tissue (Alarcon, Garcia Seoane et al. 1997; Worrell and Gotman 2011).. The discussion is now focused on the differentiation of 'red' spikes, true indicators of the epileptogenic zone, versus 'green' spikes, secondary spikes. For this same reason there are medical centers that perform epilepsy surgery standard without aECoG. The only proven types of 'red' spikes are 'ictiform spike patterns', which are ictal or continuous epileptiform discharges that are strongly related to tumour and cortical dysplasia. These ictiform spike patterns consist of recruiting patterns, repetitive bursting patterns and especially continuous rhythmic spiking. They occur in approximately 50% of the patients with a tumour or focal cortical dysplasia undergoing aECoG (Palmini, Gambardella et al. 1995; Ferrier, Aronica et al. 2006). Complete resection of cortical tissue displaying these clearly defined patterns is positively correlated with post-surgical outcome (Palmini, Gambardella et al. 1995). Therefore these ictiform patterns are always included in the resection without discussion. However, besides these ictiform patterns still individual spikes with unknown relevance for removal are present.

A new biomarker of epileptogenic tissue has emerged, named High Frequency Oscillations (HFOs), in the frequency range of 80-500Hz, which can be subdivided into Ripples (R), in the range

of 80-250Hz, and Fast Ripples (FR), in the range of 250-500Hz. Formerly, these frequency ranges were filtered out of the recording. However, over the past decade interest in HFOs has grown substantially. It was discovered that HFOs are a more reliable marker of local cortical epileptogenic properties than their traditional counterparts, epileptogenic spikes. HFOs have been shown to occur in the seizure onset zone and co-occurring with spikes as well as independently (Jacobs, Levan et al. 2009). Retrospective studies showed that removal of tissue producing HFOs even predicted a better surgical outcome, compared to removal of the ictal onset zone as defined by intracranial EEG (Jacobs, Zijlmans et al. 2010; Wu, Sankar et al. 2010). The area showing HFOs is usually smaller than the area showing spikes (Jacobs, LeVan et al. 2008). Though these areas show partial overlap, some electrodes show HFOs without spikes and others show spikes without HFOs.

Apart from better localizing the epileptogenic tissue, the behaviour of HFOs seems a sign of the tissue's intrinsic epileptogenicity and therefore more intuitively linked to seizures than are spikes. In the kainaic acid epilepsy model in rats, HFOs precede the first occurring spontaneous seizures (Bragin, Wilson et al. 2004). HFOs increase when antiepileptic medication is reduced and HFOs show a further build-up directly before seizures into seizure onset (Zijlmans, Jacobs et al. 2009; Zijlmans, Jacobs et al. 2011). The number of HFOs to occur during surgery with aECoG increased with emergence from propofol (known for its anti-epileptic properties) anaesthesia, while the number of spikes remained unchanged (Zijlmans, Huiskamp et al. 2012). Therefore the behaviour of HFOs is markedly different from the behaviour of epileptiform spikes, which are not affected by medication and show a counter-intuitive decrease in number before seizures while they increase in numbers after seizures. HFOs appear to be a direct result of the underlying epileptogenic process leading to increased excitation and seizures, whereas traditional spikes seem more complex and related to inhibitory responses (Zijlmans, M., P. Jiruska, et al. (2012).

With respect to the major paediatric epilepsy population, Wu et al (2010) already demonstrated in a retrospective study that HFOs are present in intraoperative ECoG in 80% of the paediatric epilepsy cases (n=30) and that complete resection of tissue containing FR was strongly correlated with postoperative seizure freedom (Wu, Sankar et al. 2010). Several other retrospective studies in paediatric populations confirmed this finding, independent of the underlying cause of the epilepsy and for both ictal and interical HFOs (Ochi, Otsubo et al. 2007; Jacobs, Zijlmans et al. 2010; Wu, Sankar et al. 2010; Akiyama, McCoy et al. 2011; Nariai, Matsuzaki et al. 2011; Fujiwara, Greiner et al. 2012).

However, in some brain areas spontaneous physiological HFOs have been described, namely the mesiotemporal structures (Zijlmans, Jiruska et al. 2012) and the occipital lobe (Nagasawa, Juhasz et al. 2012). In the mesiotemporal region, these physiological HFOs can be distinguished from epileptic HFOs. Physiological HFOs occur at a frequency up to 250 Hz (ripples), whereas fast ripples (250-500 Hz) are always pathological in this area (Bragin, Engel et al. 1999a; Bragin, Engel et al. 1999b). Currently, it is not possible to discriminate between pathological or physiological occipital HFOs, strongly discouraging HFO guided resections in patients with a presumed occipital focus.

HFOs can be found in the aECoG, when it is recorded at a sample frequency above the usual 500 Hz and all frequencies below 80 Hz are removed (Zijlmans, Jiruska et al. 2012). Visual recognition of HFOs gets easier over time with increased experience, comparable to the learning process for identification of spikes. The interobserver agreement for HFOs is higher than for spikes (Zelmann, Zijlmans et al. 2009). Automatic detection methods have been developed for HFOs (Zelmann, Zijlmans et al. 2009; Zelmann, Mari et al. 2012) and more methods follow quickly, while the automatic detection of spikes is difficult and the distinction between spikes that are clinically relevant and other spikes requires visual evaluation and coverage of a larger brain area. The objective and automatic detection of HFOs could lead to the future development of a new surgical device that informs the surgeon continuously and leads to faster and better surgery. Such a device could even be

combined with a treatment device. Spikes are not suited for such a device and evaluating the ECoG for spikes requires experience and is subjective and requires recording with multiple electrodes. The method is under international debate and even not used in a great part of the epilepsy surgery centers.

3.3.2 Rationale for a prospective study

The success rate of epilepsy surgery is around 65%; in resections for temporal lobe epilepsy (TLE) 60-90% of patients achieve seizure freedom while in extra-temporal epilepsy it is around 40-65% (Wiebe, Blume et al. 2001; Tellez-Zenteno, Dhar et al. 2005). In recent years the importance of early epilepsy surgery has been stressed. Children with intractable epilepsy pose a diagnostic and therapeutic challenge and should be considered for early epilepsy surgery, because successful resection of epileptic foci may lead to better social, psychological and cognitive development (Kumar, Juhasz et al. 2010). Adult surgery is more and more considered as 'delayed child surgery' as in most patients refractory epilepsy usually evolves in childhood and early adolescence (Forsgren, Beghi et al. 2005; de Boer, Mula et al. 2008). The percentage of paediatric epilepsy surgery will increase in the coming years. The department of paediatric neurology of the UMCU *has developed high expertise in childhood epilepsy and epilepsy surgery. Therefore, children are an important target population.* Epilepsy surgery takes place in about 110 patients a year in the Netherlands, of which 55% (75% age <18 years) is performed at the UMC Utrecht, 27% at the VUmc and 18% at the UMC Maastricht. In these Dutch centers, aECoG is recorded during surgery. The number of patients at the UMC Utrecht has increased since a new collaboration was formed with the Stichting Epilepsie Instellingen Nederland in 2015. Decision making for resection during surgery is currently based on the occurrence of epileptiform spikes in this aECoG. The potential advantage of HFOs are: 1) more seizure freedom after surgery, 2) smaller resection with removal of less brain tissue, 3) the possibility to develop a quick, automatic and objective HFO detection device which needs only few electrodes. Spikes do not lend themselves for such a device.

Results from retrospective studies, as summarized above, suggest that for surgical decision making on the extent of the resection, HFOs in the aECoG may be better biomarkers than traditional spikes for surgical outcome and extent of resection. An international demand for convincing data of the use of HFOs during epilepsy surgery is rising (Engel and da Silva 2012; Jacobs, Staba et al. 2012; Zijlmans, Jiruska et al. 2012). To prove that HFOs yield better outcome in seizure freedom requires a large multicenter trial. Setting up a multicenter international trial is difficult. Few centers perform aECoG, and the ones that do, use different intra-operative recording techniques and software. Moreover, the experience with the interpretation of HFOs is limited to even less centers and currently requires time and expertise.

The aim of epilepsy surgery is seizure freedom and neurosurgeons are not motivated to try a new technique, like our proposed detection device, as long as it has not been proven that the new method is at least not doing worse for seizure freedom.

Therefore our aim is to prove, in the small setting of our Dutch center already performing aECoG during epilepsy surgery, that using HFOs for tailoring resection is as good as epileptogenic spikes, in achieving seizure freedom.

Only by performing this prospective study we can investigate the feasibility and safety of HFOs for improvement of epilepsy surgery and take the next step in the development of a new surgical detection device. This would open doors towards acceptance of the use of HFOs and participation in a big international multi-center trial that is needed to demonstrate superiority of HFOs. We want to compare the outcome of aECoG-guided surgery using HFOs as a biomarker for tailoring versus the traditional use of epileptiform spikes, with the additional remark that ictiform spike patterns are always accounted for, independent of the biomarker used for tailoring. We expect that the use of HFOs for delineation of the epileptogenic cortex will lead to an equal (or improved) outcome in

seizure freedom (primary objective), to removal of less brain tissue resulting in better neurologic and cognitive function and improved quality of life (Qol) (secondary objectives).

3.4 Objectives

3.4.1 Primary Objective

To investigate whether delineation of the epileptogenic tissue during aECoG-guided surgery guided by HFOs instead of epileptiform spikes, will not lead to a worse outcome with respect to post-operative seizure freedom.

3.4.2 Secondary Objectives

To see whether the HFO-based, compared to spike-based, aECoG interpretation will lead to differences in:

- volume of brain tissue removed
- neurological deficits
- cognitive functioning
- health related quality of life (HRQOL)
- duration of surgery (defined as: time incision ("start incisie") – time surgeon done ("eind operateur")) and aECoG recording
- surgical complications

3.5 Study design

A single blinded randomised clinical trial will be performed. If informed consent is given, participating epilepsy surgery candidates who will undergo surgery using aECoG will be randomized into HFO-guided (group 1) or epileptiform spike-guided (group 2) resection. Independent of the randomisation, ictiform spike patterns will always be resected (if allowed by the localization of eloquent functions). See paragraph **Fout! Verwijzingsbron niet gevonden.**.

A RCT-design with stratification for epilepsy type (temporal lobe epilepsy vs. extra-temporal lobe epilepsy) is used to avoid a priori differences in prognosis between the two populations.

3.6 Study population

3.6.1 Population

Epilepsy surgery takes place in about 110 patients per year in the Netherlands, of whom 60 patients (75% age <18 years) a year at the UMC Utrecht and 25-30 patients a year at the VUmc[2]. In all patients at the VUmc and in 30-35 patients at the UMC Utrecht, aECoG is recorded during surgery. The number of patients at the UMC Utrecht has increased since a new collaboration started between the UMC Utrecht and the Stichting Epilepsie Instellingen Nederland in 2015. *The study population will consist of all these patients, including children. These patients are referred to the neurosurgeon by the Dutch Collaborative Epilepsy Surgery Program ("Landelijke Werkgroep Epilepsie Chirurgie" (LWEC). The aim of surgery is curing the patient from epileptic seizures. See table 1.*

Table 1: Overview epilepsy surgery in the Netherlands until 2015.

	Patients/ Year	*Patiens/Year @ 80% participation*	*% Children (≤17 years)*	*% Adults (>17 years)*
Total epilepsy surgery in the Netherlands	*100*			
• *Total epilepsy surgery in UMCU*	*60*			
- ***Epilepsy surgery with aECoG***	***30 à 35****	***24 à 28 ->round off 25***	***≥75% (N=18)***	***≤25%(N=7)***
• *Total epilepsy surgery in VUmc*	*25 à 30*			
- ***Epilepsy surgery with aECoG***	***25 à 30***	***20 à 24 ->round off 20***	***-***	***100% (N=20)***
• *Total epilepsy surgery in MUMC*	*10*			

*Note that aECoG tailoring is used in a sub selection of all epilepsy surgeries (column 1&2). Inclusion rate is based on an 80% participation (column 5). Note that pediatric surgery is only performed in the UMCU (column 3 & 4). * The number of patients at the UMC Utrecht has increased since a new collaboration started between the UMC Utrecht and the Stichting Epilepsie Instellingen Nederland in 2015.*

3.6.2 Inclusion criteria

The "Landelijke Werkgroep Epilepsie Chirurgie" (LWEC) discusses potential candidates for epilepsy surgery during their monthly meetings. The LWEC decides in this meeting for which type of surgery is opted; a.) Standard surgery, b.) Surgery with aECoG tailoring or c.) Long-term/chronic ECoG monitoring preceding the resective surgery. The discussion of the LWEC automatically determines the in- (group b) and exclusion criteria (group a & c). The (coordinating) researcher, together with the Physician Assistant and/or Nurse practitioner, checks if patients from group b comply with the other in- and exclusion criteria. This check can be done using the patient information available by the LWEC meetings and neurologist and surgeon. Patients will only be approached for participation in this study if they comply with the in- and exclusion criteria.

[2] The remaining 20-25 patients are operated in UMC Maastricht, of which 5-10 undergo aECoG during surgery. The UMC Maastricht is not included in this national multi-center RTC due to practical reasons (limited number eligible patients, different software and distance from Utrecht).

3.6.3 Inclusion criteria

- Patients of all ages with:
  Focal Epilepsy that are planned for neurosurgery with aECoG, with the goal of tailoring the resection.

  - Command of Dutch language of the patient/parents/legal representatives and capability of completing the questionnaires (by email or phone).

3.6.4 Exclusion criteria

Exclusion criteria are:

- Patients who underwent chronic ECoG monitoring preceding epilepsy surgery (grids). This is a biased population, since the results of the extensive pre-surgical work-up as well as the results of the cECoG monitoring period are included in the final decision making regarding the resection, and a precise seizure onset zone as well as spike and HFO area are known.

- Patients with an occipital focus undergoing aECoG.
  Currently, it is not possible to discriminate between pathological or physiological occipital HFOs, and thus unsafe to perform HFO guided resections in patients with a presumed occipital focus.

- Patients with tailored tumor surgery with expected progressive course of disease (e.g. expected tumor growth, planned radiotherapy after surgery).

3.6.5 Sample size calculation

The sample size calculation is based on the following: a success percentage of 65% is observed for spikes for seizure freedom and an expected success percentage of 80% for HFOs (Jacobs, LeVan et al. 2008; Jacobs, Zijlmans et al. 2010; Wu, Sankar et al. 2010).

The future perspective of the use of HFOs lies in three aspects; *1.) expected improved chance of seizure freedom; 2.) expected decrease in tissue to be removed during surgery; 3.) the development of a fast automatic and objective detection device for HFOs based on a few electrodes.* This device gives onsite information to the surgeon, and would yield faster and better epilepsy surgery. Spikes will always require time- and labor intensive clinical observation, which depends on the experience and subjective interpretation of the clinical neurophysiologist.

Primary outcome in epilepsy surgery is seizure freedom. We expect an advantage using HFOs. However, a big international RCT is needed to prove superiority of HFOs over spikes. Acceptance by surgeons and differences in decisions about eligibility, surgery techniques and EEG software between centres complicates the practical feasibility of such a multicenter RCT. Both the knowledge that HFO based surgery will not lead to decreased chance of seizure freedom, and the development and utility of a detection device are needed to come to such an international RCT.

Our aim is to demonstrate in this pilot study that the prospective use of HFOs is possible and does not lead to a lower chance of seizure freedom than the current methods based on spikes, although we expect removal of less tissue. We therefore propose a non-inferiority design, based on the primary outcome variable “seizure freedom”. Our hypothesis is that HFOs perform not worse and maybe even better than epileptiform spikes in achieving seizure freedom. The argumentation for this non-inferiority design and the sample size calculation are based on the primary outcome variable ‘seizure freedom’. We also expect removal of less tissue. This outcome measure does not qualify as a primary outcome measure, as neurosurgeons will not change their strategy if the main goal of surgery –seizure freedom- is not achieved at least as often.

The sample size calculation is based on a success percentage in the control group (spikes) of 65%, and the expected success percentage in the experimental group (HFOs) of 80%. The resulting margin is 15%. Using a one sided 95% confidence interval (5% significance level) and a non-inferiority limit (d) of 10%, to acquire an 80% power we need 39 patients per group. Resulting in a total of 78 patients.

Study completion is reached when 78 patients are included in the study, regardless of inclusion period. The study was initially set-up as a two-center study at the UMC Utrecht and VUmc Amsteram and based on previous numbers of ECoG-guided surgeries, the expected inclusion rate was expected to be around 25 patients/year in the UMC Utrecht and 20 patients/year in the VUmc Amsterdam (assuming ± 80% participation). See table 1. Because of changes in collaboration and closure of the study site VUmc, we expect to include 35 patients a year for 2016 till 2018 at the UMC Utrecht.

3.7 Treatment of subjects

3.7.1 Investigational treatment

During epilepsy surgery the final amount of tissue to be resected is guided by the epileptic activity seen in the aECoG. We compare the use of a new aECoG biomarker for epileptogenic tissue, HFOs, in comparison to the current aECoG biomarker, epileptiform spikes. Independent of the randomisation, ictiform spike patterns will always be resected.

3.7.2 Detection of HFOs

To enable evaluation of the HFOs in the aECoG in the operating room the clinical EEG software used (System Plus evolution, Micromed, Venice) has been updated with additional frequency filter settings for HFO assessment (conform to the Stellate Harmonie Software used in various previous studies (Jacobs, LeVan et al. 2008; Zijlmans, Jacobs et al. 2009; Wu, Sankar et al. 2010; Zijlmans, Jacobs et al. 2011). This software enables visual assessment while recording, that will be followed by visual re-evaluation of the recorded data by 'the dedicated decision-making team' (see paragraph **Fout! Verwijzingsbron niet gevonden.**), during a standard surgical time out. An additional laptop with this software will be connected to the EEG-console in the operating room. We expect that the visual analysis of HFOs will require 50% more time compared to the visual analysis of epileptogenic spikes (for more details see paragraph **Fout! Verwijzingsbron niet gevonden.**).

3.7.3 The dedicated team

A dedicated team consisting of a clinical neurophysiologist/neurologist, a physician assistant and a researcher (principal investigator or (coordinating) researcher) will evaluate the recorded aECoG during surgery. This team will advise the neurosurgeon on the extent of tissue to be resected, based on the aECoG biomarker of the randomized arm (HFO or spike). In clinical practice a time-out during the surgical procedure is taken to assess the location of the epileptogenic spikes. Likewise this time-out will be used to evaluate the aECoG for HFOs. In the 6 months preceding the start of the study, the team will be trained in visual assessment and interpretation of HFOs on the basis of available retrospective data, by the principal investigator and the researcher.

3.7.4 Use of co-intervention and escape medication

There will be no modifications in the regular work-up for epilepsy surgery, anaesthesia, surgical risk containment or follow-up. Patients are allowed to use their regular anti-epileptic drugs.

3.8 Methods

3.8.1 Study parameters/endpoints

3.8.1.1 Main study parameters/endpoints

♦ Outcome

Main study parameter is outcome after epilepsy surgery at 1 year follow-up using the Engel classification. This classification consists of four categories; Ia=no seizures, Ib=only rare auras, Ic=some disabling seizure after surgery, but free of disabling seizure for at least 2 years, II=rare seizures, III= worthwhile improvement and IV= no worthwhile improvement.(Engel, C. et al. 1993).

To simplify analysis, outcome scores will be dichotomized in two categories: total seizure freedom (Engel Ia+Ib) versus seizure recurrence (Engel Ic–IV). To enable interim analysis of the outcome in terms of seizures, we will determine preliminary post-surgical outcomes at 6-8 weeks and 6 months. A final outcome will be determined after 12 months. This will require the patients to fill in an additional short questionnaire on their seizure frequency at pre-surgical baseline (after signing informed consent), and the same questionnaire via email/phone at 6-8 weeks, 6 and 12 months post-operatively (See F1 of the dossier).

So called 'running down' seizures, seizures that occur in the first 2 weeks after surgery are not considered as seizure recurrence.

3.8.1.2 Secondary study parameters/endpoints

♦ Volume of resected tissue

With HFOs being a more specific and sensitive biomarker for epileptogenic tissue than spikes, potentially the resection size could become smaller or larger. Therefore, the volume of resected tissue (in cm^3) is investigated as a secondary parameter. The amount of resected tissue is determined by voxel-based volumetrics of the pre- and post-surgical MRI using Curryscan7 Neuroimaging Suite (Compumedics Neuroscan, Hamburg, GER).

♦ Neurological deficits

Neurological changes (e.g visual field defects, hemiparesis, word finding difficulties) can be divided into pre-existing, aggravated/improved, or new deficits, and can be anticipated or unexpected. The changes in/severity of neurological deficits will be classified/quantified using the National Institutes of Health Stroke Scale (NIHSS) (see also paragraph **Fout! Verwijzingsbron niet gevonden.**) before surgery (after signing informed consent) and prior to discharge out of hospital by the researcher. This will be repeated by the doctor in charge/neurologist/neurosurgeon after 6-8 weeks, 6 months and 12 months in case of initial neurological changes/deficits, until the deficits disappeared or the end of follow-up.

The NIHSS is composed of 11 items (including visual field, motor function, speech), each of which scores a specific ability between a 0 and 4. For each item, a score of 0 typically indicates normal function in that specific ability; while a higher score is indicative of some level of impairment. The patient's total NIHSS scores is calculated by summation of all scores (NIHSS score range 0-42). A difference of 1 point on the NIHSS scale between 2 tests is considered clinically relevant in our population of epilepsy patients.

♦ Cognitive functioning

Comparison of test results of pre- and post-operative (6 or 12[3] months) neuropsychological investigation (NPO). This routine neuropsychological investigation includes testing of IQ (verbal and performal), working memory and processing speed. All tests conform to the age and abilities of the patients, but report on the same domains. Per domain, individual patients' results will be dichotomized into negative/no/positive change compared to pre-surgical performance. This enables comparison of all patients, independent from age, at group level. Note: The neuropsychologists fill in a form for the researchers with the required information for the study.

♦ **Health related Quality of life (HRQOL)**
Health related quality of life tests (HRQOL, QUOLI89 for adults and an age adapted comparable list for children) are no longer performed pre-and post-operatively (6 or 12 months) during routine neurophysiological investigation (NPO). Therefore, we will use as endpoint the (interim) assessment of HRQol that is enabled by a visual analogue scale (VAS) on overall self-perceived health (including their epilepsy) included in the baseline and follow-up questionnaire. This VAS scale is also part of the HRQoL tests.

♦ **Procedure duration**
Post-hoc analysis of duration of surgery (minutes) and aECoG recording time (minutes).

♦ **Complications related to study procedures**
Accounts are kept of the number of (post-)operative complications, such as bleeding, infection, unexpected or aggravated neurological deficits. These events will also be reported as (S)AE. See paragraph 9.2 for more details on risk of complications in epilepsy surgery in literature and number of complications encountered in the UMCU the last few years. Monitoring and interim analysis by the DSMB will be based on these numbers (see section K5 of this dossier), as well as end-point determination (see paragraph **Fout! Verwijzingsbron niet gevonden.** and **Fout! Verwijzingsbron niet gevonden.**).

3.8.2 Randomisation, blinding and treatment allocation

When a patient, after a full pre-surgical work-up, is put on the waiting list for epilepsy surgery, the patients will be screened by the neurologist or PA for eligibility for the study and asked to be introduced to the researcher. Eligible epilepsy surgery patients (and/or their relatives) will be informed about this study by the researcher in person, and asked for informed consent on the day prior to the day of surgery in the UMCU, IC is asked minimal 1 week later than the informative talk with the researcher (see paragraph 11.2 for details on the informed consent procedure). In the week prior to the planned day of surgery the researcher will call the patients to give them the opportunity to ask questions and to summarize the procedures.

If a patient wants to participate, IC will be signed on the day of hospital admission, the day prior to the day of surgery. Subsequently baseline questionnaire and NIHSS will be taken and the coordinating investigator will randomize the patient. Randomization will be performed by means of online randomization software (ALEA) provided by Research Bureau VUmc. This will include stratification for epilepsy type (Temporal Lobe epilepsy (TLE) vs. extra-TLE). We will use a random small block randomisation of blocks of 2, 4 and 6 patients.

Since knowledge of the treatment allocation can bias the follow-up results, especially in HRQOL assessment, patients as well as caregivers/physicians/psychologist involved in the follow-up

[3] Timing depends on center

procedures will be blinded for the treatment allocation. This blinding can be guaranteed as the KNF report in the Electronic Patient Dossier will be blinded for the period of follow-up of the patient. Blinding of the treating physicians/neurosurgeons for treatment allocation is considered not feasible because of the character of the intervention. When the endpoint of the study is reached the patients and involved caregivers/physicians/psychologist/general practitioner are informed about the performed intervention. Premature termination of the study will lead to breaking of the randomization code.

3.8.3 Study procedures

3.8.3.1 Pre-intervention procedures

♦ Pre-operative workup (standard)

The standard clinical pre-operative workup includes:

- A pre-operative 3D whole head MRI following dedicated epilepsy protocol (Meiners 2002)
- Neurological examination (by means of NIHSS)
- NPO examination
- (video) EEG
- Visual field examination, when indicated.

Data will be retrieved from the electronic patient file (EPD) by the principal investigator and (coordinating) researcher(s).

♦ Informed consent (additional)

See paragraph **Fout! Verwijzingsbron niet gevonden.**. After the informed consent is given, the patient is requested to fill in the baseline questionnaire about seizure frequency and quality of life.

3.8.3.2 Intervention

The study intervention is limited to the moments when aECoG is recorded and assessed to determine the final extent of the cortical resection. There may be typically two or three such moments during surgery.

The site of surgery is determined based on pre-surgical information. The cortex is exposed by trepanation and opening of the dura mater. At this point the first aECoG recording session starts. One or more electrode grids with inter-electrode distance of 1 cm are placed over the exposed area. Their position is sketched on paper and photographs are taken. Propofol anaesthesia is interrupted for 10-15 minutes during which aECoG recording takes place. Patients will not unintendedly wake up as physiological parameters are watched by the anaesthesiologist. The dedicated team will advise the neurosurgeon on the aECoG biomarker findings. These findings have then to be weighed against (a) potential involvement of functional cortex and (b) practical issues such as feasibility and risks from vascular anatomy. The neurosurgeon then decides on the resection he is willing to undertake, and proceeds with surgery. The neurosurgeon decides, at any given moment, to perform interim aECoGs recording to guide further decision making following the same procedure as stated above. Finally, after completion of the resection, a last aECoG is recorded and the end result of surgery is photographed. Note that this procedure is not altered by this study, except for the used criteria for what constitutes the epileptiform biomarker in the aECoG. In half of the patients, this will be HFOs (arm 1) and in the other half the conventional epileptiform spikes (arm 2). We expect that the analysis of

HFOs in the aECoG will take probably 50% more time than the analysis of spikes. This means a prolongation of the operation time by approximately 5 minutes for each aECoG recording.

The dedicated team analyses the presence of spikes or HFOs; although HFOs cannot be evaluated without knowledge about the presence of artifacts in the 0-80Hz band (Zijlmans, Jiruska et al. 2012). In case of HFO assessment, the dedicated team advises the neurosurgeon about the location and extent of the resection taking only the HFOs and ictiform spike patterns in consideration.

The clinical reports by the clinical neurophysiologist and neurosurgeon will speak of ‘epileptogenic events’ instead of spikes and/or HFOs in order to preserve blinded randomisation of secondary caregivers. The reports will state that the patient is included in ‘the HFO study’.

Exceptional circumstances

This is an intention to treat study. Therefore, independent of the randomization, similar to standard procedure (spikes), HFOs in eloquent regions will not be resected. In group 2 (spikes), a planned resection without tailoring is done if no spikes are present. Likewise, a planned resection is performed if no HFOs are found or doubt exists about validity of the observed HFOs in group 1 (HFOs).

3.8.3.3 Post-interventional procedures

♦ Post-operative in-hospital

The hospitalized post-operative period is usually 5-10 days.
During this period are performed:

- Surgical complication monitoring
- Neurological examination post-operative before discharge (NIHSS).

♦ Post-operative follow-up

The standard post-operative follow-up consists of:
<u>Follow-up at 6-8 weeks (visit-margin of ±2 weeks):</u>

- Clinical neurological examination (neurological deficits=NIHSS*, preliminary outcome, FU-questionnaire incl HRQoL)

<u>At 2-3 months (no visit margin since no time-dependent variables):</u>

- A post-operative 3D whole head MRI following dedicated epilepsy protocol (Meiners 2002).
- Visual field examination, when indicated

<u>Follow-up at 6 months (visit margin of ±3 months):</u>

- Clinical neurological examination (neurological deficits=NIHSS*, preliminary outcome, FU-questionnaire incl HRQoL)
- NPO examination (cognitive function)

<u>OR Follow-up at 12 months (visit margin of ±3 months):</u>

- Clinical neurological examination (neurological deficits=NIHSS*, final outcome, FU-questionnaire incl HRQoL)
- NPO examination (cognitive function)

Data will be retrieved from the electronic patient file (EPD) by the principal investigator and (coordinating) researcher(s).

To facilitate interim analysis we will present patients the short follow-up (fu) questionnaire on seizure frequency in addition to a general HRQoL question (VAS scale) through internet/phone at 6-8 weeks, 6 months and 12 months post-operatively.

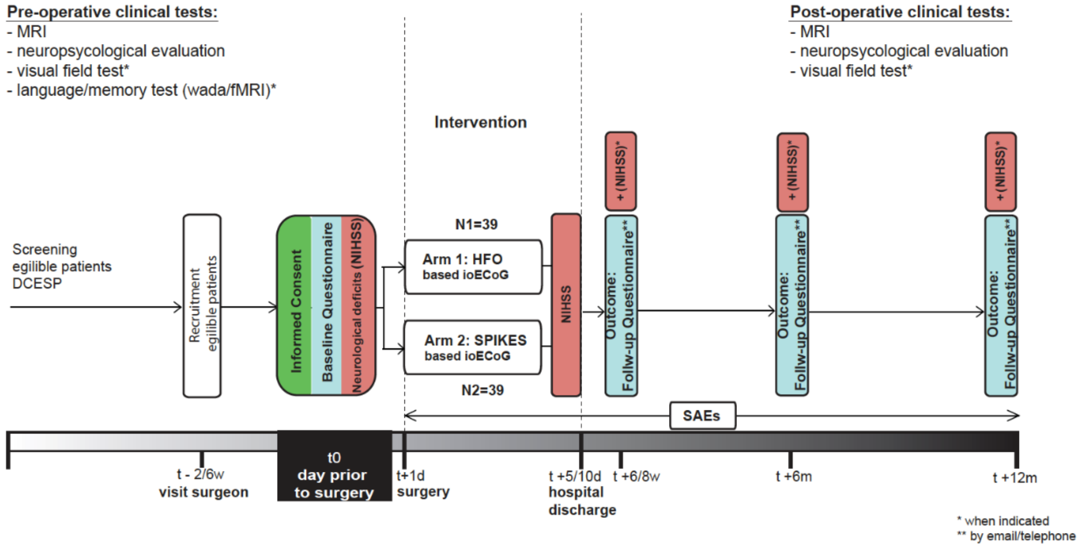

Figure 2: Timeline "The HFO study", with respect to the additional aspects for the patient compared to the normal clinical procedures during the epilepsy surgery trajectory. *NIHSS only when indicated

3.8.3.4 Withdrawal of individual subjects

Patients can withdraw from the study at any time for any reason if they wish to do so, without any explanation. The neurosurgeon can decide to withdraw a subject from the study for urgent medical reasons during surgery or in case of a second operation (re-do). The Code of conduct involving minors, as stated by the CCMO, will be followed in the assessment of objections by subjects from these groups.

3.8.3.5 Replacement of individual subjects after withdrawal

78 subjects are needed to ensure the power/significance of our findings. Our primary parameter is outcome after follow-up of 1 year. However, we will collect preliminary outcome at 6-8 weeks, 6 and 12 months to enable interim analysis. Therefore we need to replace subjects if they withdraw from the study during follow-up before the first preliminary outcome determination at 6-8 weeks. However, the number of subjects that withdraw after the initial intervention is expected to be low, since follow-up after surgery is routine procedure. Patients withdrawn from the study during the operation procedure by the surgeon/dedicated team need to be replaced.

3.8.3.6 Follow-up of subjects withdrawn from treatment

In case of withdrawal, treatment will be continued according to the standard treatment protocol of the UMCU. The collected patient data before moment of withdrawal will remain included in the statistical analysis. The last determined preliminary outcome classification will be used for analysis.

3.8.3.7 Premature termination of the study

Premature termination of the study is possible in case of:

1. A significant harmful effect of the intervention in arm 1 (HFOs) compared to arm 2 (spikes, the gold standard) at interim analysis (at 20 and 40 patients included). For details see interim analysis as described in the DSMB plan in section K5 of this dossier.
2. Advise to do so from the independent monitoring committee (see DSMB plan in section K5 of this dossier).

If the trial is to be terminated prematurely, the METC will be notified within 15 days of this decision. All personnel and subjects involved in the RCT will be informed. The study will be made public.

3.9 Safety reporting

3.9.1 Section 10 WMO event

In accordance to section 10, subsection 1, of the WMO, the investigator will inform the subjects and the reviewing accredited METC if anything occurs, on the basis of which it appears that the disadvantages of participation may be significantly greater than was foreseen in the research proposal. The study will be suspended pending further review by the accredited METC. The investigator will take care that all subjects are kept informed.

3.9.2 Adverse and serious adverse events

3.9.2.1 Adverse events (AEs)

Adverse events are defined as any undesirable experience occurring to a subject during the study, whether or not considered related to the experimental treatment. Most reported (serious) adverse events of epilepsy surgery are infections, and neurological deficits such as hemiparesis, visual field defects, and language disturbances (Tonini, Beghi et al. 2004). Neurological deficits can be divided into pre-existing, improved, aggravated or new deficits, and can be anticipated or unexpected.[4] The neurological deficits will be assessed prior to discharge out of hospital. Direct post-operative functional deficits are sometimes present due to surgical induced brain oedema, and will resolve prior to discharge; these will not be reported as SAE.

The expected adverse events related to the surgical intervention are complications, new seizures, worsening of the seizure frequency, infection or bleeding. These will be monitored through the primary and secondary outcomes or results in a SAE (admittance to hospital). We will therefore not separately report AEs during the post-operative follow-up period of 1 year, since there are very few relevant AEs that we can think off outside the previously mentioned that are covered. Second, we are dependent on self reportage by the patients (online questionnaire) and cannot rely on outward hospital visits, which results in very subjective reportage. Third, it is no longer obliged to report AEs in all trials.

Additional remark: A recent publication reported the incidence of per- and post-operative adverse events of epilepsy surgery in US in the period 1990-2008: neurological complications/stroke (1-4%), post-operative infection (2-4%), intracranial bleeding (1-3%), status epileptic (≤1%), and pulmonary complications (1-3%). Cardiac complications, embolisms and death are incidental (Englot, Ouyang et al. 2013). Mortality in epilepsy surgery is seldom observed.

In the UMCU in 2011 a total of 52 patients underwent surgery with aECoG (including chronic ECoG evaluated patients), in 2 patients complications occurred (psychosis, paralysis leg and arm). In 2012 a total of 47 patients underwent surgery with aECoG, in 4 patients complications occurred (speech disturbance, 3x paralysis arm or leg). A complication was defined as an unexpected deficit after surgery. Minor visual field deficits, anticipated in TLE surgery, were not included.

[4] Note that: The majority of epilepsy surgeries is performed in areas with adjacent functionally specialized brain areas. Desirably, the neurosurgeon takes a safety margin of one sulcus into account. However, sometimes he is forced to exceed this boundary or has pre-surgically decided to sacrifice a certain functional area. In those cases this is done with mutual agreement of patient and surgeon. The surgical risk on functional damage depends on the resected area. Pre- and intra-operative function localization will be performed, and the same safety rules regarding these eloquent regions are valid for HFOs as for current (spike) procedure.

3.9.2.2 Serious adverse events (SAEs)

A serious adverse event is any untoward medical occurrence or effect that at any dose:

- results in death;
- is life threatening (at the time of the event);
- requires hospitalization or prolongation of existing inpatients' hospitalization;
- results in persistent or significant disability or incapacity;
- is a new event of the trial likely to affect the safety of the subjects.

For the present study, a SAE is defined according to the definition above. SAEs will be closely monitored by the (coordinating) researcher within the timeframe of hospital admission (normal range ≤10 days after surgery) and monitored with the follow-up questionnaires and by the physician in charge during the follow-up period. If a SAE occurs, the principal investigators will be notified by e-mail or telephone within 24 hours, independent of the site.

All SAEs will be reported to the DSMB and the central METC, according to their requirements. All SAEs will be reported through the web portal *ToetsingOnline* to the accredited METC that approved the protocol. The reporting will occur within 15 days after the investigator has first received information on the SAE. For fatal or life-threatening cases a preliminary report will be offered within 7 days followed by a complete report within 8 days.

3.9.3 Follow-up of adverse events

Relevant adverse events (related to the intervention) will be followed until they have abated, or until a stable situation has been reached. This is incorporated in the study protocol. Depending on the event, follow up may require additional tests or medical procedures as indicated, and/or referral to the general physician or a medical specialist.

3.9.4 Data Safety Monitoring Board (DSMB)

During the inclusion period, an independent and external Data Safety Monitoring Board will analyse the incidence of the above mentioned serious adverse events, in accordance with the "DSMB plan" (See section K5 of this dossier). DSMB meetings and following recommendations will be based on the interim analysis performed by the DSMB statistician. For a detailed description of the tasks and obligations, and stopping rules of the DSMB, see the DSMB plan. The advice(s) of the DSMB will be notified to the METC that approved the protocol. With this notification a statement will be included indicating whether the advice will be followed.

3.9.5 Monitoring

The surgical risks of tailored resection by HFOs do not directly differ compared to the current resection based on spikes in the aECoG. Resection side and size are largely determined by the results of the pre-operative work-up. However, the use of a new biomarker for guiding the extent of the resection poses a risk in performing a somewhat smaller or larger resection than intended, with the accompanying two-sided risk of increasing or decreasing the chances on seizure freedom. Since also children are included, according to the directive by the NFU (Nederlandse Federatie van Universitair medische centra), this study is to be classified as a 'moderate risk study'. The monitoring of this study will be performed according to this classification. Monitoring will be performed by the assigned monitor of the UMCU and will be performed according to a pre-defined monitor plan. For a detailed description of the monitoring plan, please see section K6 of this dossier.

3.9.6 Stopping rules

Study completion will be at inclusion plus 1 year follow-up of 78 patients. The stopping rules for this trial can be found in the “DSMB plan” (see section K5 of this dossier), and in paragraph **Fout! Verwijzingsbron niet gevonden.** ‘Premature termination of the study’.

3.10 Statistical analysis

All patients who are randomly assigned treatment will be included in the following analyses.

3.10.1 Primary outcome and Secondary outcome

The primary ‘intention to treat’ analyses at study completion are based on the difference between the treatment arms with respect to surgical outcome after 12 months. The primary outcome is a categorical outcome, total seizure freedom vs. seizure recurrence. Risk ratios (RR) and risk difference with 95% confidence interval will be calculated (chi-square).

Secondary outcomes are the amount of tissue resected (continuous variable), neurological deficits (categorical variable), quality of life (continuous variable), and cognitive functioning (IQ continuous variable, other domains categorical variable, scored as negative/no/positive improvement). For the secondary outcome parameters consisting of continuous variables, a T-test with 95% confidence interval is calculated and tested. For the secondary outcome parameters consisting of categorical variables, the RR and risk difference with a 95% confidence interval are calculate using a Chi-square test.

Regression analysis, linear regression for continuous variables and logistic regression for categorical variables, is performed 1.) to stratify for epilepsy type and 2.) to investigate if there are relationships with demographic/subject variables, such as age, gender, and pathology, or with experimental variables, such as the duration of the operation, the surgeon and the amount of anesthesia received. Regression analysis is also performed between primary outcome (post surgical outcome) and the secondary outcomes.

A post hoc analysis for duration of surgery and aECoG recordings, and complications will be performed. In the logistic regression analysis we will, after screening for the number of missing data, perform multiple imputation. For the primary and secondary endpoints no imputation will be performed. Statistical analysis will be performed in SPSS version 21 or higher and/or R version 3.1 or higher.

The number of eligible patients withholding consent will be registered, and demographic information is collected anonymously for post-hoc explorative analysis.

3.10.2 Interim analysis

The biostatistician of the DSMB will perform a primary **safety** interim analysis on the first 20 and 40 included patients and the occurrence of surgery complications and SAE. **Efficiency** analysis is performed once: during the interim analysis of 40 patients (halfway inclusion). This requires an alpha correction for the efficiency analysis using the O’Brien Fleming method. The test will be performed with an alpha = 0.003 (one-sided because of the non-inferiority design). Premature termination of the study is possible in case of significant (p-value <0.003) harmful effects in patients that obtained seizure freedom in arm 1 (HFOs) compared to arm 2 (spikes, current standard standard) at interim analysis. The DSMB may for various reasons decide to perform additional analyses. Guidance for those is provided in the DSMB plan, section 8 paragraph “Formal statistical methods”; see section K5 of this dossier.

For the planned interim analysis the available (preliminary) post-surgical outcome from the additional follow-up questionnaire at 6-8 weeks, 6 and 12 months will be used. The most recent available post-surgical outcome for each patient will be used during interim analysis. This means that during first interim analysis (20 patients) e.g. that for patient #1 preliminary outcome at 6 months will be used and for patient #20 the preliminary outcome at 6-8 weeks.

3.11 Ethical considerations

3.11.1 Regulation statement

The study will be conducted according to the principles of the Declaration of Helsinki (version of 2008) and in accordance with the Medical Research Involving Human Subjects Act (WMO) and other guidelines, regulations and Acts. Data management, monitoring and reporting of the study will be performed in accordance with the ICH GCP guidelines.

3.11.2 Recruitment and consent

During the intake visit the neurosurgeon asks eligible patients if a researcher may approach them about the study. Subsequently, the researcher will inform the patient about the study in a meeting scheduled directly after the patient's intake visit with the neurosurgeon. An information letter with an informed consent form is provided to the patient and/or parents/legal representatives (See Section E1 & E2 of the dossier). In the week prior to the planned surgery the researcher will call the patients to give them the opportunity to ask questions and to summarize the procedures.

Informed consent will be asked by the researcher in person during a second appointment minimal 1 week after the intake visit, on the day of hospital admission, one day prior to surgery. When informed consent is obtained, the referring physician and general practitioner are informed about the patient participating in the study (See Section K6 of the dossier).

In case the patient is younger than 18 years, parents/legal representatives will be asked for informed consent. Of children >12 years, who are not severely mentally retarded, not only the parents'/legal representatives' consent but also the child's consent will be asked.

If necessary, further explanation is provided through telephone, e-mail depending on the need expressed by the patient, caregivers or legal representatives. Patients who do not consent will be treated according to the standard epilepsy surgery protocol.

3.11.3 Objection by minors

This study also includes minors (age 0-18 years). In the case of a minor, both the minor and both of the parents will be sent a letter. The minor will only be included when he/she and both of his/her parents approve on the participation and sign informed consent. The Code of conduct involving minors will be followed in the assessment of objections by subjects from these groups. Clearly stated in the information letter is that the representatives are free to refrain from participation in the study.

3.11.4 Benefits and group relatedness

The intervention under study will affect the surgical strategy only. Use of a more precise biomarker as HFOs in the aECoG might increase the success rate of epilepsy surgery while decreasing complication risk, quicker revalidation, diminished neurological and cognitive deficits and improve overall quality of life compared to the current intervention based on spikes. We expect that the surgical risks for the intervention are the same as for the current golden standard. Standard safety procedures are followed. The involved neurosurgeons are highly experienced in epilepsy surgery. In the last years the surgical complication risks during the standard procedure proved to be low. In the period 2011-2012, 6 complications (psychosis, deficits in motor function of arm and/or leg, or speech problems) were reported on a total of 97 surgeries. With these numbers, the overall chance on complications in the UMCU is 6%, which is comparable with the risks reported in literature (Tonini, Beghi et al. 2004; Englot, Ouyang et al. 2013). See for more details paragraph **Fout! Verwijzingsbron niet gevonden.**.

Why include children?

The incidence of epilepsy is highest in the paediatric and adolescent population (70/100 000 versus 30/100 000 in adults) (Forsgren, Beghi et al. 2005). In recent years, the importance of early epilepsy surgery has been stressed. Children with intractable epilepsy should be considered for early epilepsy surgery, since shorter epilepsy duration is correlated with a better prognosis regarding post-surgical and cognitive outcome (Jeong, Lee et al. 1999; Engel, McDermott et al. 2012; Simasathien, Vadera et al. 2013). This is the reason for a current increase in the number of childhood epilepsy surgeries and a decrease in adult epilepsy surgeries.

Epilepsy surgery can significantly improve HRQOl and set the stage for normalized social development within the first 6 months after surgery (van Empelen, Jennekens-Schinkel et al. 2005). Therefore, paediatric population would benefit most of seizure freedom when accompanied with quick revalidation, less neurological deficits and improved quality of life.

Wu et al (2010) already demonstrated in a retrospective study that HFOs are present in aECoG in 80% of the paediatric epilepsy cases and complete resection of tissue containing FRs was strongly correlated with postoperative seizure freedom. Akiyama et al (2011) reported this same finding for ictal HFOs, and additionally found that resection of the SOZ did not influence seizure outcome nor did surgical resection size (Akiyama, McCoy et al. 2011). Several other retrospective studies in paediatric populations confirm these findings, independent of the underlying cause of the epilepsy and for both ictal as interical HFOs (Ochi, Otsubo et al. 2007_ENREF_24; Jacobs, Zijlmans et al. 2010; Wu, Sankar et al. 2010; Akiyama, McCoy et al. 2011; Nariai, Matsuzaki et al. 2011; Fujiwara, Greiner et al. 2012).

Adult surgery is more and more considered as ‘delayed child surgery’ as in most patients refractory epilepsy usually evolves in childhood and early adolescence (Forsgren, Beghi et al. 2005; de Boer, Mula et al. 2008). The percentage of paediatric epilepsy surgery will increase in the coming years.

3.11.5 Compensation for injury

The investigator has a liability insurance, which is in accordance with article 7, subsection 6 of the WMO.

The sponsor (also) has an insurance, which is in accordance with the legal requirements in the Netherlands (Article 7 WMO and the Measure regarding Compulsory Insurance for Clinical Research in Humans of 23th June 2003). This insurance provides cover for damage to research subjects through injury or death caused by the study.

1. € 450.000,-- (i.e. four hundred and fifty thousand Euro) for death or injury for each subject who participates in the Research;
2. € 3.500.000,-- (i.e. three million five hundred thousand Euro) for death or injury for all subjects who participate in the Research;
3. € 5.000.000,-- (i.e. five million Euro) for the total damage incurred by the organisation for all damage disclosed by scientific research for the Sponsor as ‘verrichter’ in the meaning of said Act in each year of insurance coverage.

The insurance applies to the damage that becomes apparent during the study or within 4 years after the end of the study.

The insurance polis of the UMCU regarding damage to research subjects, the ‘proefpersonenverzekering’, can be found in section G1 of this dossier. The UMCU liability insurance is provided in section G2 of this dossier.

3.12 Administrative aspects and publication

3.12.1 Handling and storage of data and documents

An Investigator Site File and a Trial Master File will be set up by the coordinating investigator in the beginning of the study. The list of essential documents will be in accordance with the GCP-guidelines. The essential documents that make up the file will be stored in a secure but accessible manner. All essential documents will be legible and accurate. The key documents for randomization will be kept separated from the accumulating data by the research nurse, who is involved in the study procedures and data accumulation.

For each randomized patient an online Case Record Form (CRF) will be completed. The relevant aECoG data will be stripped from all personal details and receive a unique patient-code in combination with a center identification number that cannot be traced to a patient. The decryption key is only accessible to the (coordinating) researcher(s). Trial personnel will not pass names outside the hospital. The research coordinator will insure that patients' anonymity is maintained. The subject identification code list will be safeguarded by the principal investigator.

Central data management will be performed by the (coordinating) investigator. CRF data will be digitalized into a dedicated Open clinica & NETQ database (support: ICT divisie Hersenen UMCU). In this database patient information and accumulating data will be stored. All data will be stored at the investigational location according to the guidelines, and will be kept for 15 years. A description of the data monitoring can be found in the Monitoring Plan, section K6 of this dossier.

3.12.2 Amendments

Amendments are changes made to the investigation after a favourable opinion by the accredited METC has been given. A ‘substantial amendment’ is defined as an amendment to the terms of the METC application, or to the protocol or any other supporting documentation, that is likely to affect to a significant degree:

1. The safety or physical or mental integrity of the subjects of the trial;
2. The scientific value of the trial;
3. The conduct or management of the trial; or
4. The quality or safety of any intervention used in the trial.

All substantial amendments will be notified to the METC that gave a favourable opinion. Non-substantial amendments (e.g. correction of an error in writing) will not be notified to the accredited METC, but will be recorded and filed by the investigators.

3.12.3 Annual progress report

The investigator will submit a summary of the progress of the trial to the accredited METC once a year. Information will be provided on the date of inclusion of the first subject, numbers of subjects included and numbers of subjects that have completed the trial, serious adverse events/ serious adverse reactions, other problems, and amendments.

3.12.4 End of study report

The investigator will notify the accredited METC of the end of the study within a period of 8 weeks. The end of the study is defined as the end of the study period for the last patient.

Information will be provided on the date of inclusion of the first subject, numbers of subjects included and numbers of subjects that have completed the trial, serious adverse events, other problems, and amendments.

In case the study is ended prematurely, the investigator will notify the accredited METC, including the reasons for the premature termination. Within one year after the end of the study, the investigator/sponsor will submit a final study report with the results of the study, including any publications/abstracts of the study, to the accredited METC.

3.12.5 Public disclosure and publication policy

The study will be entered into the international registry for clinical trials (register@clinicaltrials.gov). There are no restrictions on the publication of data and results from the sponsor the National Dutch Epilepsy Foundation (NEF). The results of the study will be published in one or more appropriate international peer reviewed journal(s). Co-authorship will require a reasonable contribution to the data collection.

3.13 References

Akiyama, T., B. McCoy, et al. (2011). "Focal resection of fast ripples on extraoperative intracranial EEG improves seizure outcome in pediatric epilepsy." Epilepsia **52**(10): 1802-1811.

Alarcon, G., J. J. Garcia Seoane, et al. (1997). "Origin and propagation of interictal discharges in the acute electrocorticogram. Implications for pathophysiology and surgical treatment of temporal lobe epilepsy." Brain **120 (Pt 12)**: 2259-2282.

Bragin, A., J. Engel, Jr., et al. (1999a). "Hippocampal and entorhinal cortex high-frequency oscillations (100--500 Hz) in human epileptic brain and in kainic acid--treated rats with chronic seizures." Epilepsia **40**(2): 127-137.

Bragin, A., J. Engel, et al. (1999b). "High-Frequency Oscillations in Human Brain." Brain Research **142**: 137-142.

Bragin, A., C. L. Wilson, et al. (2004). "High-frequency oscillations after status epilepticus: epileptogenesis and seizure genesis." Epilepsia **45**(9): 1017-1023.

de Boer, H. M., M. Mula, et al. (2008). "The global burden and stigma of epilepsy." Epilepsy Behav **12**(4): 540-546.

Engel, J., P. C., et al. (1993). Outcome with respect to epileptic seizures. Surgical Treatment of Epilepsies. J. jr. Engel. New York, NY, Raven Press**:** 609-621.

Engel, J., Jr. and F. L. da Silva (2012). "High-frequency oscillations - Where we are and where we need to go." Prog Neurobiol.

Engel, J., Jr., M. P. McDermott, et al. (2012). "Early surgical therapy for drug-resistant temporal lobe epilepsy: a randomized trial." Jama. **307**(9): 922-930. doi: 910.1001/jama.2012.1220.

Engel, J., Van Ness, P.C., Rasmussen, T.B. et al. (1993). "Outcome with respect to epileptic seizures. In Engel J Jr, ed. Surgical treatment of epilepsies. New York, NY: Raven Press;." 609-621.

Englot, D. J., D. Ouyang, et al. (2013). "Relationship between hospital surgical volume, lobectomy rates, and adverse perioperative events at US epilepsy centers." J Neurosurg **118**(1): 169-174.

Ferrier, C. H., E. Aronica, et al. (2006). "Electrocorticographic discharge patterns in glioneuronal tumors and focal cortical dysplasia." Epilepsia **47**(9): 1477-1486.

Forsgren, L., E. Beghi, et al. (2005). "The epidemiology of epilepsy in Europe - a systematic review." Eur J Neurol **12**(4): 245-253.

Fujiwara, H., H. M. Greiner, et al. (2012). "Resection of ictal high-frequency oscillations leads to favorable surgical outcome in pediatric epilepsy." Epilepsia **53**(9): 1607-1617.

Jacobs, J., P. LeVan, et al. (2008). "Interictal high-frequency oscillations (80-500 Hz) are an indicator of seizure onset areas independent of spikes in the human epileptic brain." Epilepsia **49**(11): 1893-1907.

Jacobs, J., P. Levan, et al. (2009). "High frequency oscillations in intracranial EEGs mark epileptogenicity rather than lesion type." Brain **132**(Pt 4): 1022-1037.

Jacobs, J., R. Staba, et al. (2012). "High-frequency oscillations (HFOs) in clinical epilepsy." Prog Neurobiol.

Jacobs, J., M. Zijlmans, et al. (2010). "High-Frequency Electroencephalographic Oscillations Correlate With Outcome of Epilepsy Surgery." Annals of Neurology **67**: 209-220.

Jeong, S. W., S. K. Lee, et al. (1999). "Prognostic factors in anterior temporal lobe resections for mesial temporal lobe epilepsy: multivariate analysis." Epilepsia. **40**(12): 1735-1739.

Kumar, A., C. Juhasz, et al. (2010). "Objective detection of epileptic foci by 18F-FDG PET in children undergoing epilepsy surgery." J.Nucl.Med. **51**(12): 1901-1907.

Meiners, L. C. (2002). "Role of MR imaging in epilepsy." Eur.Radiol. **12**(3): 499-501.

Nagasawa, T., C. Juhasz, et al. (2012). "Spontaneous and visually driven high-frequency oscillations in the occipital cortex: intracranial recording in epileptic patients." Hum Brain Mapp **33**(3): 569-583.

Nariai, H., N. Matsuzaki, et al. (2011). "Ictal high-frequency oscillations at 80-200 Hz coupled with delta phase in epileptic spasms." Epilepsia **52**(10): e130-134.

Ochi, A., H. Otsubo, et al. (2007). "Dynamic changes of ictal high-frequency oscillations in neocortical epilepsy: using multiple band frequency analysis." Epilepsia **48**(2): 286-296.

Palmini, A., A. Gambardella, et al. (1995). "Intrinsic epileptogenicity of human dysplastic cortex as suggested by corticography and surgical results." Ann Neurol **37**(4): 476-487.

Rosenow, F. and H. Luders (2001). "Presurgical evaluation of epilepsy." Brain **124**(Pt 9): 1683-1700.

Sander, J. W. (2003). "The epidemiology of epilepsy revisited." Current Opinion in Neurology **16**(2): 165-170.
Schwartz, T. H., C. W. Bazil, et al. (2000). "Do reactive post-resection "injury" spikes exist?" Epilepsia **41**(11): 1463-1468.
Simasathien, T., S. Vadera, et al. (2013). "Improved outcomes with earlier surgery for intractable frontal lobe epilepsy." Ann Neurol **11**(10): 23862.
Stefan, H., R. Hopfengartner, et al. (2008). "Interictal triple ECoG characteristics of temporal lobe epilepsies: An intraoperative ECoG analysis correlated with surgical outcome." Clin Neurophysiol **119**(3): 642-652.
Tellez-Zenteno, J. F., R. Dhar, et al. (2005). "Long-term seizure outcomes following epilepsy surgery: a systematic review and meta-analysis." Brain **128**(Pt 5): 1188-1198.
Tonini, C., E. Beghi, et al. (2004). "Predictors of epilepsy surgery outcome: a meta-analysis." Epilepsy Res **62**(1): 75-87.
van Empelen, R., A. Jennekens-Schinkel, et al. (2005). "Health-related quality of life and self-perceived competence of children assessed before and up to two years after epilepsy surgery." Epilepsia **46**(2): 258-271.
Wiebe, S., W. T. Blume, et al. (2001). "A randomized, controlled trial of surgery for temporal-lobe epilepsy." N Engl J Med **345**(5): 311-318.
Worrell, G. and J. Gotman (2011). "High-frequency oscillations and other electrophysiological biomarkers of epilepsy: clinical studies." Biomark Med **5**(5): 557-566.
Wu, J. Y., R. Sankar, et al. (2010). "Removing interictal fast ripples on electrocorticography linked with seizure freedom in children." Neurology **75**(19): 1686-1694.
Zelmann, R., F. Mari, et al. (2012). "A comparison between detectors of high frequency oscillations." Clin Neurophysiol **123**(1): 106-116.
Zelmann, R., M. Zijlmans, et al. (2009). "Improving the identification of High Frequency Oscillations." Clin Neurophysiol **120**(8): 1457-1464.
Zijlmans, M., G. M. Huiskamp, et al. (2012). "Epileptic high-frequency oscillations in intraoperative electrocorticography: The effect of propofol." Epilepsia **53**(10): 1799-1809.
Zijlmans, M., J. Jacobs, et al. (2011). "Ictal and interictal high frequency oscillations in patients with focal epilepsy." Clin Neurophysiol **122**(4): 664-671.
Zijlmans, M., J. Jacobs, et al. (2009). "High-frequency oscillations mirror disease activity in patients with epilepsy." Neurology **72**(11): 979-986.
Zijlmans, M., J. Jacobs, et al. (2009). "High frequency oscillations and seizure frequency in patients with focal epilepsy." Epilepsy Res **85**(2-3): 287-292.
Zijlmans, M., P. Jiruska, et al. (2012). "High-frequency oscillations as a new biomarker in epilepsy." Ann Neurol **71**(2): 169-178.

4 Interim analysis IDMC

4.1 IDMC report: 20 patients included and FU of 6-8 weeks (dd 17-04-2016)

4.1.1 Study progression

Date official start of study (first inclusion) is 6-11-2014 in the UMC Utrecht.
Total population in study: 78 patients
Expected inclusion rate: ±45 patients/year (±25/year in UMCU, ±20/year in VUmc)
Expected inclusion rate ±4 patients/month
Note: currently the maximal capacity of dedicated team is ±4 patients/month.

Start in the VUmc was scheduled for Autumn 2015, but has been cancelled (see below).

4.1.2 Recruitment

In September 2014 the recruitment started in the UMC Utrecht. Since then:

- Approached patients:	40
- Included patients:	27
- 'Pending' patients:	1
- Patients not included, reason:	12
Decided not to participate after given information	11
Organizational problems	1

In total 8 other patients were identified as eligible patients during screening but were not approached at all because:

- they were not interested in receiving information about the study (n = 3).
- not enough members of the HFO team available at their planned date of surgery (n = 5).

4.1.3 Randomization

So far, in total 27 patients have been randomized. Seventeen out of the 27 patients were children.

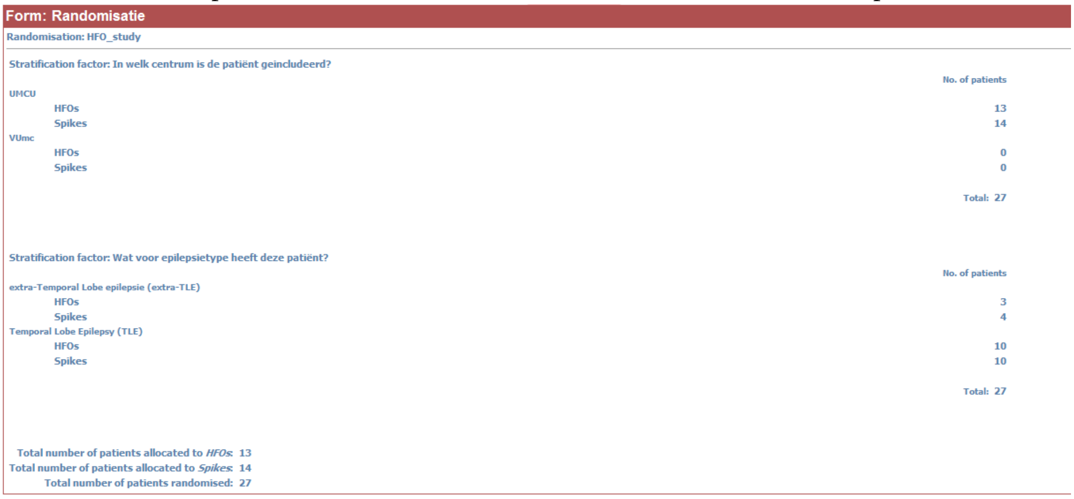
Form: Randomisatie
Randomisation: HFO_study

Stratification factor: In welk centrum is de patiënt geincludeerd?

	No. of patients
UMCU	
HFOs	13
Spikes	14
VUmc	
HFOs	0
Spikes	0
Total:	27

Stratification factor: Wat voor epilepsietype heeft deze patiënt?

	No. of patients
extra-Temporal Lobe epilepsie (extra-TLE)	
HFOs	3
Spikes	4
Temporal Lobe Epilepsy (TLE)	
HFOs	10
Spikes	10
Total:	27

Total number of patients allocated to *HFOs*: 13
Total number of patients allocated to *Spikes*: 14
Total number of patients randomised: 27

Figure S6: ALEA overview randomization first 20 patients.

4.1.4 Protocol violations

In total nine protocol violation were reported. Four protocol violations were reported about a shorter reflection time of less than 14 days, due to rescheduling of the surgical procedures. We explained the violation of reflection time to all these participants and/or parents and asked if they were fine with this. We randomized four patients that did not strictly comply with the initially defined inclusion criteria for refractory focal epilepsy; at least ≥2 experienced seizures in the past 24 months, in spite of two or more different anti-epileptic drugs (AEDs). The clinical indication for surgery has changed: (early) surgery is advised in case of developmental delays or side effects of AEDs. We adapted the in- en exclusion criteria (see in- & exclusion criteria in paragraph 1.4.1 & 1.42). The ninth protocol violation was about not reporting the adverse events over the period in which amendment nr 4 was written. In amendment nr 4 we declared that we would stop reporting adverse events (see above).

4.1.5 Treatment and follow-up

The total planned follow-up after surgery is 1 year. Additional follow-up questionnaires are assessed at 6-8 weeks, 6 and 12 months after surgery, e.g. to collect preliminary outcome information.
Post-surgical outcome is available for:

- 6-8 weeks: 24 patients
- 6 months: 6 patients
- 12 months: 3 patients

Lost during follow-up: 0

NOTE: From here on, we will report the results of the first 20 included patients of which first preliminary outcome at 6-8 weeks is available.

4.1.6 Baseline characteristics first 20 patients

	**Total (N=20)**	**HFO-arm (N=10)**	**Spike-arm (N=10)**
# Male/Female	7/13	3/7	4/6
# Adult/Child	8/12	5/5	3 /7
Mean + stdev age in years (range)	15.8 ±8.6 (2.0-31.0)	18.4±7.1 (9.0-30.0)	13.2±9.5 (2.0-31.0)
Mean duration epilepsy in months	81.8±83.2 (8.0-343.0)	100.2±100.1 (13.0-343.0)	63.3 ±62.1 (8.0-165.0)
TLE/extra-TLE	17/3	9/1	8/2
Region focus:<br>- *Temporal*<br>-*Frontal*<br>-*Central*<br>-*Parietal*	 17<br>3<br>0<br>0	 9<br>1<br>0<br>0	 8<br>2<br>0<br>0
# Auras/Seizures before surgery	11/17	4 /9*	7/8 **
Mean # Auras/month (range)	7.9±12.8 (0.0-45.0)	16.5±19.3 (4.0-45.0)	3.0±3.1 (0-9.0)
Mean # Seizures/month (range)	36.4±84.9 (0.0-336.0)	10.0±11.1 (1.0-35.0)	66.3±120.1 (0.0-336.0)

Table S6: baseline characteristics first 20 patients.

4.1.7 Serious adverse events first 20 patients

We encountered no complications related to study procedures. So far, two SAEs were reported.

#	Description Event	**Category** *(1=death 2=disability/incapacity 3= life-threatening 4= congenital anomaly/birth defect 5= hospitalization-initial or prolonged 6= required intervention to prevent permanent impairment 7= other)*	**Relation to study treatment** *(1=Unrelated, 2=Unlikely, 3=Possible, 4=Probable, 5=Definite)*	**Outcome of the SAE?** *1= Recovered 2= Recovered with sequelae 3= Continuing 4= Resulted in Death 5= Unknown*
#1	Readmittance (5 days) due to nausea and headache	5	2	1
#2	Status epilepticus after surgery	5	3	1

Table S7: SAEs reported for the first 20 patients.

4.1.8 Primary parameters/end points first 20 patients

Preliminary post-surgical outcome at 6-8 weeks is available in 20 patients, at 6 months in 12 patients, and at 12 months in 6 patients. Seizure free is defined as Engel 1A en 1B (no seizures, but auras may occur). Recurrent seizures are defined as Engel 1C-4.

	**HFO-arm**	**Spike-arm**
**Preliminary 6-8weeks post-OK:**	**N=10**	**N=10**
Auras	3	2
Seizures	2	2
Seizure free	8	9
Recurrent seizures	2	1
**Preliminary 6 months post-OK:**	**N=4**	**N=4**
Auras	2	2
Seizures	2	2
Seizure free	3	3
Recurrent seizures	1	1
**Final 12 months post-OK:**	**N=1**	**N=2**
Auras	0	1
Seizures	1	1
Seizure free	1	1
Recurrent seizures	0	1

Table S8: Preliminary primary outcome first 20 patients.

4.1.9 Secondary parameters/end points

	HFO arm			Spike arm		
**Procedure duration**	**N=10**			**N=10**		
Mean (stdv) # ioECoG recordings (range)	5.5 (±3.0) (3.0-11.0)			6.5 (±2.8) (3.0-12.0)		
Mean (stdv) duration ioECoG recordings (hh:mm:ss) (range)	00:24:50 (±00:10:46) (00:13:34-00:41:23)			00:27:06 (±00:09:50) (00:13:44-00:49:54)		
Mean (stdv) duration surgery recordings (hh:mm:ss) (range)	03:57:54 (±01:08:13) (02:27:00-06:10:00)			04:10:42 (±00:44:10) (03:27:00-05:31:00)		
**Neurological deficits (NIHSS) (score min=0, max=42)**	N=10			N=10		
# patients yes/no deficit pre-NIHSS	1/9			1 /9		
Min/Max pre-surgery NIHSS score	0.0-1.0			0.0-1.0		
# patients yes/no deficit post-NIHSS	3/7			3/7		
Min/Max post-surgery NIHSS score	0.0-1.0			0.0-2.0		
# patients Δ pre-post NIHSS>0	3			2		
Min/Max Δ pre-post NIHSS	-1.0-1.0			0.0-2.0		
**Health related Quality of life (Qol, range 0 – 10)**	**N=10**	**N=6**	**N=3**	**N=10**	**N=6**	**N=3**
Mean (stdv) Qol baseline (range)	6.7 (±1.9) (4.0-10.0)			7.5 (±1.5) (5.0-10.0)		
Mean (stdv) Qol 6-8w post (range)	7.6 (±1.1) (6.0-10.0)			7.6 (±1.5) (4.0-9.0)		
Mean (stdv) Qol 6m post (range)		8.0 (±0.0) (8.0-8.0)			8.2 (±1.0) (7.0-10.0)	
Mean (stdv) Qol 12m post (range)			8.3 (±1.5) (7.0-10.0)			8.3 (±0.6) (8.0-9.0)

Table S9: Preliminary secondary outcome first 20 patients

- *Volume of resected tissue:* A pre-operative MRI is performed in all 20 patients. Post-operative MRIs are performed in 18 patients, and will not be performed in 2 patients. MRI data are collected and will be analyzed near completion of the study.

- *Cognitive functioning* Pre-operative NPO is performed for all 20 patients. Post-operative NPO is performed around 1 year after surgery and thus not available yet for the other patients. Three patients have reached the end of the study now. Post-operative NPO is performed in 2 patients, and will not be performed in 1 patient. NPO data is collected and will be analyzed near completion of the study.

4.1.10 End of study

Three patients have reached the end of the study now.

4.2 IDMC report: 40 patients included and FU of 6-8 weeks (dd 07-04-2017)

4.2.1 Study progression

Date official start of study (first inclusion) is 6-11-2014 in the UMC Utrecht.
Total population in study: 78 patients
Expected inclusion rate: ±45 patients/year (±45/year in UMCU)
Expected inclusion rate ±4 patients/month,
Note: currently the maximal capacity of dedicated team is ±4 patients/month.

4.2.2 Recruitment

In September 2014 the recruitment started in the UMC Utrecht. Since then:

- Approached patients: 75
- Included patients: 42
- 'Pending' patients: 4
- Patients not included, reason: 29
    - Decided not to participate after given information 27
    - Organizational problems 2

In total 21 other patients were identified as eligible patients during screening but were not approached at all because:

- they were not interested in receiving information about the study (n = 11).
- not enough members of the HFO team available at their planned date of surgery (n = 10).

4.2.3 Randomization

So far, in total 42 patients have been randomized. Twenty out of the 42 patients were children.

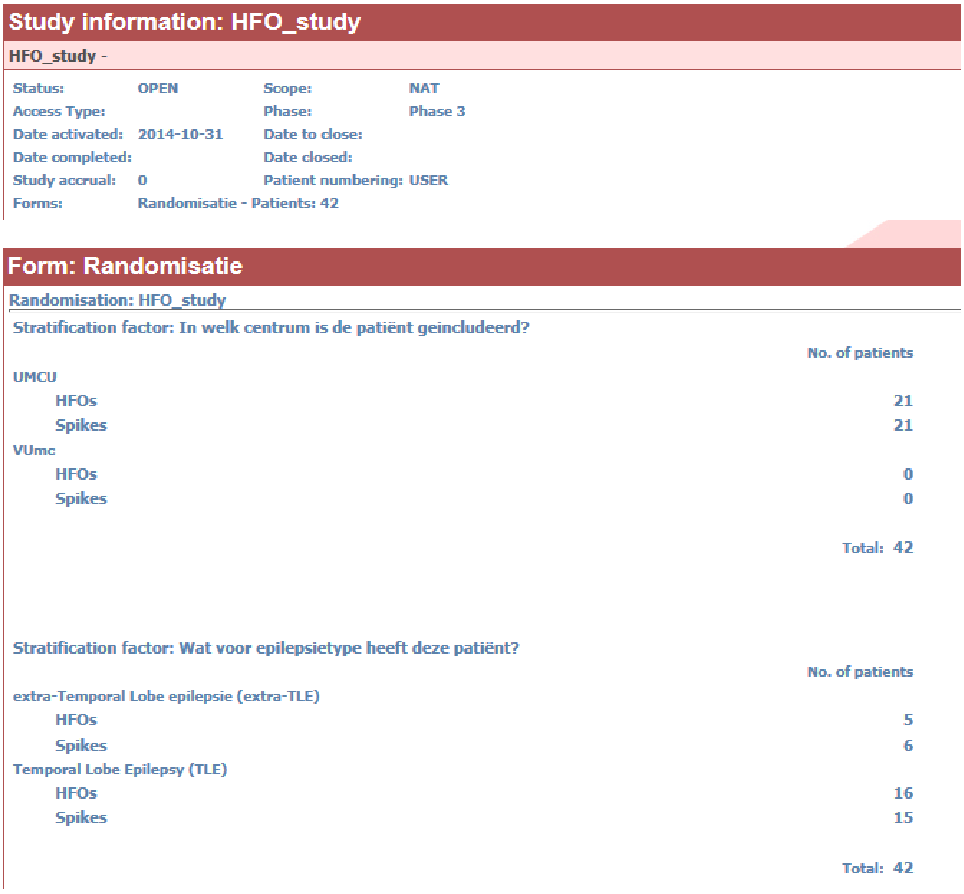

Study information: HFO_study

HFO_study -

Status:	OPEN	Scope:	NAT
Access Type:		Phase:	Phase 3
Date activated:	2014-10-31	Date to close:	
Date completed:		Date closed:	
Study accrual:	0	Patient numbering:	USER
Forms:	Randomisatie - Patients: 42		

Form: Randomisatie

Randomisation: HFO_study

Stratification factor: In welk centrum is de patiënt geincludeerd?

	No. of patients
UMCU	
HFOs	21
Spikes	21
VUmc	
HFOs	0
Spikes	0
Total:	42

Stratification factor: Wat voor epilepsietype heeft deze patiënt?

	No. of patients
extra-Temporal Lobe epilepsie (extra-TLE)	
HFOs	5
Spikes	6
Temporal Lobe Epilepsy (TLE)	
HFOs	16
Spikes	15
Total:	42

Figure S7: ALEA overview randomization first 40 patients.

4.2.4 Protocol violations

No new protocol violations have been added since last DSMB report.

4.2.5 Treatment and follow-up

The total planned follow-up after surgery is 1 year. Additional follow-up questionnaires are assessed at 6-8 weeks, 6 and 12 months after surgery, e.g. to collect preliminary outcome information.

Post-surgical outcome is available for:

- 6-8 weeks: 40 patients
- 6 months: 36 patients
- 12 months: 25 patients

Lost during follow-up: 0

NOTE: From here on, we will report the results of the first 40 included patients of which first preliminary outcome at 6-8 weeks is available.

4.2.6 Baseline characteristics first 40 patients

	**Total (N=40)**	**HFO-arm (N=21)**	**Spike-arm (N=19)**
# Male/Female	19/21	9/12	10/9
# Adult/Child	20/20	13/8	7 /12
Mean + stdev age in years (range)	22.3 ±17.0 (2.0-59.0)	26.2±17.3 (2.0-59.0)	17.9±16.0 (2.0-57.0)
Mean duration epilepsy in months	147.8±171.6 (6.0-662.0)	174.1±181.7 (13.0-662.0)	118.8 ±159.5 (6.0-570.0)
TLE/extra-TLE	17/3	16/5	15/4
Region focus:<br>*-Temporal*<br>*-Frontal*<br>*-Central*<br>*-Parietal*	 31<br>8<br>0<br>1	 16<br>5<br>0<br>0	 15<br>3<br>0<br>1
# Auras/Seizures before surgery	20/36	7/20	13/16
Mean # Auras/month (range)	8.8±12.1 (0.0-45.0)	15.0±16.5 (3.0-45.0)	5.4±7.8 (0-30.0)
Mean # Seizures/month (range)	55.9±122.0 (0.0-600.0)	64.9±144.7 (1.0-600.0)	44.8±89.2 (0.0-336.0)

Table S10: Baseline characteristics first 40 patients.

4.2.7 Serious adverse events first 40 patients

We encountered no complications related to study procedures. So far, four SAEs were reported.

#	Description Event	**Category** *(1=death 2=disability/incapacity 3= life-threatening 4= congenital anomaly/birth defect 5= hospitalization-initial or prolonged 6= required intervention to prevent permanent impairment 7= other)*	**Relation to study treatment** *(1=Unrelated, 2=Unlikely, 3=Possible, 4=Probable, 5=Definite)*	**Outcome of the SAE?** *1= Recovered 2= Recovered with sequelae 3= Continuing 4= Resulted in Death 5= Unknown*
#1	Readmittance (5 days) due to nausea and headache	5	2	1
#2	Status epilepticus after surgery	5	3	1
#3	Temporary neurological deficit left arm without EEG correlate	5	2	1
#4	Subdural hematoma, external hydrocephalus, urosepsis	5	3	1

Table S11: SAEs reported for the first 40 patients.

4.2.8 Primary parameters/end points first 40 patients

Preliminary post-surgical outcome at 6-8 weeks is available in 40 patients, at 6 months in 36 patients, and at 12 months in 25 patients. Seizure free is defined as Engel 1A en 1B (no seizures, but auras may occur). Recurrent seizures are defined as Engel 1C-4.

	**HFO-arm**	**Spike-arm**
**Preliminary 6-8weeks post-OK:**	N=21	N=19
Auras	4	6
Seizures	5	4
Seizure free	18	18
Recurrent seizures	3	1
**Preliminary 6 months post-OK:**	N=18	N=18
Auras	3	4
Seizures	5	2
Seizure free	14	17
Recurrent seizures	4	1
**Final 12 months post-OK:**	N=13	N=12
Auras	4	5
Seizures	5	3
Seizure free	10	9
Recurrent seizures	3	3
**Latest available follow-up:**	N=21	N = 19
Auras	4	5
Seizures	5	3
Seizure free	18	16
Recurrent seizures	3	3

Table S12: Preliminary primary outcome first 40 patients.

4.2.9 Secondary parameters/end points

	HFO arm			Spike arm		
**Procedure duration**	**N=21**			**N=19**		
Mean (stdv) # ioECoG recordings (range)	5.6 (±2.4) (3.0-11.0)			5.2 (±2.5) (3.0-12.0)		
Mean (stdv) duration ioECoG recordings (hh:mm:ss) (range)	00:27:19 (±00:11:41) (00:13:34-00:50:30)			00:23:16 (±00:08:39) (00:13:01-00:49:55)		
Mean (stdv) duration surgery recordings (hh:mm:ss) (range)	04:13:00 (±01:15:36) (02:27:00-06:38:00)			04:02:22 (±01:00:45) (02:09:00-05:40:00)		
**Neurological deficits (NIHSS) (score min=0, max=42)**						
# patients yes/no deficit pre-NIHSS	4/17			2 /17		
Min/Max pre-surgery NIHSS score	0.0-1.0			0.0-1.0		
# patients yes/no deficit post-NIHSS	8/13			5/14		
Min/Max post-surgery NIHSS score	0.0-3.0			0.0-2.0		
# patients Δ pre-post NIHSS>0	9			3		
Min/Max Δ pre-post NIHSS	-1.0-3.0			0.0-2.0		
**Health related Quality of life (Qol, range 0 – 10)**	**N=21**	**N=16**	**N=13**	**N=19**	**N=17**	**N=12**
Mean (stdv) Qol baseline (range)	6.5 (±2.2) (0.0-10.0)			7.6 (±1.7) (5.0-10.0)		
Mean (stdv) Qol 6-8w post (range)	7.8 (±1.2) (6.0-10.0)			7.6 (±1.4) (4.0-10.0)		
Mean (stdv) Qol 6m post (range)		7.9 (±1.5) (3.0-10.0)			8.2 (±1.5) (5.0-10.0)	
Mean (stdv) Qol 12m post (range)			8.1 (±1.7) (4.0-10.0)			8.4 (±1.2) (6.0-10.0)

Table S13: Preliminary secondary outcome first 40 patients

- ***Volume of resected tissue:*** Pre-operative MRI is performed in all 40 patients. In two patients the MRI is performed in a different hospital So far, post-operative MRIs are performed in 26 patients, and will not be performed in 5 patients. MRI data are collected and will be analyzed near completion of the study.

- ***Cognitive functioning:*** Pre-operative NPO is performed for all 40 patients. In three adults no complete IQ-test has been done. Post-operative NPO is performed around 1 year after surgery and thus not available yet for all patients. Twenty-five patients have reached the end of the study now. Post-operative NPO is performed in 12 patients, will not be performed in 1 patient, and still has to be collected for 12 patients.

4.2.10 End of study

Twenty-five patients have reached the end of the study now.

4.3 Efficiency & safety analysis interim analysis 40 patients

Efficiency:

- Number of patients at ≥ 6 – 8 weeks follow-up: 40 (21 HFO / 19 Spikes)
- % patients with Recurrent Seizures within the first 12 months after OK:

  HFO: 14 % (3 out of 21)

  Spikes: 16% (3 out of 19)

Fisher Exact 2-sided: $p = 1.000$ // Fisher Exact 1 sided: $p = 0.619$

Conclusion: No reason to stop the study as $p > 0.003$ (see stopping rules)

Safety:

Conclusion: Until now 2 SAE per study arm (HFO // Spikes)), there is no reason to stop the study.

4.4 IDMC charter

<table>
<tr><th colspan="2">CONTENT</th></tr>
<tr><td colspan="2">4.5 Introduction</td></tr>
<tr><td>Name (and sponsor's ID) of trial plus ISRCTN and/or EUDRACT number</td><td>ID sponsor: University Medical Centre Utrecht
Name trial: Intra-operative detection and localisation of high frequency oscillations in the ECoG to guide epilepsy surgery.
Short name: "The HFO study"
ABRnr: 44527
METC-ID: 13-389</td></tr>
<tr><td>Objectives of trial</td><td>To investigate the outcome of aECoG-guided surgery using HFOs as biomarker for tailoring versus the traditional use of epileptiform spikes as biomarker, by means of a multi-center single-blinded randomized trial.

Primary objective is to investigate whether delineation of the epileptogenic tissue during aECoG-guided surgery using HFOs instead of epileptiform spikes, will lead to an equal (or improved) outcome with respect to postoperative seizure freedom. Independent of the randomisation ictiform spike patterns will always be resected.

Secondary objectives are to see whether the HFO-based, compared to spike-based, aECoG interpretation will lead to differences in the amount of resected tissue that can be spared, the duration of surgery and aECoG recording, surgical complications, neurological deficits, cognitive function, health related quality of life (HRQOL).
Included is a time line of the trial design (see Figure 1).
Involved centers: UMC Utrecht & VUmc Amsterdam</td></tr>
<tr><td>Outline of scope of charter</td><td>The purpose of this document is to describe the roles and responsibilities of the independent DSMB for the "The HFO study", including the timing of meetings, methods of providing information to and from the DSMB, frequency and format of meetings, statistical issues and relationships with other committees.</td></tr>
<tr><td colspan="2">4.6 Roles and responsibilities</td></tr>
<tr><td>Aims of the committee</td><td>"To protect and serve the patients involved in the "The HFO study" trial and to assist and advise the Principal Investigators so as to protect the validity and credibility of the trial."</td></tr>
<tr><td>Terms of reference</td><td>The DSMB should receive and review the progress and recruitment of the trial data and provide advice on the conduct of the trial to the Principal Investigator.
The DSMB should inform the Principal Investigator and the research team if, in their view:
(i) the results are likely to convince a broad range of clinicians, including those supporting the trial and the general clinical community, that one trial arm is clearly indicated or</td></tr>
</table>

**CONTENT**	
	contraindicated, and there was a reasonable expectation that this new evidence would materially influence patient management; or<br>(ii) it becomes evident that no clear outcome will be obtained.
**Specific roles of DSMB**	The specific roles of the DSMB are:<br>• monitor recruitment figures and losses to follow-up<br>• suggest additional data analyses<br>• monitor overall conduct, data quality, including completeness, encourage collection of high quality data<br>• monitor evidence for treatment differences in the main efficacy outcome measures<br>• monitor evidence for treatment harm (e.g. toxicity data, SAEs, deaths)<br>• make recommendation about trial continuation and recruitment of participants or whether recruitment should be terminated either for everyone or for some treatment groups and/or some participant subgroups<br>• perform the pre-planned interim analysis and recommend on continuation of the study accordingly<br>• give recommendations if the assumptions made for sample size calculation prove to be incorrect. The assumptions may pertain to patient recruitment and the incidence of the primary outcome event.<br>• monitor compliance with previous DSMB recommendations<br>• assess the impact and relevance of external evidence.<br><br>It is the sponsor's obligation to inform the DSMB of the concerns regarding the safety of the subjects. Such concerns could refer (unexpected) side effects of the intervention or data from other studies etc.
**4.7 Before or early in the trial**	
**DSMB input into the protocol**	All potential DSMB members have had access to the protocol before agreeing to join the committee. By signing this charter, DSMB members declare to be comfortable with the protocol and to be supportive of the aims and methods of the trial.
**First meeting**	The DSMB will have its first meeting early in the trial, but after the start of recruitment.
**4.8 Composition**	
**Membership of the DSMB**	The members of the DSMB for this trial are:<br>**(1) Erik Gommer *(Clinical Physicist)***<br>***Maastricht University Medical Centre+***<br>***Department of Clinical Neurophysiology***<br>Erik Gommer is a clinical physicist at the department of Clinical Neurophysiology, where he performs research on cerebral hemodynamics and neuromonitoring. He also is responsible for the training of clinical

CONTENT	
	physicist trainees. He is an advisor of the Medical Ethics Assessment Committee regarding medical devices.<br><br>**(2) Paul Hofman (Neuroradiologist)**<br>***Maastricht University Medical Centre+***<br>***Department of Radiology***<br>Paul Hofman is associate professor in neuroradiology at the department of radiology. He has extensive experience with research on imaging biomarkers of cognitive decline in epilepsy patients, trauma patients and patients with neuropsychiatric disorders. His clinical work in in neuroradiology and head and neck radiology with an emphasis on epilepsy imaging.<br><br>**(3) Geert-Jan Rutten (Neurosurgeon)**<br>***St Elisabeth Hospital***<br>***Department of Neurosurgery***<br>Geert-Jan Rutten is a neurosurgeon working at the St Elisabeth Hospital in Tilburg. He specializes in clinical use of functional neuroimaging techniques, brain tumour surgery and cognitive impairments in neurosurgical patients.<br><br>**(4) *Ton Feuth (Biostatistician, CHAIR)***<br>***Radboud University Nijmegen Medical Centre***<br>***Department of Epidemilogy, Biostatistics en Human Technology Assessment (EBH)***<br>Ton Feuth is a retired university teacher biostatics. Besides lecturing he provided statistical support for several biomedical research projects and resulting papers. He is member (methodology) of the METC CMO Nijmegen, and member of the METC Independent Review Board Nijmegen. Currently he is member of the Data Safety Monitoring Board for the study "*Two phase, repeated crossover study with dose escalation on Delta(9)-tetrahydrocannabinol (Delta-THC) in behavioral disturbances in dementia"* (Dept Geriatrics & Alzheimer Centre Nijmegen, As a statistician he performed the interim analysis.<br><br>The members declare that they have no competing interest (Annex 1).
**Responsibilities of the DSMB chair**	The Chair has previous experience of serving on DSMBs and experience of chairing meetings, and facilitates and summarises discussion. The Chair is responsible for ensuring that final recommendations are made and communicated (verbal and in writing) to the Principal Investigator.
**The responsibilities of the DSMB statistician**	The DSMB membership will include a statistician to provide independent statistical expertise, this is Ton Feuth.
**The responsibilities of the trial statistician**	The coordinating investigator of the study (Maryse van 't Klooster) will act as trial statistician and will produce (or oversee the production of) the regular reports to the DSMB. A template report will be agreed upon during the first and second DSMB meeting.

CONTENT	
**The responsibilities of the PI and other members of the Trial Management Group (TMG)**	The PI and coordinating investigator are expected to attend open sessions of the DSMB meeting and update the DSMB on study progress and relevant issues.
**4.9 Relationships**	
**Relationships with Principal Investigators, other trial committees (eg Trial Steering Committee (TSC) or Executive Committee), sponsor and regulatory bodies**	The PI and the coordinating investigator are the primary contacts for the DSMB. The coordinating investigator with respect to more operational matters, the PI as the formal person to whom the DSMB will report its recommendations. The PI accepts the recommendations on behalf of the Sponsor.
**Authority DSMB**	The DSMB provides recommendations to the PI. It is the responsibility of the PI and the Sponsor to respond appropriately to the advice and recommendations of the DSMB.
**Payments to DSMB members**	The chair/statistician, Ton Feuth, will receive a compensation for his work. Other members are not paid, except for reimbursement for travel expenses.
**The need for DSMB members to disclose information about any competing interests**	All DSMB members signed the “Suggested competing interests form” which can be found in annex 1.
**4.10 Organisation of DSMB meetings**	
**Expected frequency of DSMB meetings**	The frequency of the DSMB meetings is scheduled with regards to the interim analysis of primary endpoint surgical outcome. This interim analysis will be performed by the statistician of the DSMB (Ton Feuth) on the first 20 and 40 patients that are included. Extensive monitoring, supported by the interim analysis, will take place in the first year, followed by a yearly visit in the consecutive years. The exact frequency of meetings will depend upon any statistical plans specified and otherwise on trial events.<br><br>The initial frequency of reports to the DSMB will be at least after each 10 included patients. These reports may initiate an earlier meeting of the DSMB. This frequency may be adapted by the DSMB as the trial progresses.
**Organisation of DSMB meetings**	The default format of the meetings is as follows:<br>1. Open session: Introduction, trial status update and any ‘open’ parts of the report.<br>2. Closed session: DSMB discussion of ‘closed’ parts of the report and to independently discuss recommendations.<br>3. Open session: Feedback of DSMB findings to PI, coordinating investigator and other trial staff present.<br><br>The format may be extended if indicated to include more discussion/clarification with trial staff. Debriefing may also take place after the meeting, in case this is more practical. The DSMB may decide to hold meetings by teleconference.

CONTENT	
**4.11 Trial documentation and procedures to ensure confidentiality and proper communication**	
**Intended content of material to be available in open sessions**	Open sessions: Accumulating information relating to recruitment and data quality will be presented. Total numbers of events for the primary outcome measure and other outcome measures may be presented as agreed with the DSMB on the basis of a template report. Progress data should also be presented per participating centre.
**Intended content of material to be available in closed sessions**	Closed sessions: In addition to all the material available in the open session, the closed session material will include access to un-blinded data at group and individual patient level including complication and safety data.<br>A report will be prepared by the trial statistician that present data group comparative as well as individual. The template will be agreed with the DSMB upon early in the trial. Data will be presented as A/B and the unblinding key will be send separately to the DSMB chair.
**Blinding**	DSMB members will be not be blinded for the treatment allocation.
**Access to accumulating data and interim analysis**	The following individuals will see the unblinded accumulating data and interim results:<br>- DSMB members<br>The DSMB reports will not be shared outside this group, only DSMB recommendation will be communicated with all investigators and relevant other groups (e.g. the METC). The DSMB members will not share confidential information as included in the reports with anyone outside the DSMB.
**DSMB decisions/recommendations**	The DSMB reports its recommendations in verbal and in writing to the PI. The PI is responsible for further dissemination. The coordinating investigator will ensure appropriate archiving of DSMB recommendations (In addition to the DSMB own archiving.)
**Availability of reports to the DSMB**	The DSMB will receive updated reports preferably 2 weeks before any meeting, but at least 1 week before. Reports will be sent to the DSMB members via appropriate secured electronic transfer. DSMB members ensure deletion of reports with after 1 week after the recommendations has been send to the PI.
**Confidential material after the meeting**	DSMB members ensure deletion of reports within 1 week after the recommendation has been sent to the PI. The trial statistician is responsible for securely archiving an electronic copy of every final report prepared for the DSMB.
**4.12 Decision making**	
**Decisions/recommendations by the DSMB**	Recommendations can include:<br>• No action needed, trial continues as planned

CONTENT	
	• Early stopping due, for example, to clear harm of a treatment, futility, or external evidence<br>• Suggest protocol changes in view of continued safety of the participants.<br><br>The DSMB has the responsibility to advise the Sponsor and the PI. However, if the Sponsor and PI decide to ignore the DSMBs recommendations, they must provide a written substantiated reason.
**Formal statistical methods**	The DSMB will review primarily the safety of patients in this study. Therefore they review the primary outcome parameter: (preliminary) post-surgical outcome dichotomised into seizure freedom vs. recurrent seizures. Interim analysis is scheduled after inclusion of the first 20 and 40 patients, and will be primarily a safety analysis. (Preliminary) post-surgical outcome will be determined at 6w/6m/12 months. The most recent available post-surgical outcome for each patient will be used during interim analysis.<br><br>The DSMB will monitor differences in the rates of occurrence of (S)AES (see section C1 ‘ The research protocol’ paragraph 9.2) between treatment groups as well as other safety aspects closely. Summaries will be provided such that events can be categorized and compared between treatment groups in a clinical meaningful way. Substantial differences guided by statistical criteria and/or clinical judgment may lead to a recommendation to discontinue the trial for safety reasons, when weighted against potential intervention benefits.<br>Efficiency analysis is performed once during the the interim analysis of 40 patients (halfway inclusion). This requires an alpha correction for the efficiency analysis using the O’Brien Flemming method. The test will be performed with an alpha = 0.003 (one-sided because of the non-inferiority design). Premature termination of the study is possible in case of a significant (p-value <0.003) harmful effects in patients that obtained seizure freedom in arm 1 (HFOs) compared to arm 2 (spikes, current standard standard) at interim analysis’.
**Decision/ recommendation process**	The DSMB will make every effort to reach a unanimous decision. All material decisions and recommendations require attendance and participation of all three members.
**4.13 Reporting**	
**DSMB recommendations/decisions**	The DSMB will confirm recommendation in writing to the PI within 1 week of the meeting. A copy of this is lodged with the trial office.
**Minutes of the meetings and archiving**	The coordinating investigator is responsible for ensuring minutes are taken and archived for the open sessions. The DSMB chair is responsible for ensuring minutes are taken for the closed session. The chair will archive these minutes, which will be made available to the PI after completion of the trial (after database lock and unblinding). The DSMB Chair should sign off any minutes or notes.

CONTENT	
**Disagreement between the DSMB and the body to which it reports**	If the DSMB has serious problems or concerns with the decision by the PI or the Sponsor following a material DSMB recommendation, this will first be discussed directly with the PI and responsible department management. If no agreement can be reached, this will be escalated to the responsible representative of the board for the directors of the UMCU.<br><br>Depending on the reason for the disagreement confidential data may have to be revealed to all those attending such meetings. The meetings should be chaired by a senior member of the responsible department, the board of directors or an external expert who is not directly involved in the trial.
**4.14 After the trial**	
**Publication of results**	At the end of the trial the DSMB is willing to discuss the final data with the trial investigators (e.g. PI, coordinating investigator, and other trial staff) and give advice about data interpretation.
**The information about the DSMB that will be included in published trial reports**	DSMB members should be named and their affiliations listed in the main report, unless they explicitly request otherwise. A brief summary of the timings and conclusions of DSBM meetings should be included in the body of this paper.
**Constraints on DSMB members divulging information about their deliberations after the trial has been published**	The DSMB will not pro-actively discuss issues from their involvement in the trial publicly. In any case they will not do so until after the primary trial results have been published, or when permission is agreed with the overseeing committee. An exception might occur in case of serious safety concerns, but this will then first be addressed with the PI according to the stepwise approach indicated above under “Disagreement”.

Signed:

[Name] ________________________ Date: ___/___/___

[Name] ________________________ Date: ___/___/___

[Name] ________________________ Date: ___/___/___

[Name] ________________________ Date: ___/___/___

5 Characteristics non-participants

In total 96 eligible candidates did not participate in the trial. Forty were not approached and fifty-six patients withheld consent.

**Non Participants (N=96)**	
Gender (M/F) N (%)	45 (47%) / 51 (53%)
Child/Adult N (%)	51 (53%) / 45 (47%)
Age at surgery mean (y)	21.3
- Child	10.6
- Adult	33.0
TLE/eTLE N (%)	57 (59%) / 39 (41%)
Surgical side (Left/Right) N (%))	45 (47%) / 51 (53%)
Re-surgery N (%)	12 (13%)

Table S2: Characteristics non-participants (N=96). Note: It was only allowed to gather general pre-surgical baseline characteristics collected during the screening phase for non-participants**.**

6 Additional analyses

6.1 Baseline characteristics

	HFO-arm (N=39)	Spike-arm (N=39)	p-value
**Sex**			
Female/Male	18 (46%) / 21 (54%)	20 (51%) / 19 (49%)	0·82
**Age at surgery (years)**			
Median [IQR]	21 [12-39]	15 [9·2-29]	0·23
Child/Adult	17 (44%) / 22 (56%)	22 (56%) / 17 (44%)	0·37
**Epilepsy duration (years)**			
Median [IQR]	10 [2·8-22]	7.9 [2·2-14]	0·35
**Auras (last month/per month)**			
Yes/no [N patients (%)]	13 (33%) / 26 (67%)	23 (59%) / 16 (41%)	0·004
Median [IQR]	10 [4·0-15]	5·0 [2·0-22]	0·52
**Bilateral tonic-clonic seizures**			
Yes [N patients (%)]	13 (33%)	11 (28%)	0·81
**Seizures (last month/per month)**			
Yes/no [N patients (%)]	38 (97%) / 1 (3%)	36 (92%) / 3 (8%)	0·62
Median [IQR]	12 [3·0-75]	15 [4·0-80]	0·74
**Number of AED trials**			
<2	8 (21%)	15 (38%)	0·14
Median [IQR]	2·0 [2·0-2.5]	2·0 [1·0-3·0]	0·43
**Number of AED used at intervention**			0.14
0	0 (0%)	2 (5%)	
**1**	8 (21%)	13 (33%)	
**2**	21 (54%)	11 (28%)	
**3**	6 (15%)	10 (26%)	
**4**	3 (8%)	3 (8%)	

**5**	1 (3%)	0 (0%)	
**Neurosurgeon**			
**A/B/C**	14/21/4	11/22/6	0·68
**Side surgery**			
Left/right	19 (49%) / 20 (51%)	19 (49%) / 20 (51%)	1·00
**Lobe surgery**			0·12
Frontal	14 (36%)	10 (26%)	
Fronto-temporo-parietal	0 (0%)	1 (3%)	
Parietal	0 (0%)	4 (10%)	
Temporal	24 (62%)	24 (62%)	
Anterior Lobectomy	*14 (36%)*	*15 (38%)*	
Complete Lobectomy	*2 (5%)*	*2 (5%)*	
Neocortical Resection	*8 (21%)*	*7 (18%)*	
Temporo-parieto-occipital	1 (3%)	0 (0%)	
**Previous brain surgery**			
Yes/no	5 (13%) / 34 (87%)	1 (3%) / 38 (97%)	0·20
**Number of preoperative investigations**			
>3	15 (38%)	11 (28%)	0·28
Median [IQR]	2·0 [2·0-3·0]	2·0 [2·0-3·0]	0·24
**Preoperative MRI**			0·71
Abnormal	29 (74%)	30 (77%)	
Abnormal upon revision	7 (18%)	8 (21%)	
Normal	3 (8%)	1 (3%)	
**Preoperative 7 Tesla MRI done**			
Yes/no	8 (21%) / 31 (79%)	4 (10%) / 35 (90%)	0·35
**Preoperative investigations (concordant/(partially) discordant with resection/not performed)**			
MRI	30 (77%) / 9 (23%) / 0 (0%)	34 (87%) / 5 (13%) / 0 (0%)	0·38
Interictal EEG	23 (59%) / 16 (41%) / 0 (0%)	26 (67%) / 11 (28%) / 2 (5%)	0·25
Ictal EEG	22 (56%) / 9 (23%) / 8 (21%)	25 (64%) / 7 (18%) / 7 (18%)	0·82

PET	4 (10%) / 6 (15%) / 29 (74%)	4 (10%) / 5 (13%) / 30 (70%)	1·00
SPECT	2 (5%) / 0 (0%) / 37 (95%)	1 (3%) / 1 (3%) / 37 (95%)	1·00
MEG	4 (10%) / 2 (5%) / 33 (85%)	1 (3%) / 3 (8%) / 35 (90%)	0·59
**Pathology$^{\beta}$**			0·03
“Good” prognosis	25 (64%)	34 (87%)	
Tumour	*10 (26%)*	*16 (41%)*	
Vascular malformation	*4 (10%)*	*3 (8%)*	
Hippocampal sclerosis	*2 (5%)*	*6 (15%)*	
FCD II	*9 (23%)*	*9 (23%)*	
“Poor” prognosis	14 (36%)	5 (13%)	
Gliosis; reactive tissue	*6 (15%)*	*1 (3%)*	
Tuber	*5 (13%)*	*1 (3%)*	
FCD I and mild MCD	*1 (3%)*	*2 (5%)*	
No Abnormality	*2 (5%)*	*1 (3%)*	

Table S4: Baseline characteristics intention to treat population including statistics. Data are n (%), mean (S) or median (IQR).$^{\beta}$ The definition of pathology prognosis (e.g. "good" and "poor") is based on Lamberink et al. 2020.[1*]

6.2 Uni- & multivariable logistic regression analysis

Variable	Category	Univariable analysis				Multivariable analysis including variables with p univariable < 0.2			
		OR	95% CI for OR		P value	OR	95% CI for OR		P value
			Lower	Upper			Lower	Upper	
**Age at surgery - years**		1.002	0.999	1.004	0.164	0.986	0.921	1.057	0.702
**Age at surgery – adult/child**	Adult	Ref	-	-	Ref	Ref	-	-	Ref
	Child	0.463	0.152	1.411	0.176	0.348	0.036	3.335	0.360
**Epilepsy duration - years**		1.009	0.972	1.048	0.622				
**Number of auras at baseline**		0.957	0.869	1.054	0.368				
**Number of seizures at baseline**		0.995	0.987	1.003	0.252				
**Seizure type**	Other	Ref	-	-	Ref				
	Generalized	0.552	0.181	1.685	0.297				
**Number of AED used at baseline**		0.977	0.569	1.678	0.933				
**Side surgery**	Right	Ref	-	-	Ref				
	Left	1.241	0.423	3.644	0.693				
**Location Epilepsy**	Temporal	Ref	-	-	Ref				
	Extra-temporal	1.156	0.386	3.460	0.795				
**Lobe Surgery**	Temporal	Ref	-	-	Ref				
	Frontal	0.95	0.285	3.161	0.933				
	Parietal	2.533	0.371	17.279	0.342				
**Previous brain surgery**	No	Ref	-	-	Ref	Ref	-	-	Ref
	Yes	4.143	0.754	22.755	0.102	1.449	0.147	14.279	0.751
**Number of pre-operative exams**		0.970	0.553	1.701	0.916				
**Number of pre-operative exams - dichotomous**	Up to 3	Ref	-	-	Ref				
	>3	0.974	0.238	3.979	0.971				
**Pathology - type**	Hippocampal sclerosis	Ref	-	-	Ref				
	Tumour	9637400	0	Inf	0.994				
	Vascular malformation	86736596	0	Inf	0.994				
	FCD II	6802870	0	Inf	0.995				
	Gliosis; reactive tissue	154198393	0	Inf	0.993				
	Tuber	115648794	0	Inf	0.994				
	FCD I and mild MCD	231297589	0	Inf	0.993				
	No abnormality	231297589	0	Inf	0.993				
**Pathology - prognosis**	Good	Ref	-	-	Ref	Ref	-	-	Ref
	Poor	12.146	3.508	42.046	**$8.11*10^{-5}$**	11.892	3.174	44.562	**0.0002**
**Surgeon**	Surgeon 1	Ref	-	-	Ref				
	Surgeon 2	0.629	0.174	2.265	0.478				
	Surgeon 3	1.414	0.307	6.508	0.656				

Table S5: Results of the uni- and multivariable logistic regression analyses of the pre-specified baseline characteristics versus outcome (recurrent seizures).

6.3 Intraclass Correlation Neurosurgeons

We determined the intraclass correlation coefficient (ICC) for the three surgeons in our study to inform us how strongly clustered our primary outcome (i.e. seizure freedom) is. A high ICC means the outcome values are highly clustered, meaning that results depend on the surgeon to which the observations belong. In contrast, a low ICC means the outcome values are minimally or not at all clustered and do not depend on the surgeon.

We calculated the ICC from our regression model's empty or null version.[5] To obtain information on ICC's uncertainty (i.e. its distribution), we used a Bayesian generalized linear mixed model with non-informative priors (Stan, https://mc-stan.org/).

We fitted the data with two chains, 20,000 iterations (2,500 warm-up), and used a Bernoulli distribution which is more efficient than a binomial distribution given the dichotomous nature of our outcome (i.e. seizure freedom: yes/no).

The intraclass correlation coefficient, based on the variances of posterior predicted distribution and conditioned on random effects, was: 0.17 (95% Credible Interval (CI): 0.09– 0.23), indicating a low amount of outcome clustering within surgeons.

The full stanmodel:

```
functions {
}
data {
  int<lower=1> N;                        // total number of observations
  int Y[N];                              // response variable
  // data for group-level effects of ID 1
  int<lower=1> N_1;                      // number of grouping levels
  int<lower=1> M_1;                      // number of coefficients per level
  int<lower=1> J_1[N];                   // grouping indicator per observation
  // group-level predictor values
  vector[N] Z_1_1;
  int prior_only;
}
transformed data {
}
parameters {
  real Intercept;                        // temporary intercept for centered predictors
  vector<lower=0>[M_1] sd_1;   // group-level standard deviations
  vector[N_1] z_1[M_1];                  // standardized group-level effects
}
transformed parameters {
  vector[N_1] r_1_1;                     // actual group-level effects
  real lprior = 0;                       // prior contributions to the log posterior
  r_1_1 = (sd_1[1] * (z_1[1]));
  lprior += student_t_lpdf(Intercept | 3, 0, 2.5);
  lprior += student_t_lpdf(sd_1 | 3, 0, 2.5)
    - 1 * student_t_lccdf(0 | 3, 0, 2.5);
}
model {
  // likelihood including constants
  if (!prior_only) {
    // initialize linear predictor term
    vector[N] mu = Intercept + rep_vector(0.0, N);
    for (n in 1:N) {
      // add more terms to the linear predictor
```

[5] Conform: S. Wu, C.M. Crespi, and W.K. Wong. Comparison of Methods for Estimating the Intraclass Correlation Coefficient for Binary Responses in Cancer Prevention Cluster Randomized Trials. Contemp Clin Trials. 2012 Sep; 33(5):869-80. doi: 10.1016/j.cct.2012.05.004.

```
    mu[n] += r_1_1[J_1[n]] * Z_1_1[n];
   }
   target += bernoulli_logit_lpmf(Y | mu);
  }
  // priors including constants
  target += lprior;
  target += std_normal_lpdf(z_1[1]);
}
generated quantities {
  // actual population-level intercept
  real b_Intercept = Intercept;
}
```

6.4 Per protocol analyses

Per protocol analyses were performed, considering the patients who showed overruling ictiform spike patterns without HFOs in the HFO group (N=4, see figure 1 [PP]) as patients who could be viewed as being treated as if they were in the spike arm (See figure SX) . Note that in these patients sporadic spikes were not considered. See figure S5 for results.

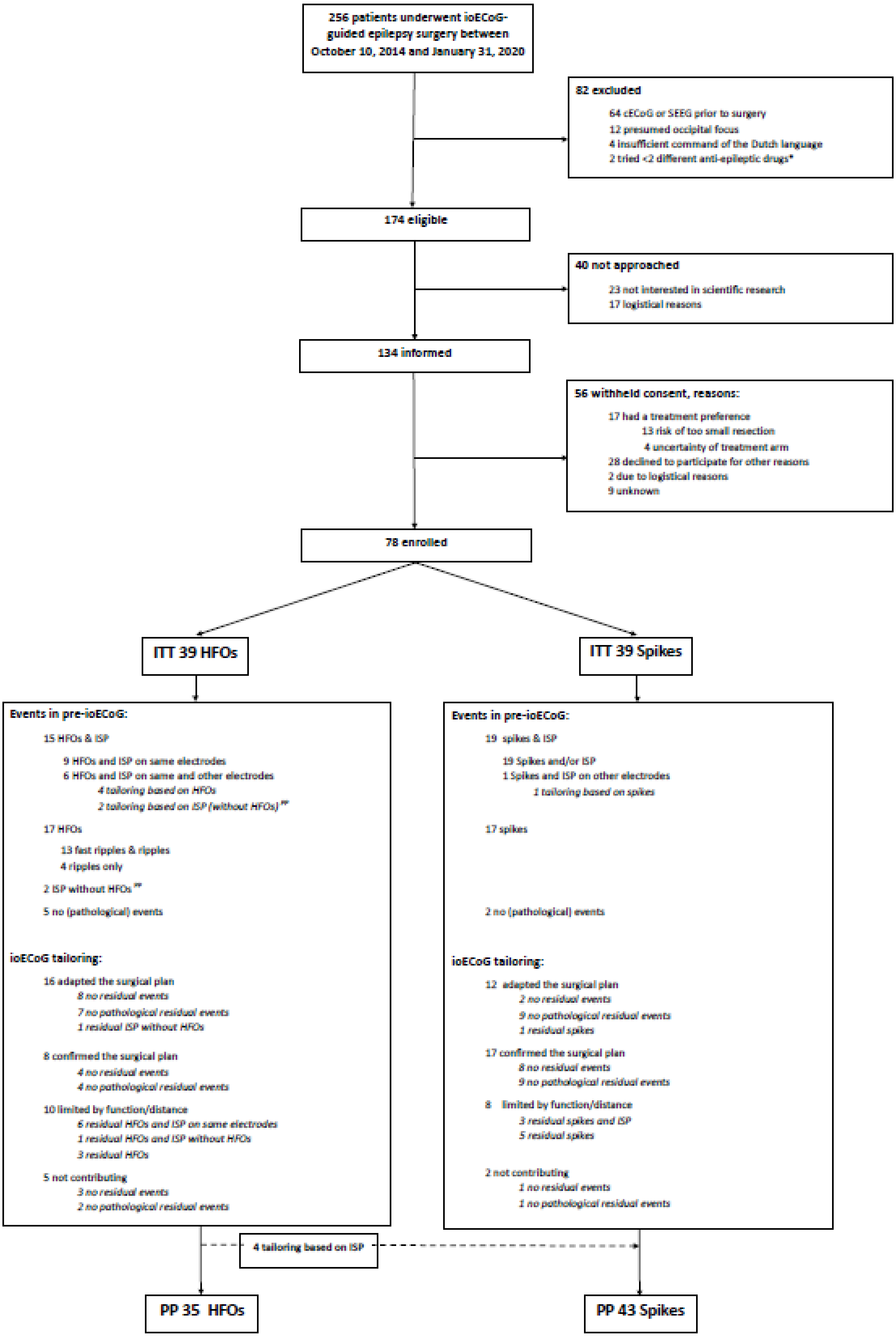

Figure S4: Trial profile including per protocol group. ioECoG = intra-operative electrocorticography (ECoG); cECoG = chronic ECoG; SEEG = stereo-electroencephalography; Events in pre-ioECoG = epileptogenic events recorded during the recordings that were performed before the first resection (pre-resection ioECoG, pre-ioEoG); ioECoG tailoring = all events recorded with ioECoG during the surgical procedure and their impact on the surgical decision making. Residual events = events in the last ioECoG recording after finalizing the resection that remained behind; HFOs = high frequency oscillations,

including both ripples (80-2050Hz) and fast ripples (250-500Hz); ISP = ictiform spike patterns. No events = no epileptogenic events recorded in ioECoG. No pathological events = the events recorded in the ioECoG that are not considered pathological (e.g. prolonged ripple activity not clearly standing out from the background, sharp waves, sporadic spikes, irritative post-resection spikes); [PP] per protocol analyses.

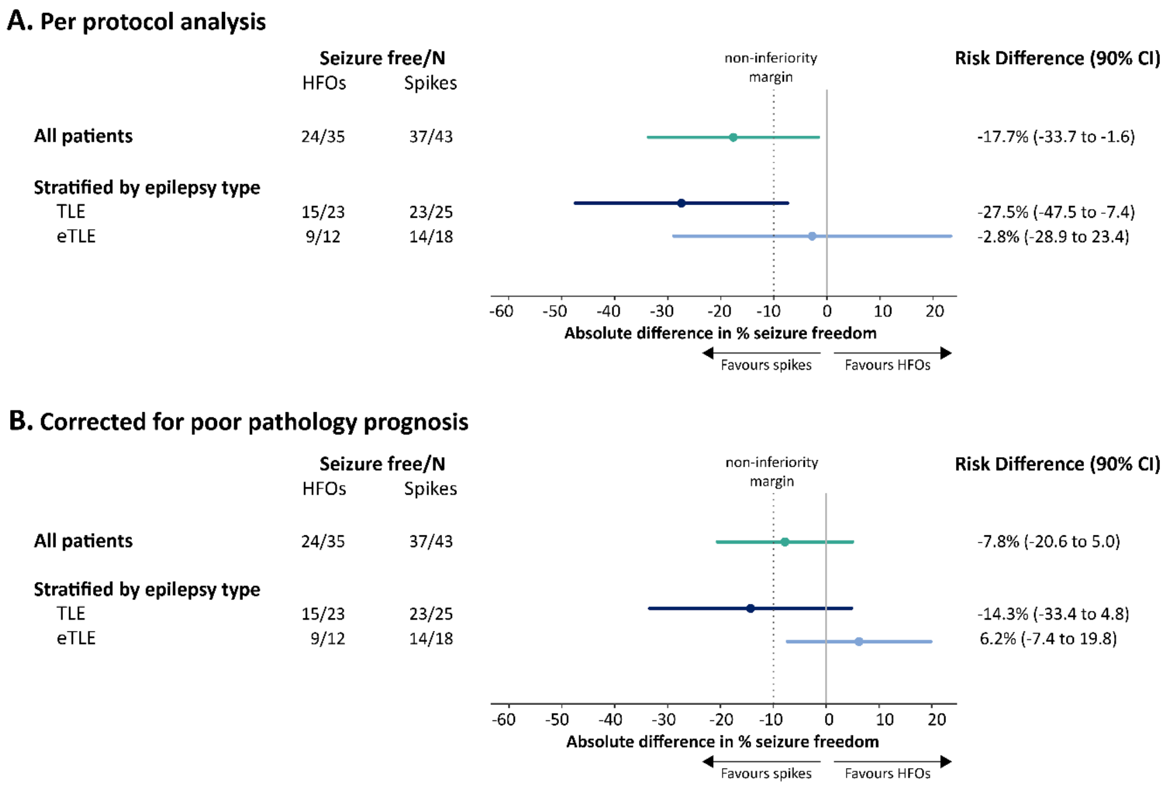

Figure S5: Forest plot of the per protocol analyses including subgroup analyses. Patients in the HFO arm showing overruling ictiform spike patterns without HFOs (N=4) were considered as treated in the spike arm. Results are absolute risk differences (dots) with a 90% CI (line) for the primary endpoint seizure freedom. Non-inferiority of HFOs would be shown if the lower limit of the 90% CI of the absolute risk difference was higher than the -10% non-inferiority margin (dotted line); this is only the case for the corrected subgroup analysis of extratemporal epilepsy type.

6.5 Per-patient ECoG information

Sex	Age (yr)	Group	Side	Lobe	Events in pre-ioECoG	Tailoring based on	Events in post-ioECoG	Type of tailoring	Outcome
f	9,3	HFOs	L	TLE	FR and ripples; ISP without HFOs	HFOs	no events	confirmed the surgical plan	SF
f	24,4	Spikes	R	TLE	ISP + other spikes	ISP + other spikes	no pathological events	adapted the surgical plan	RS
f	2,1	Spikes	R	TLE	no pathological events	surgical plan	no events	not contributing	SF
m	4,4	Spikes	R	eTLE	ISP	ISP	no pathological events	confirmed the surgical plan	RS
m	23,8	HFOs	L	TLE	FR and ripples	HFOs	no events	confirmed the surgical plan	SF
m	13,4	HFOs	L	eTLE	HFOs in ISP	HFOs in ISP	no pathological events	confirmed the surgical plan	SF
f	28,6	HFOs	L	TLE	ripples	HFOs	no events	adapted the surgical plan	SF
f	3,1	Spikes	R	TLE	spikes	spikes	no pathological events	confirmed the surgical plan	SF
m	12,3	Spikes	R	eTLE	ISP + other spikes	ISP + other spikes	no pathological events	adapted the surgical plan	SF
f	30,3	HFOs	R	TLE	FR and ripples	HFOs	no pathological events	adapted the surgical plan	RS
f	18,7	HFOs	L	TLE	FR and ripples; ISP without HFOs	HFOs	no events	adapted the surgical plan	SF
f	13,5	Spikes	R	TLE	ISP + other spikes	ISP + other spikes	no events	confirmed the surgical plan	SF
f	14,0	HFOs	L	TLE	no (pathological) events	surgical plan	no events	not contributing	SF
f	13,2	Spikes	L	TLE	ISP + other spikes	ISP + other spikes	spikes	limited by function/distance	SF
m	10,6	Spikes	L	TLE	spikes	spikes	no pathological events	confirmed the surgical plan	SF
f	31,6	Spikes	R	TLE	ISP + other spikes	ISP + other spikes	no pathological events	adapted the surgical plan	SF
f	15,4	HFOs	R	TLE	FR and ripples	HFOs	no events	adapted the surgical plan	RS
m	20,6	Spikes	R	TLE	spikes	spikes	no pathological events	confirmed the surgical plan	SF
f	14,0	HFOs	R	TLE	FR and ripples; HFOs in ISP	HFOs + HFOs in ISP	FR and ripples; HFOs in ISP	limited by function/distance	SF
m	22,0	HFOs	L	TLE	FR and ripples	HFOs	no pathological events	confirmed the surgical plan	SF
m	5,6	HFOs	R	eTLE	HFOs in ISP; ISP without HFOs	ISP without HFOs	FR and ripples; HFOs in ISP	limited by function/distance	SF
m	6,5	Spikes	L	eTLE	ISP	ISP	ISP	limited by function/distance	RS
m	49,9	HFOs	L	eTLE	no pathological events	surgical plan	no pathological events	not contributing	RS
f	2,2	HFOs	R	TLE	FR and ripples	HFOs	no events	adapted the surgical plan	SF
m	12,8	Spikes	R	TLE	spikes	spikes	no events	confirmed the surgical plan	SF
m	19,9	Spikes	L	eTLE	spikes	spikes	no pathological events	confirmed the surgical plan	SF
f	8,9	Spikes	R	TLE	spikes	spikes	no pathological events	confirmed the surgical plan	SF
f	49,2	HFOs	L	TLE	FR and ripples; HFOs in ISP	HFOs	HFOs in ISP	limited by function/distance	RS

m	53,2	Spikes	R	TLE	ISP + other spikes	ISP + other spikes	no events	confirmed the surgical plan	SF
m	4,6	Spikes	R	TLE	spikes	spikes	no events	adapted the surgical plan	SF
f	57,3	Spikes	L	TLE	spikes	spikes	no events	confirmed the surgical plan	SF
m	44,9	HFOs	L	TLE	no pathological events	surgical plan	no events	not contributing	RS
f	59,2	HFOs	R	TLE	ripples	HFOs	no events	confirmed the surgical plan	SF
f	7,6	HFOs	R	eTLE	no pathological events	surgical plan	no events	not contributing	SF
m	13,4	Spikes	R	TLE	ISP + other spikes	ISP + other spikes	no pathological events	adapted the surgical plan	SF
f	49,6	HFOs	R	TLE	FR and ripples; ISP without HFOs	HFOs	no events	adapted the surgical plan	SF
m	41,1	HFOs	L	eTLE	ISP without HFOs	ISP without HFOs	no events	adapted the surgical plan	RS
f	36,8	Spikes	R	TLE	spikes	spikes	no events	confirmed the surgical plan	SF
m	44,0	HFOs	R	TLE	FR and ripples	HFOs	no pathological events	adapted the surgical plan	RS
m	18,6	HFOs	R	TLE	FR and ripples; ISP without HFOs	ISP without HFOs	FR and ripples	limited by function/distance	SF
f	25,5	Spikes	L	eTLE	ISP	ISP	no pathological events	adapted the surgical plan	SF
f	25,9	Spikes	R	eTLE	ISP	ISP	no events	confirmed the surgical plan	SF
m	15,2	Spikes	L	TLE	spikes	spikes	spikes	adapted the surgical plan	SF
m	9,4	Spikes	L	TLE	spikes	spikes	spikes	limited by function/distance	SF
m	36,0	HFOs	R	TLE	HFOs in ISP	HFOs in ISP	HFOs in ISP	limited by function/distance	RS
m	0,6	Spikes	L	eTLE	ISP + other spikes	ISP	spikes	limited by function/distance	SF
f	13,2	Spikes	L	TLE	spikes	spikes	spikes	limited by function/distance	SF
f	53,7	Spikes	L	TLE	ISP + spikes	ISP + other spikes	ISP	limited by function/distance	SF
m	8,9	HFOs	L	TLE	FR and ripples; ISP without HFOs	HFOs	FR and ripples; ISP without HFOs	limited by function/distance	RS
m	21,4	HFOs	L	eTLE	FR and ripples	HFOs	no pathological events	adapted the surgical plan	SF
m	2,0	HFOs	L	eTLE	FR and ripples; HFOs in ISP	HFOs + HFOs in ISP	HFOs in ISP	limited by function/distance	SF
f	28,9	HFOs	R	eTLE	HFOs in ISP	HFOs in ISP	no pathological events	adapted the surgical plan	SF
m	6,9	HFOs	R	TLE	HFOs in ISP	HFOs in ISP	no pathological events	adapted the surgical plan	SF
m	50,3	HFOs	R	TLE	FR and ripples; HFOs in ISP	HFOs + HFOs in ISP	no events	adapted the surgical plan	SF
m	1,3	Spikes	R	TLE	ISP + other spikes	ISP + other spikes	no pathological events	adapted the surgical plan	SF
m	14,6	Spikes	R	TLE	spikes	spikes	no pathological events	confirmed the surgical plan	SF
m	54,9	Spikes	L	eTLE	ISP + other spikes	ISP + other spikes	no events	confirmed the surgical plan	SF
m	47,0	HFOs	R	TLE	ripples	HFOs	no pathological events	confirmed the surgical plan	SF
f	25,9	Spikes	L	eTLE	no pathological events	surgical plan	no pathological events	not contributing	SF
f	1,0	HFOs	R	TLE	FR and ripples	HFOs	HFOs in ISP	limited by function/distance	RS
f	36,5	Spikes	L	eTLE	ISP	ISP	no pathological events	confirmed the surgical plan	SF

**m**	34,3	Spikes	L	TLE	ISP	ISP	ISP	limited by function/distance	RS
**m**	17,1	HFOs	R	eTLE	FR and ripples	HFOs	FR and ripples	limited by function/distance	SF
**m**	11,6	HFOs	R	eTLE	FR and ripples	HFOs	no pathological events	confirmed the surgical plan	SF
**f**	17,2	HFOs	L	eTLE	FR and ripples; HFOs in ISP	HFOs + HFOs in ISP	no pathological events	adapted the surgical plan	RS
**f**	8,1	Spikes	R	eTLE	ISP + other spikes	ISP + other spikes	no pathological events	adapted the surgical plan	SF
**f**	30,1	HFOs	L	eTLE	ISP without HFOs	ISP without HFOs	ISP without HFOs	adapted the surgical plan	RS
**f**	11,2	Spikes	L	eTLE	spikes	spikes	no events	confirmed the surgical plan	SF
**f**	25,3	Spikes	L	eTLE	ISP	ISP	no pathological events	adapted the surgical plan	SF
**m**	33,0	HFOs	R	eTLE	FR and ripples	HFOs	no events	confirmed the surgical plan	RS
**m**	6,9	Spikes	R	eTLE	ISP + other spikes	ISP + other spikes	no pathological events	adapted the surgical plan	SF
**f**	33,4	Spikes	R	eTLE	spikes	spikes	no events	adapted the surgical plan	SF
**m**	58,9	HFOs	L	TLE	FR and ripples	HFOs	FR and ripples	limited by function/distance	SF
**m**	36,4	Spikes	L	TLE	spikes	spikes	spikes	limited by function/distance	SF
**f**	10,4	HFOs	L	eTLE	ripples	HFOs	no pathological events	adapted the surgical plan	SF
**f**	16,3	Spikes	L	TLE	spikes	spikes	no pathological events	confirmed the surgical plan	SF
**m**	21,3	HFOs	L	TLE	no pathological events	surgical plan	no pathological events	not contributing	SF
**f**	12,3	HFOs	R	eTLE	FR and ripples	HFOs	no events	adapted the surgical plan	SF

Table S6 per-patient ECoG information. f=female m=male R=right, L=left, TLE=temporal lobe epilepsy eTLE=extra-temporal lobe epilepsy, FR=fast ripples, ISP=ictiform spike pattern SF=seizure free, RS=recurrent seizures
